# Computational Screening of Electroactive Biobased-Phthalimide Molecules for Redox Flow Batteries

**DOI:** 10.1021/acs.joc.5c01283

**Published:** 2026-01-06

**Authors:** Alex S. Moraes, Rafaela Binda da Silva, Murilo A. Dada, Giovanna Tâmega, Luana Cristina Italiano Faria, Raphaella Von Stein, Graziela C. Sedenho, Ernesto C. Pereira, Marco A. B. Ferreira

**Affiliations:** † Department of Chemistry, 67828 Federal Univeristy of São Carlos (UFSCar), São Carlos, São Paulo, CEP 13565-905, Brazil; ‡ 153988 São Carlos Institute of Chemistry, University of São Paulo (USP), São Carlos, SP 13566-590, Brazil

## Abstract

In the search of sustainable materials for energy storage, phthalimide-based compounds have shown great potential as anolytes for redox flow batteries (RFBs). Here, we conducted a high-throughput computational screening of 5,705 phthalimide derivatives, including a strategically designed subset of biobased candidates derived from renewable platform chemicals. Structure–property analyses, grounded in principles of physical organic chemistry, were employed to elucidate key trends related to redox potential, radical stability, and solubility, properties critical to RFB performance. Statistical modeling and clustering analysis further refined the selection of optimal candidates. From these efforts, a promising biobased compound was identified, and a closely related derivative was synthesized via a sustainable Diels–Alder route. Electrochemical characterization revealed quasi-reversible redox behavior, high solubility in acetonitrile, and exceptional cycling stability over 2,000 redox events without chemical degradation. These results underscore the utility of computational strategies in accelerating the discovery of robust, renewable, and high-performance organic materials for next-generation energy storage systems.

## Graphical abstract

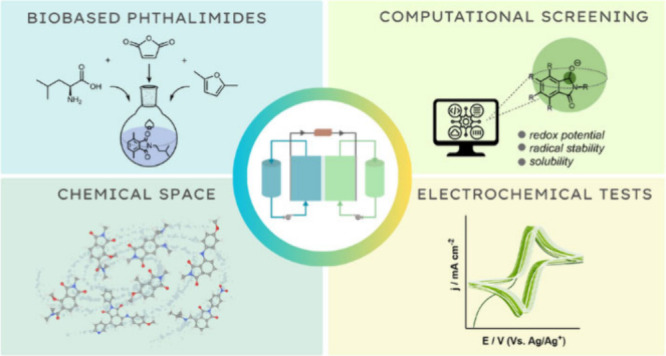

## 1 Introduction

Developing sustainable energy storage solutions is one of the most significant challenges of the 21st century, driven by population growth and ongoing industrialization.^1^ Despite the availability of various renewable energy sources, their integration into the energy grid is still hindered by intermittency.^2^ The integration of energy storage systems that remain stable during these variations will make it easier to incorporate renewables into the electrical system,^3^ and redox flow batteries (RFBs) demonstrate considerable promise for use in large-scale energy storage applications.^4^ In these systems, energy is efficiently stored in solutions of redox-active molecules within external tanks (Scheme 1a). The electrolytes are pumped through an electrochemical cell, where they undergo charging and discharging processes.^5^ In this context, it is crucial to identify the anolytes and catholytes that undergo reversible redox reactions with maximum potential difference which determines the amount of energy that can be stored and released.^6^

**Scheme 1:**
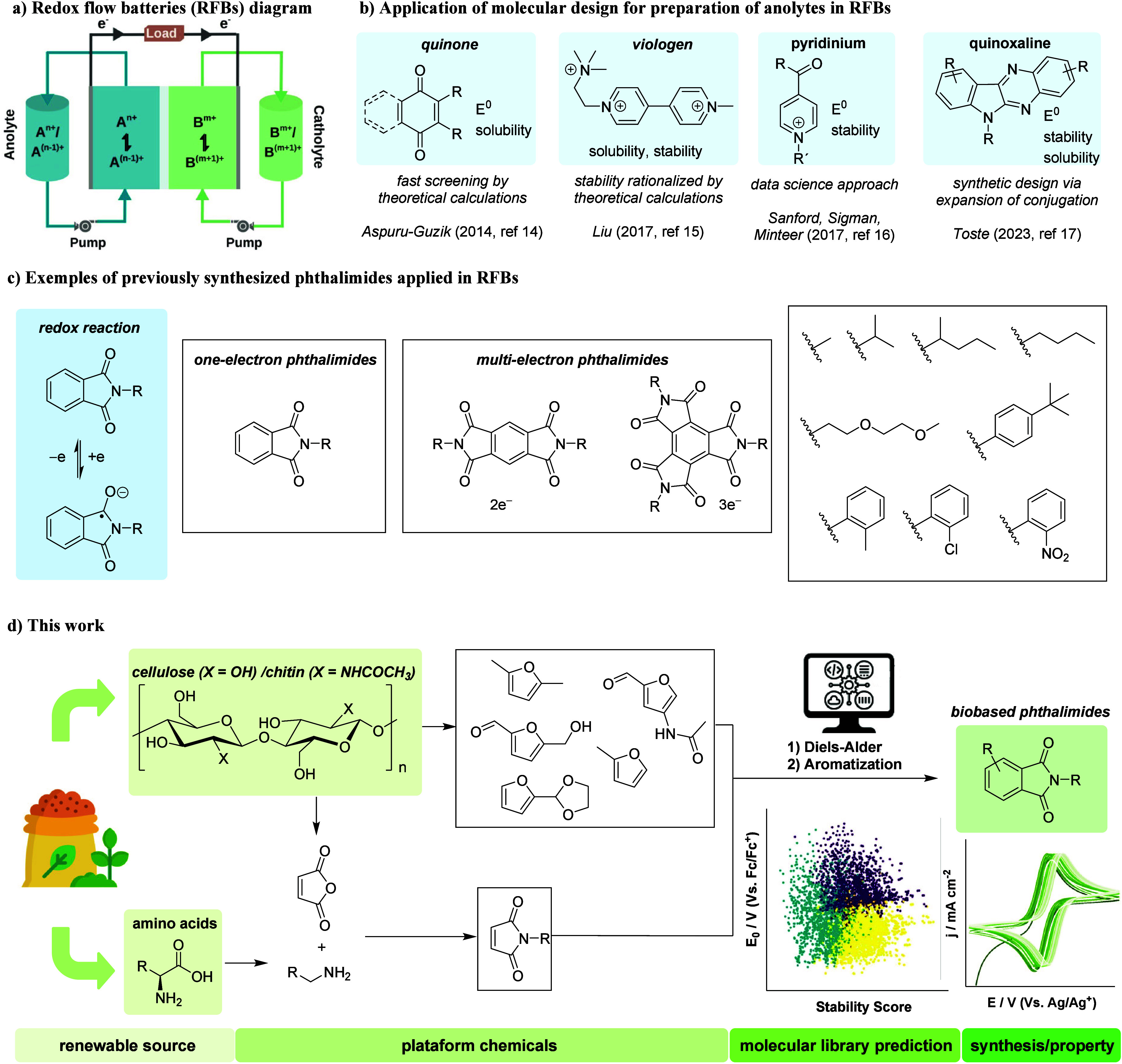
(a) Redox Flow Battery (RFB) Architecture; (b) Representative Anolytes Designed via Molecular Design Strategies; (c) Reported Synthetic Pathways for Phthalimides Applied in RFBs; (d) Overview of This Work: Design of Biobased Phthalimides Guided by Physical Organic Chemistry Principles

The most developed and commercialized RFBs rely on metal ion-based species, such as vanadium, as redox active compounds in strong acidic aqueous solutions. These systems, while promising, present certain drawbacks, including high costs, scarcity, toxicity, and a limited thermodynamic potential window of water (1.6 V),^7^ which reduces energy density and may hinder their large-scale application. The use of Redox-Active Organic Molecules (ROMs), in Non-Aqueous Organic RFBs (NAORFBs), offers a broader electrochemical window, enabling voltages of up to 4 V.^8^ This makes ROMs particularly advantageous for achieving high energy and power densities, although more extreme potentials lead to increased radical reactivity, thereby compromising stability. Identifying electrolytes capable of remaining stable at these extreme potentials remains a critical challenge, especially for anolytes, as most radical formed upon single-electron reduction at potentials below – 2.0 V suffer from poor chemical and electrochemical cycling stability.^9^ By applying the principles of molecular design using physical-organic chemistry tools,^1c,10^ it is possible to modify the molecular structure and properties of ROMs through the implementation of targeted synthetic strategies, facilitating the optimization of key ROMs characteristics, such as redox potentials,^11^ stability,^12^ and solubility.^13^ As a result, in recent times, several anolytes that align with these optimized criteria have been designed, including derivatives of quinone,^14^ viologen,^15^ pyridinium,^16^ quinoxaline,^17^ and phthalimide^18^ (Scheme 1b).

Although recent advances have contributed to progress in the field, organic active materials derived from biomass have primarily been developed for stationary battery applications,^19^ and ROMs specifically designed from abundant and renewable sources for RFBs remain underexplored. Developing such materials would represent a meaningful step toward sustainable energy storage.^1a^ Among the promising candidates are phthalimides, whose electrochemical properties have attracted attention since the 1970s, when early studies reported reversible and irreversible behavior and also potential decomposition pathways, depending on the number of electrons transferred.^20^ These investigations revealed the formation of relatively stable anion radicals in quasi-reversible systems, ultimately motivating their use as redox mediators in electron-transfer reactions.^21^ After their first application as anolytes in NAORFBs,^22^ several studies sought to improve phthalimide performance (Scheme 1c). Strategies included increasing charge density by forming eutectic-based anolytes,^18,19^ enabling multielectron reduction through the introduction of multiple imide groups,^22a^ and designing bipolar redox-active molecules that integrate both anolyte and catholyte functionalities, which help mitigate membrane crossover issues.^11c,22f^ Structural modifications such as N-aryl substitution to shift redox potentials^22c^ and *N*-alkyl chain extension to improve solubility^22g^ have also been explored. Synthesized *N*-alkyl/aryl phthalimides currently exhibit an electrochemical window ranging from – 0.87 to – 1.93 V.^11c,18,22^ However, their use in battery applications has so far been limited to nitrogen-substituted derivatives derived from fossil-based feedstocks. To address this limitation, a potentially green synthetic route employing platform chemicals obtained from cellulose and amino acids could be envisioned, expanding the chemical space of potentially biomass-based electroactive molecules (Scheme 1d). In this approach, a Diels–Alder reaction between biobased maleimides and furans, followed by aromatization, would yield the desired biobased phthalimide structure.^23^

To maintain the focus on sustainability, our study used computational chemistry and data science methods to predict the physicochemical properties and select a potential candidate of biobased phthalimides (Scheme 1d).^10a,24^ To investigate their electrochemical behavior and stability, we designed a tailored chemical space by incorporating functional groups that are either inherent to renewable feedstocks or can be easily introduced via sustainable synthetic routes onto the phthalimide scaffold. The selected and synthesized candidate demonstrated excellent electrochemical performance in preliminary flow battery tests, showing synthetic accessibility, stability, and a favorable electrochemical potential.

## 2 Results and Discussion

### 2.1 Mapping the Chemical Space of Phthalimides

To develop sustainable phthalimide-based redox-active molecules, our approach began with the identification of renewable raw materials as potential feedstocks (Scheme 2a). From this foundation, a range of substituents was selected, including both biobased and fossil-derived groups such as aromatics and strong electron-withdrawing moieties, to enable a systematic comparison of their physicochemical and electrochemical properties. These substituents were chosen based on their presence in biomass or their potential to be introduced via straightforward and sustainable transformations. The core phthalimide scaffold was designed based on a potential Diels–Alder (DA) reaction between furans and maleimides, followed by aromatization.^23^ This method offers a greener alternative to conventional phthalimide synthesis, which typically rely on reactions between phthalic acid derivatives and petroleum-derived amines or anilines.^25^ Notably, furan and maleic anhydride can be obtained from cellulose and chitin, while amino acids can serve as sources of primary amines, making the entire route amenable to renewable sourcing. Once the biobased phthalimide scaffold is in place, diverse postfunctionalization strategies can be envisioned to expand molecular diversity (see in Supporting Information (SI) for details). These derivatizations are compatible with green chemistry principles and allow substitutions at well-defined positions: a total of 14 functional groups (FG) R^1^/R^1^′ and R^2^/R^2^′ on the aromatic ring, while 2 distinct FGs were employed for the R^3^ on the nitrogen atom (Scheme 2b). Alongside nonbiobased substituents, each molecule in the data set consists of the phthalimide scaffold functionalized with one FG at R^3^ and two additional FGs distributed across R^1^, R^1^′, R^2^, and R^2^′. Molecular structures were encoded as SMILES strings, enabling straightforward substitution of FGs at the specified positions. After enumerating all possible combinations, a virtual chemical space comprising 1094 unique biobased phthalimide derivatives were generated, within a total of 5705 molecules. The SMILES representations were then converted to 3D coordinates (XYZ file) using RDKit for subsequent computational analysis. This extensive molecular library enables a comprehensive structure–property relationship analysis and guides the identification of promising candidates for electrochemical applications.

**Scheme 2:**
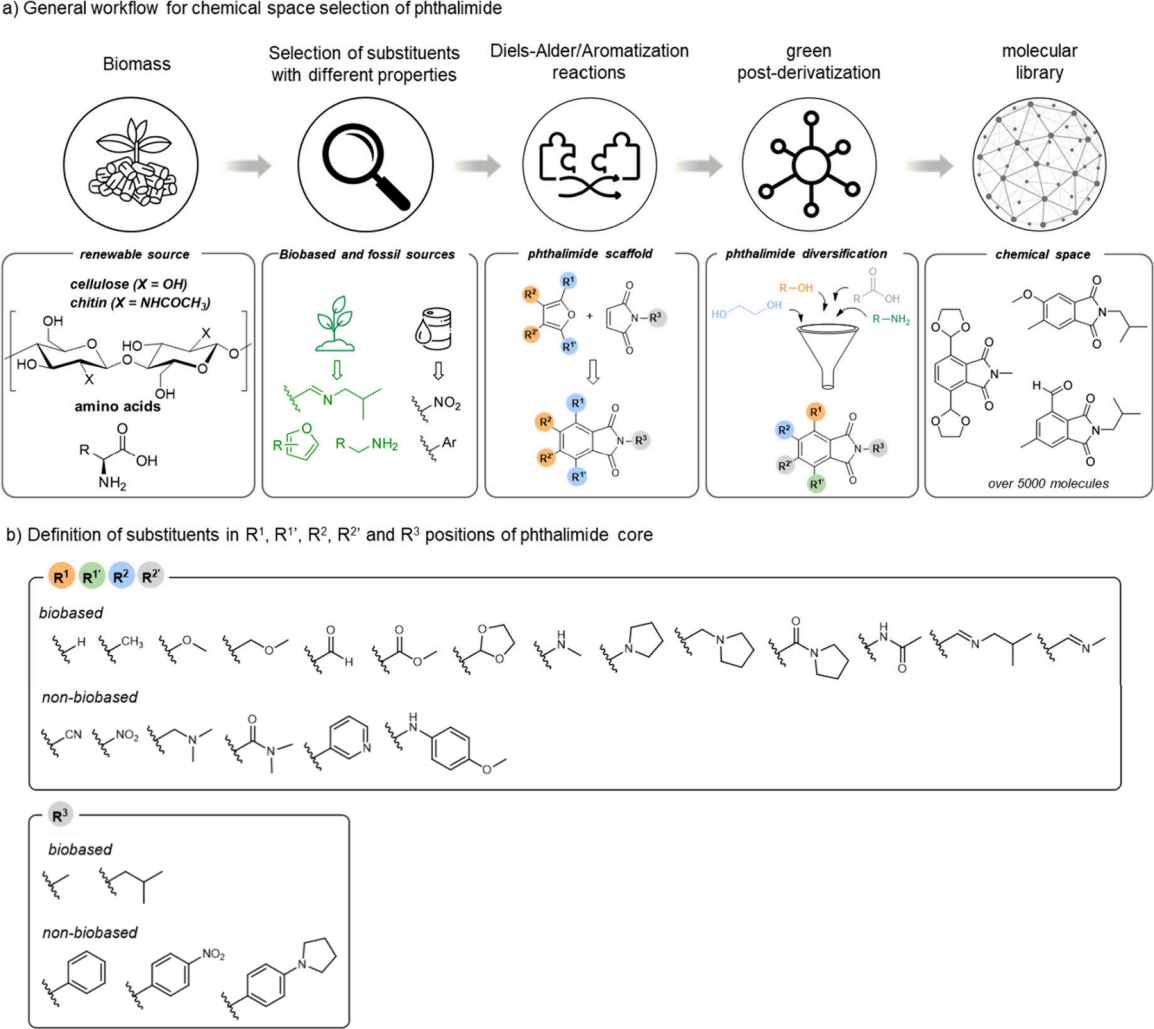
(a) Strategy for Designing Biobased Phthalimides. Biomass-Derived Feedstocks Guide the Selection of Substituents with Diverse Properties;^a^ (b) Substitution Patterns on the Phthalimide Scaffold, with R^1^/R^1^′ and R^2^/R^2^′ on the Aromatic Ring and R^3^ on the Nitrogen Atom

a A Diels–Alder/aromatization reaction sequence yields the phthalimide scaffold, which undergoes green post-functionalization to build a virtual library of over 5000 candidate molecules.

### 2.2 High-Throughput Computational Screening

The computational protocol adopted in this study follows a multistep workflow, summarized in the flowchart shown in Scheme 3. Each step is described in detail in the following sections.

**Scheme 3:**
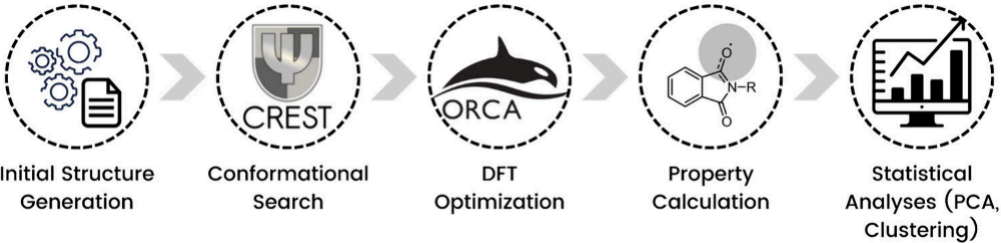
Computational Protocol Adopted in This Study

#### Conformational Sampling and Quantum Chemical Calculations

To ensure proximity to the global energy minimum, an extensive conformational search was performed using CREST at the GFN2-xTB level,^26^ identifying the lowest-energy conformer for each derivative. These structures were further optimized via Density Functional Theory (DFT) calculations using ORCA 5.0.4.^27^ Furthermore, Natural Bond Orbital (NBO) analyses were performed using NBO software, version 7.0.4.^28^ Geometry optimizations and vibrational frequency analyses were conducted at the B97-D3/def2-SVP level, followed by single-point energy evaluations at the higher B97-D3/def2-TZVP level to obtain improved electronic properties, including electronic energies and Mulliken spin densities. This method was chosen after a benchmark test comparing 5 different levels of theory with experimental redox potential (*E*_0_) data. This test is presented in the . All calculations were performed using implicit solvation (acetonitrile) via the conductor-like polarizable continuum model (CPCM). To verify the stability of the optimized geometries, frequency analyses were performed, and 66 molecules exhibiting imaginary frequencies greater than 15 cm^–1^ were discarded. The final data set comprised 5639 phthalimide derivatives, for which all properties were rigorously computed.

#### Properties Calculation

Standard redox potentials were estimated using adiabatic ionization potentials derived from DFT thermochemistry in implicit solvent, including vibrational zero-point energy corrections. For this purpose, the redox potential (*E*_0_) values were calculated according to eq 1, in which the ΔG_red_ and ΔG_ox_ were obtained as a sum of the electronic energy (*E*_el_) from the B97-D3/def2-TZVP single point calculations and the thermal corrections (ΔG_corr_) from the frequency analyses at B97-D3/def2-SVP level, according to eq 2. The latter includes zero-point energy (ZPE) and enthalpy and entropy contributions to the total free energy. The reference electrode potential, *E*_0_^*ref*^, was calculated for ferrocene using the same theoretical approach described for the phthalimide molecules.^29^

$$E_{0}=-\frac{\left(\Delta G_{Red}-\Delta G_{Ox}\right)}{nF}-E_{0}^{Ref}$$ 1

$$\Delta G=E_{el}+\Delta G_{corr}$$ 2

For the stability analyses, we followed Sowndarya et al. protocol,^12^ in which thermodynamic stability and kinetic persistence were evaluated based on the maximum fractional spin density (*FSD*_max_) and the percent buried volume (%*BV*) descriptors. The *FSD*_max_ is a measure of the delocalization of the unpaired electron in the reduced phthalimide radical and can be obtained directly from the DFT calculations. On the other hand, the %*BV* of an atom, which can be defined as the percentage of occupied space inside a sphere centered on the chosen atom, was calculated using Morfeus python package,^30^ which can calculate several molecular features relevant for chemical analyses. Finally, we calculated the stability score (SS), according to the eq 3, in which %BV is calculated at the carbonyl oxygen (O1), as justified in subsequent sections. The stability score is a metric to join the kinetic persistence and the thermodynamic stability into one single parameter. This score has also been used by Hamza et al. as a metric to find N-alkylated pyridoxal stable candidates for AORFBs.^31^

$$Stability\;Score=\%BV_{O1}+50\left(1-FSD_{\max}\right)$$ 3

We defined redox potential, fractional spin density, percent buried volume and stability score as the main features for the selection of suitable candidates for organic stable radical active species redox flow batteries.^10a^ Furthermore, several other properties, such as NBO charges and occupation coefficients, solvation energies, Sterimol parameters, frontier molecular orbitals and electric dipole moments were calculated to perform a deep multidimensional analysis. All properties were extracted and analyzed using in-house python scripts developed exclusively for this work. Despite potential asymmetry in some derivatives (e.g., differing FGs at R^1^ and R^1^′), all properties were treated as symmetric due to the phthalimide core’s inherent symmetry axis, because macroscopic properties will depend on a combination of the effects of both sides of the molecule. To account for this, atom-specific properties (e.g., charges, NBO energies, percent buried volume, Sterimol) were averaged across equivalent positions R^1^ and R^1^′, or R^2^ and R^2^′.

#### Redox Potential

The phthalimide derivatives in our data set span a broad range of redox potentials, from −2.2 V to −0.39 V (vs Fc/Fc^+^), as shown in Figure 1a. The distribution is trimodal, with two distinct peaks and a shoulder near −2.0 V, suggesting the presence of three distinct subpopulations. By deconvoluting the distribution into three Gaussian functions, we classified the molecules into: (i) Group 1 (G1), most negative redox potentials (*E*_0_ ≤ −1.7 *V*); (ii) Group 2 (G2), intermediate potentials (−2.0 V < *E*_0_ < −1.5 V); (iii) Group 3 (G3), least negative potentials (*E*_0_ ≥ −1.5 V).

**Figure 1:**
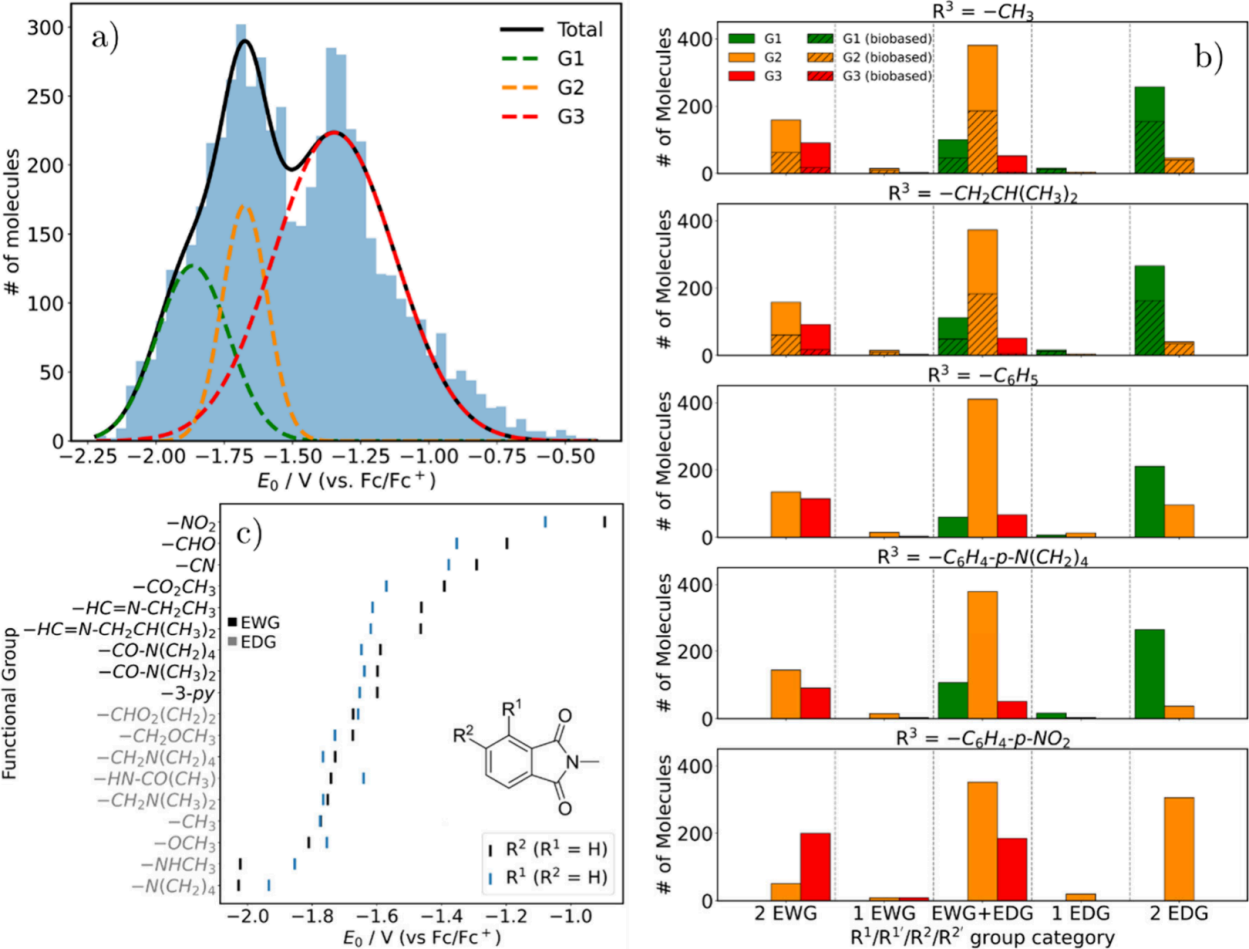
(a) Distribution of computed redox potentials (*E*_0_ vs Fc/Fc^+^) for the phthalimide derivatives. The trimodal profile was deconvoluted into three Gaussian components, corresponding to Groups 1 (G1, most negative), 2 (G2, intermediate), and 3 (G3, least negative). (b) Structural distribution of EDG/EWG substituents at R,^1^ R^2^ and R^3^ across the three groups (G1-G3). (c) Influence of functional group type (EDGs in gray, EWGs in black) and position (R^1^ versus R^2^) on redox potential for monosubstituted molecules. Hammett σ_p_ values were used as a quantitative basis for the classification and ordering of presentation.

To understand the structural basis of these differences, we analyzed the electronic effects of substituents (R^1^ and R^2^) on the phthalimide core (Figure 1b). Data analysis revealed that, among the R^3^ substituents investigated, significant differences in the distribution of groups with respect to the nature of R^1^ and R^2^ substituents were observed only when R^3^ = C_6_H_4_NO_2_. We found that G1 consists almost entirely of molecules with at least one electron-donating group (EDG), while G3 is dominated by molecules with electron-withdrawing groups (EWGs). Notably, no molecule bearing R^3^ = C_6_H_4_NO_2_ was assigned to G1, even when two EDGs were present at R^1^ and R^2^. Mixed substitution patterns (EDG and EWG) lead to intermediate redox potentials (G2). For redox-active anolytes, highly negative potentials are desirable as they increase the cell voltage and energy density of redox flow batteries (RFBs). Thus, G1 molecules, particularly those with strong EDGs, are the most promising candidates. This is probably because EDGs donate electron density to the core of the phthalimide and therefore making the addition of an extra electron more difficult, which then leads to a more negative redox potential.

To further analyze the relative influence of functional groups on redox potential, we focused on molecules bearing a single substitution on the aromatic ring, either at R^1^ or R^2^, while keeping R^3^ = CH_3_. The results are shown in Figure 1c, where EDGs are shown in gray and EWGs in black along the *y*-axis. As previously observed, EDGs tend to shift the redox potential to more negative values than EWGs. Moreover, Figure 1c reveals that strong EDGs (e.g., OR, NR_2_) and all EWGs exhibit a more pronounced variation in redox potential between substitutions at R^1^ and R^2^ when compared to weak EDGs (e.g., CH_3_, CH_2_OR, CH_2_NR_2_). For example, for the NO_2_ and NHCH_3_ substituents, a difference of 0.18 and 0.17 V were observed, respectively, while for OCH_3_ this variation was only 0.06 V.

Beyond the general trends discussed above, a more detailed analysis highlights the positional dependence of substituents on the redox potential of phthalimide derivatives (Figure 1c). The redox process under investigation involves the reduction of the phthalimide core, in which the incoming electron is delocalized over the aromatic ring. Interestingly, not all atomic positions contribute equally to this delocalization: as illustrated in Figure 2, the C-7 position exhibits negligible spin density in the reduced state. Consequently, substituents at R^1^ (attached to C-7) have a limited electronic influence on the redox potential, regardless of whether they are EDGs or EWGs. In contrast, substituents at R^2^ (attached to C-8) are directly associated with a region of significant spin density, resulting in a more pronounced electronic effect. This explains the observed systematic differences between the two positions: strong EDGs at R^2^ shift the redox potential to more negative values compared to the same groups at R^1^, whereas EWGs produce the opposite effect, leading to less negative (more positive) potentials at R^2^ due to their stronger interaction with the spin-delocalized site. Thus, the observed redox shifts can be primarily attributed to positional effects on electronic delocalization, with spin density analysis serving as a qualitative descriptor rather than a direct quantitative predictor of substituent effects.

**Figure 2:**
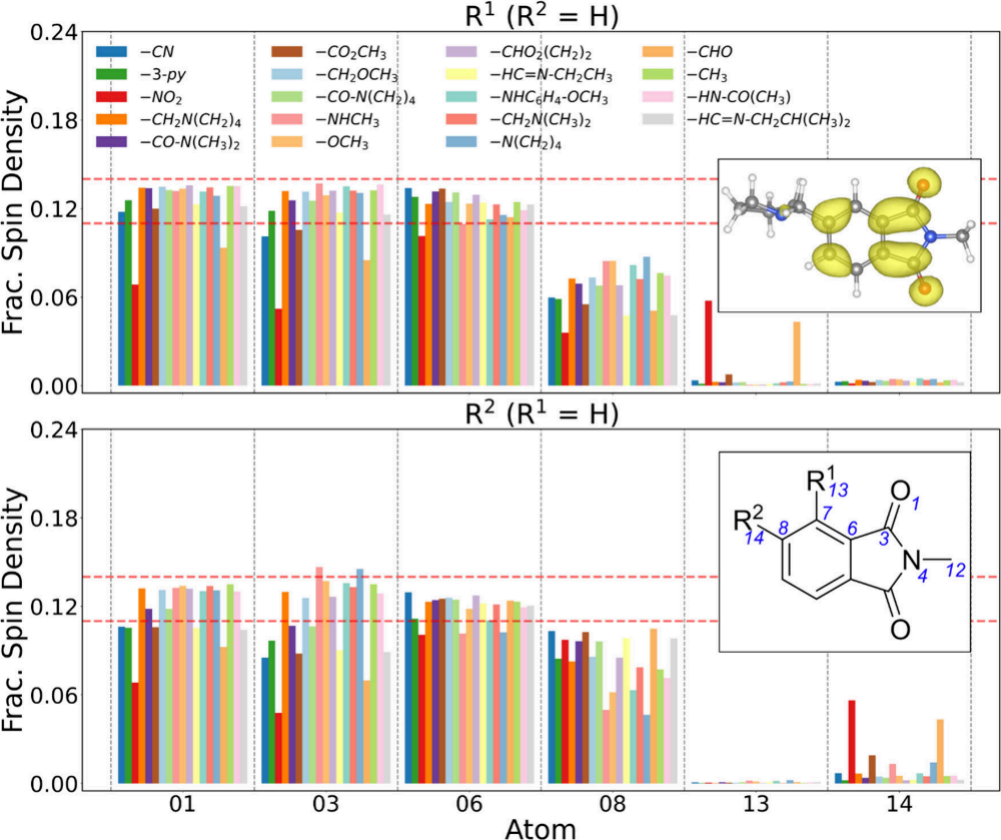
Spin density distributions (FSD) of phthalimide derivatives highlight key atoms (O1, C3, C6, C8) involved in electron delocalization.

#### Thermodynamical Stability and Kinetic Persistence Descriptors

The fact that the *FSD*s (Figure 2) are highly concentrated on atoms O1, C3, and C6 suggests that these atoms are potentially involved in reactive pathways of the reduced radical phthalimides. According to the stability score (eq 3), all three atomic positions should be sterically protected. Indeed, Figure 3a-c shows strong correlations between the %*BV* at these positions, indicating that steric shielding at one site often coincides with relative protection at the others. As expected, C6 exhibits the highest %*BV* due to its position at the ring junction, which effectively captures the bulk of R^1^ and R^2^ substituents. However, considering that the main deactivation pathway for this class of molecules is likely related to the dimerization of the protonated reduced species (Figure 3d),^22^ we focused on the %*BV* at O1 as a practical descriptor of overall kinetic stability, since it highly correlates with C3, which is the atom participating in the dimer formation reaction.

**Figure 3:**
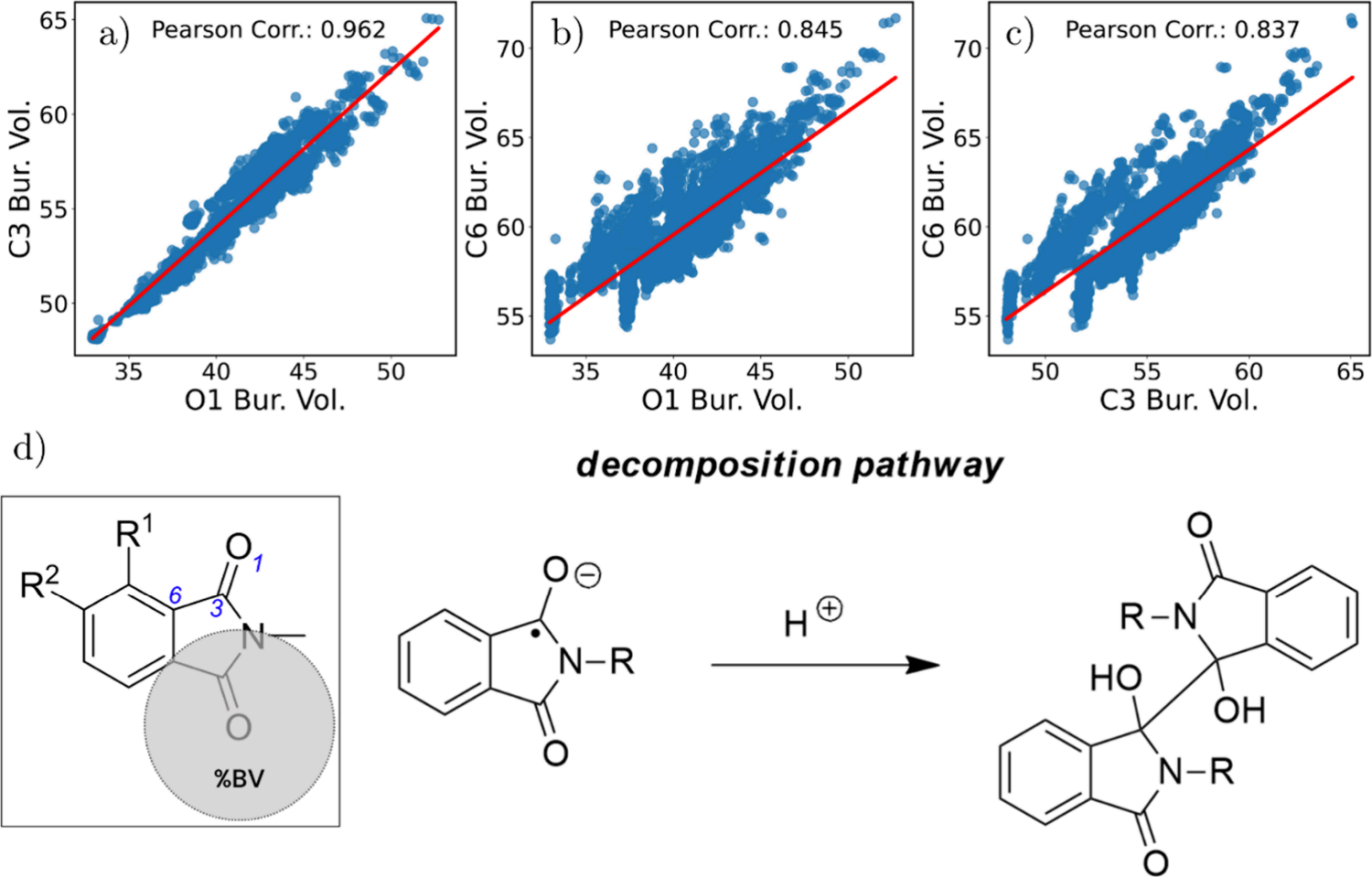
Effect of R-group substitutions on tuning the %*BV* at O1, C3, and C6 atomic positions of different phthalimides. (a) Correlation between %*BV* at atoms O1 and C3. (b) Correlation between %*BV* at atoms O1 and C6. (c) Correlation between %*BV* at atoms C3 and C6. (d) Proposed decomposition pathway via dimerization of the protonated radical species, supporting the use of %*BV* at O1 as a proxy for kinetic persistence.

#### Data Driven Analysis of Chemical Space of Anolyte Candidate

In recent years, data-driven approaches have emerged as powerful tools for establishing correlations between chemically relevant multiparameters that reflect the intrinsic properties of organic molecules. These methods have advanced our understanding of molecular properties and chemical reactivity, laying the groundwork for rational molecular design.^32^ Following data science principles, we applied Principal Component Analysis (PCA) for dimensionality reduction and chemical space visualization, aiming to better capture the complex intrinsic relationships within the data set and identify optimal anolyte candidates. The DFT-based descriptors collected from the oxidized and reduced forms of phthalimides are summarized in Table S2 of the SI and are categorized as follows: electronic parameters, including natural charges, NBO energies, spin density, and HOMO, LUMO, and SOMO energies; and steric parameters, such as Sterimol values and buried volume. Most of these parameters aim to capture correlations with key ROM properties, notably *E*_0_ and stability. After PCA, we performed K-means clustering (*k* = 3, where *k* is the number of clusters specified in the algorithm) on the data set (Figure 4a). The first three principal components (PCs) explain 51.6% of the variance, with the resulting clusters exhibiting distinct property trends. In the following paragraphs, we provide representative examples from the clusters to illustrate additional structure–property trends.

**Figure 4:**
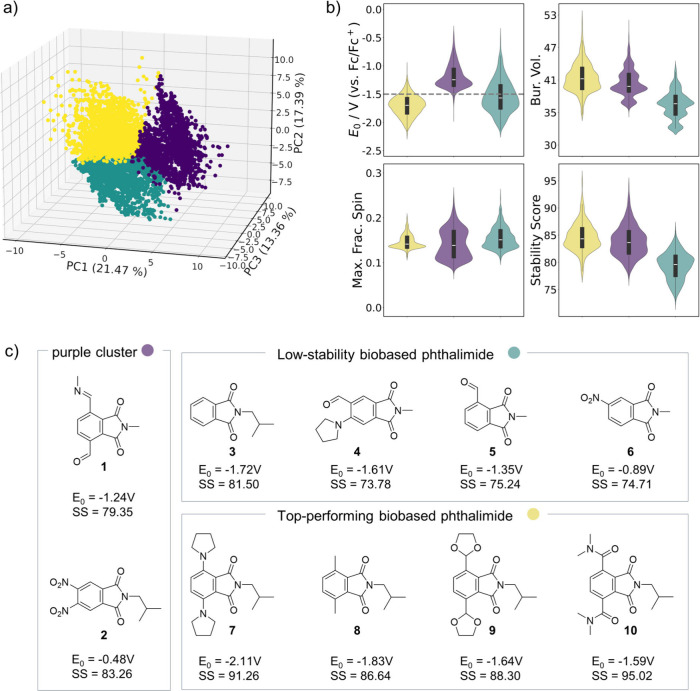
(a) PCA plot of the phthalimide data set, showing three distinct clusters obtained by K-means clustering (*k* = 3). (b) Violin plots comparing key descriptors among clusters: redox potential (*E*_0_), maximum spin density, buried volume at O1 (%*BV*), and stability score (SS), calculated according to eq 3, with the %BV calculated at O1. (c) Selected biobased phthalimide based on their predicted performance.

To further analyze the generated clusters in detail, we used the violin plots shown in Figure 4b. It is evident that the purple cluster exhibits the highest redox potential values, ranging from −1.9 V to −0.39 V, with both the mean and median values of −1.2 V. This indicates that most *E*_0_ are greater than −1.5 V, which is not very interesting for NAORFBs anolytes. The %*BV* values in this cluster are well distributed, ranging from 36% to 51% (mean and median of 41% and 40%, respectively), which indicate that they have relatively high kinetic persistence. The *FSD*_max_ values are also well distributed, ranging from 0.078 to 0.25, with both mean and median of 0.14, indicating that these molecules are, in general, thermodynamically stable. The stability score range is from 77.0 to 94.3, with mean and median values of 83.9 and 83.6, indicating a great part of the molecules in this cluster are stable. While this cluster is not interesting for NAORFBs, its molecules might be useful for AORFBs, since they are both kinetically and thermodynamically stable and their redox potentials are consistent with the electrochemical window of water. Representative examples of biobased phthalimide from this cluster are shown in Figure 4c, which include electron-withdrawing groups such as CHO and CH = NHMe (**1**), and NO_2_ (**2**) incorporated in the aromatic rings, consistent with the more positive redox potentials observed for this group. Their relatively high kinetic persistence can be attributed either to the presence of a bulky R^3^ group, as in compound **2** (R^3^ = *i*-Bu), or to electron-withdrawing substituents at R^1^/R^1^′, which increase the %BV and contribute to radical persistence.

The green cluster presents better redox potentials, compared to the purple cluster, with values ranging from −2.1 V to −0.46 V, with both mean and median of −1.6 V. However, this cluster presents the lowest %*BV*s, ranging from 33% to 44% (both mean and median of 37%), being the poorest regarding kinetic persistence. The *FSD*_max_ values are as distributed as the ones in the purple cluster, ranging from 0.10 to 0.25, and their mean and median values are both 0.15, similar to the purple cluster. The stability scores go from 72.4 to 86.3, with mean and median values of 79.3 and 79.6, and are the poorest among the three clusters. This trend is consistent with the structural patterns observed in this group, which is composed predominantly of molecules bearing small substituents at the R^3^ position (∼70% with R^3^ = Me) and typically monosubstitution at R^1^/R^1^’. A representative example is compound **3** (Figure 4c), where all aromatic positions are unsubstituted, yet the presence of an isobutyl group at R^3^ still results in a SS of 81.5. This suggests that substitution at R^3^ exerts a stronger influence on the stability score than substitution at other positions. Other notable examples include compound **4**, which combines a donor and a withdrawing group at R^2^/R^2^′ and has a small substituent at R^3^ (R^3^ = Me), leading to a moderate redox potential (−1.61 V) but lower stability. Compounds **5** (R^1^ = CHO) and **6** (R^2^ = NO_2_) are monofunctionalized derivatives that display substantial shifts in redox potential along with low radical stability, further illustrating the sensitivity of this cluster to electronic effects.

The yellow cluster has redox potentials ranging from −2.2 to −1.0, with both mean and median values of −1.7 V, being the most suitable for NAORFB anolytes so far. Their buried volumes vary from 36% to 53%, with both mean and median of 42%. and are, therefore, the most kinetically stable compared to the other clusters. Finally, the *FSD*_max_ values range from 0.11 to 0.20, are highly concentrated around its mean and median values of 0.15 and 0.14, respectively, showing great delocalization and, therefore, high thermodynamic stability. The stability score range is from 77.6 to 95.7, with mean and median values of 84.7 and 84.4. These stability scores are the greatest among all clusters. With these analyses, we can conclude that the yellow cluster contains the most suitable phthalimides for RFB anolytes, with more negative redox potentials, high kinetic persistence (great %*BV* values) and high thermodynamic stability (low maximum *FSD*). This high stability performance can be mainly attributed to substitution at the R^1^/R^1^′ positions, regardless of the electronic nature of the substituents, which contributes to increased %*BV* and, consequently, higher SS. Representative compounds **7**–**10** (Figure 4c) illustrate this trend. Among the 802 biobased derivatives found in this cluster (i.e., those with R^3^ = Me or *i*-Bu), most contain electron-donating groups on the aromatic ring contributing to the negative redox potential. For instance, compound **7**, bearing R^1^ = R^1^′ = N­(CH_2_)_5_, shows a very strong donating effect that drives the redox potential to highly negative values (−2.11 V). Methyl groups are also effective in shifting the potential, as seen in compound **8**, which presents a redox potential of −1.83 V. The acetal group in compound **9** is also noteworthy, acting as a weak withdrawing group and resulting in a redox potential of −1.60 V, similar to compound **10**, which contains an amide at R^1^ and exhibits a potential of −1.59 V.

Finally, Figure 5 shows that by only considering the *E*_0_ and the stability score, it is possible to group the molecules more suitable for ORFBs, in which more negative redox potentials and high stability scores are desired. We can also see what was discussed in the cluster analysis from a different point of view: the yellow cluster has more negative redox potentials and high stability scores; the molecules in the purple cluster, although they are very stable, their redox potentials are not negative enough; and finally, the green cluster has a wide range of redox potentials, however, the stability values are the poorest. All computational results for the 5,705 phthalimide derivatives are available in the Supporting Information file phthalimide_data set.xlsx (tab: ‘Computational Data’). This table includes, for each molecule, the canonical SMILES, substitution patterns, reduction potentials, stability, and other descriptors. The column labeled ‘Cluster’ indicates the classification (yellow, green, purple) resulting from the unsupervised chemical space analysis, allowing identification of top-performing candidates. Given the size and complexity of the data set, the data are presented in a structured, machine-readable format to facilitate filtering and selection according to user-defined criteria.

**Figure 5:**
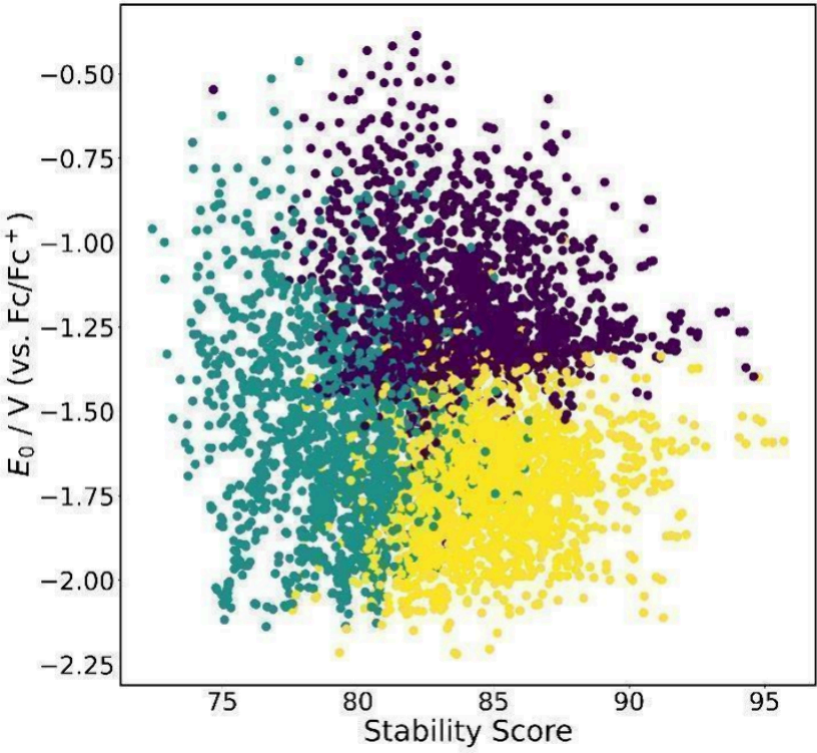
Distribution of redox potential (*E*_0_) versus stability score for all phthalimides data set. The data points are colored according to the K-means clusters previously defined.

#### Structure–Property Relationships of Phthalimides

Establishing clear correlations between molecular structure and physicochemical properties is essential for the rational development of phthalimide-based redox-active materials. In this context, linear free energy relationships (LFERs) and related approaches offer a useful way to explore how structural modifications might influence parameters such as redox potential and radical stability.^32^ While such correlations do not imply causation, they can reveal empirical patterns that help prioritize candidates for further investigation. In particular, identifying consistent structure–property trends within a well-defined chemical space may offer limited predictive value when cautiously applied to structurally related molecules. Our goal in this work was not to define universal rules, but to explore possible descriptors and correlations that could inform early stage design of next-generation anolyte candidates.

To explore computationally inexpensive ways of estimating redox potential across a broader chemical space, we investigated the correlation between *E*_0_ and the singly occupied molecular orbital (SOMO) energy of the reduced species. While frontier orbital energies do not mechanistically define redox behavior, as emphasized in prior works such as Peljo and Girault, they can nonetheless correlate with redox trends under specific conditions.^33^ In our case, SOMO energies were evaluated at two levels of theory (def2-SVP and def2-TZVP), and both showed strong linear correlation with the calculated *E*_0_ values (Figure 6a-b). This supports the potential use of SOMO energy as a rapid, qualitative screening parameter for prioritizing candidates in large-scale computational workflows, where full thermodynamic cycles may be prohibitively expensive. In each case, linear regressions were performed using the complete data set (black dashed line, *R*_*full*_^2^) and the subset of biobased molecules (solid red line, *R*_*bio*_^2^).

**Figure 6:**
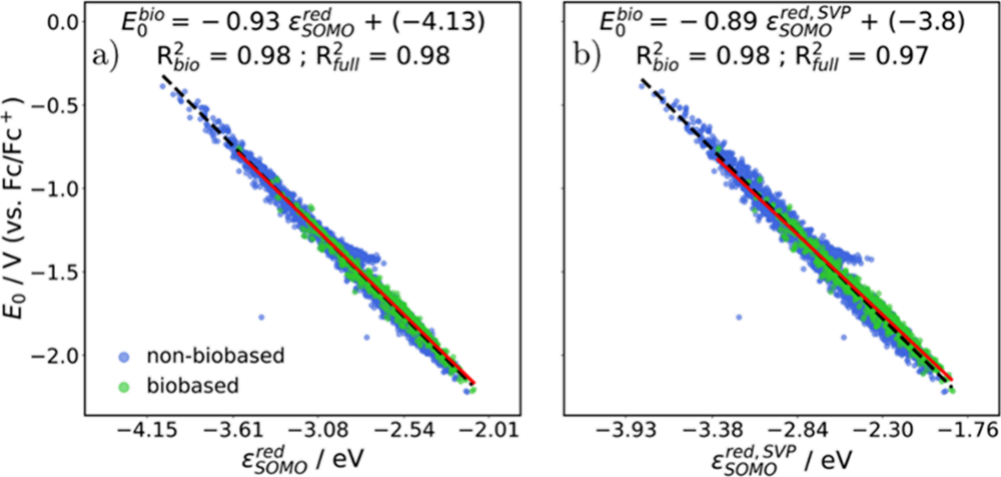
Correlation between the SOMO energy of the reduced species calculated with the def2-TZVP (a) or def2-SVP basis-set (b) and the redox potential *E*_0_. In all cases, *R*_*full*_^2^ values (black dashed lines) refer to the full data set, while *R*_*bio*_^2^ values (solid red lines) refer to the biobased subset.

Another relevant aspect investigated in this study was the predictive capability of the SS in assessing the persistence of the reduced forms of phthalimides. Although the stability SS were not originally derived to predict dimerization directly, they were introduced by Paton and co-workers as general descriptors of radical thermodynamic and kinetic stability, based on benchmarking across well-known radical families.^12^ In subsequent applications, these metrics were successfully applied to describe the persistence of electrochemically generated radicals in the context of ROMs, for which dimerization is one of the major deactivation routes. In the specific case of phthalimide-based redox-active molecules, experimental evidence supports dimerization as a plausible deactivation pathway, reinforcing the relevance of this analysis. (Figure 7a).^22^ As a proxy for radical persistence, we computed the dimerization free energy (ΔG_dim,calc_), which we hypothesized could correlate with the thermodynamic and kinetic stability of these intermediates. For this analysis, only symmetric phthalimides were considered, i.e., those bearing identical substituents at R^1^ = R^1^′ or R^2^ = R^2^′, to simplify the computation of homodimers. To ensure representative sampling, 44 symmetric phthalimide derivatives were selected to span the range of %BV values observed across the data set relevant to dimerization propensity (Figure 7b). It was expected by this strategy that more stable radicals, both thermodynamically and kinetically, would exhibit less negative ΔG_dim,calc_. This hypothesis was confirmed by the correlation observed in Figure 7c (R^2^ = 0.48), supporting the idea that the stability score may serve as a meaningful proxy for dimerization resistance. Building on this insight, a multivariate statistical analysis was performed, which led to the development of a two-term predictive model with substantially improved performance (R^2^ = 0.86; Q^2^ = 0.83; k-fold = 0.82) (Figure 7d). The model also demonstrated moderate external validation ability (R^2^_pred_ = 0.76), based on a 20% hold-out test set. The two descriptors retained in the model reflect both thermodynamic and steric contributions to dimerization: (i) *FSD*, the fractional spin density on C3, previously discussed as a marker of delocalization and thermodynamic stability; and (ii) *sterimol_B5_R1_ox*, which represents the maximum width perpendicular to C7-R1 bond (B5 parameter) of the substituent at the R^1^ position (averaged over R^1^ and R^1^′), thus quantifying the steric bulk that can hinder close approach and consequently dimerization. Although the multivariate model provides enhanced predictive accuracy, its convergence with the original SS reinforces the chemical robustness of this descriptor and its capacity to capture radical persistence and reactivity. In this sense, the multivariate model can be seen as a refined version of the stability score, tailored for the specific case of homodimerization.

**Figure 7:**
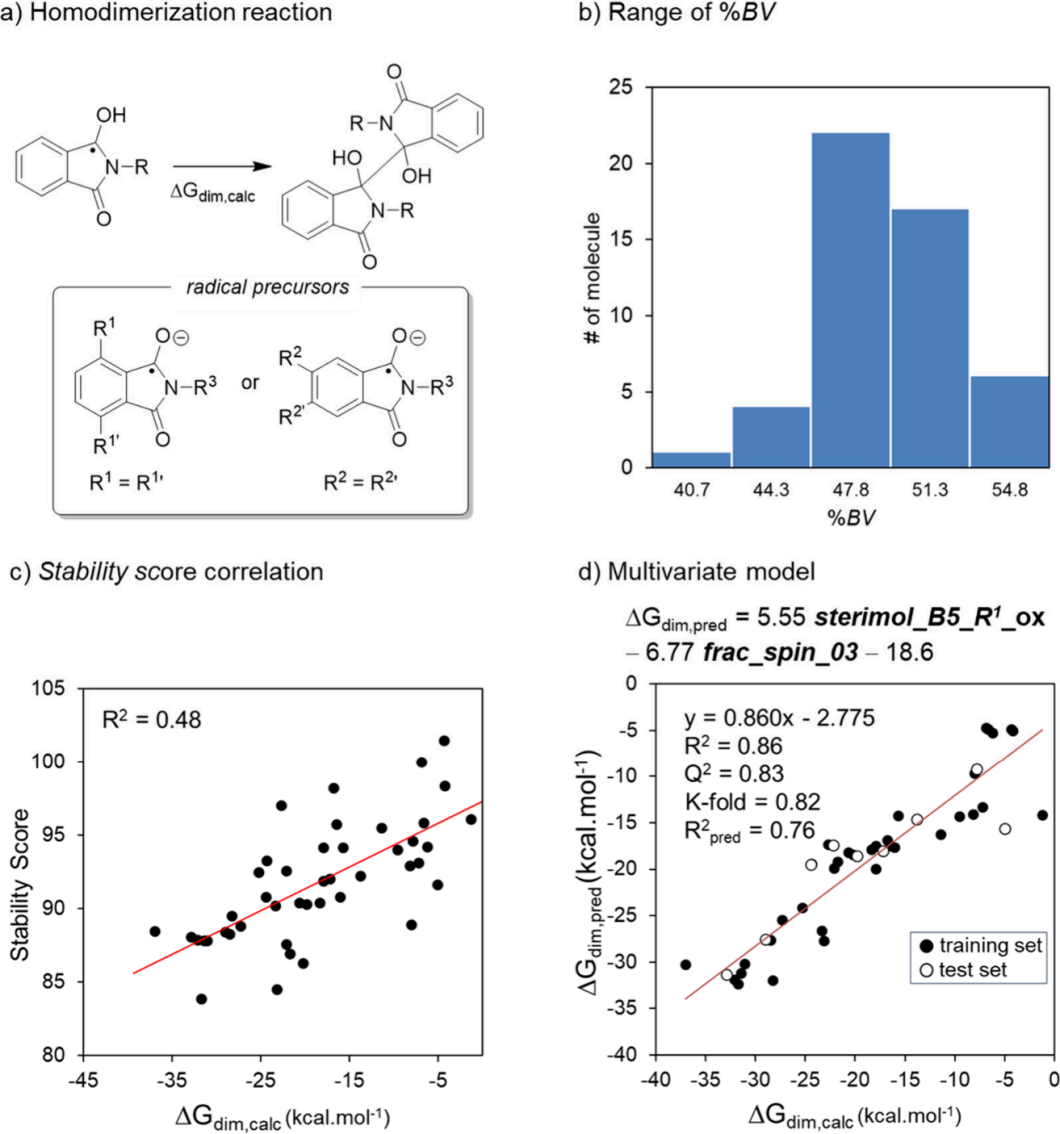
(a) General dimerization mechanism for the protonated radical species of phthalimides, used here as a model of radical persistence. (b) Distribution of selected symmetric phthalimide derivatives based on %*BV*. (c) Correlation between ΔG_dim,calc) and the previously defined stability score (R^2^ = 0.48). (d) Multivariate regression model incorporating two descriptors, *FSD* (fractional spin density at C3) and *sterimol_B5_R1_ox* of oxidized phthalimides.

### 2.3 Synthesis and Electrochemical Characterization of the Phthalimides Candidates

Our next step was to select synthetically accessible candidates to perform preliminary electrochemical experiments, aiming to validate the design strategy proposed in this study. To facilitate visualization of the experimentally investigated compounds within the computed chemical space, a 2D PCA projection with highlighted candidates has been included in the Supporting Information (Figure S11). As a starting point, we focused on the yellow cluster (Figure 8a), which comprises the compounds with the most promising computed physicochemical properties, particularly with respect to redox potential (*E*_0_) and the SS. From the original data set, the selected candidate **8** featured R^1^ = R^1^′ = Me, readily derived from the biobased platform chemicals, and R^3^ = *i*-Bu, a direct derivative of the amino acid valine. For practical reasons related to commercial availability of building blocks, we opted to synthesize phthalimide **8′**, a closely related structural analogue. Phthalimide **8′** retains the same substitution pattern at R^1^ and R^1^′, while R^3^ = isopentyl, a side chain directly derived from leucine. Computational analysis of the key physicochemical descriptors revealed a high degree of similarity between the original candidate **8** and phthalimide **8′** supporting its selection for experimental validation. To further evaluate our computational workflow, we expanded the experimental set to include three additional phthalimide derivatives, intentionally focused on nontop-performing candidates to challenge the predictive robustness of our model. The purple cluster was not selected, as many of its members display SS comparable to those in the yellow (top-performing) cluster as discussed in Figure 4. Instead, we prioritized the green cluster, whose compounds exhibit clearly lower SS offering a more rigorous test of the computational methodology. Among these, compound **3** was expected to have a redox potential comparable to the top-performing derivative but lower SS, due to the absence of Me at R^1^/R^1^′, while bearing an isobutyl group at R^3^. For practical reasons, analog **3′** featuring an isopentyl group at R^3^ was synthesized instead. Finally, compounds **5** and **6** were selected to represent molecules featuring both lower SS and more positive redox potentials.

**Figure 8:**
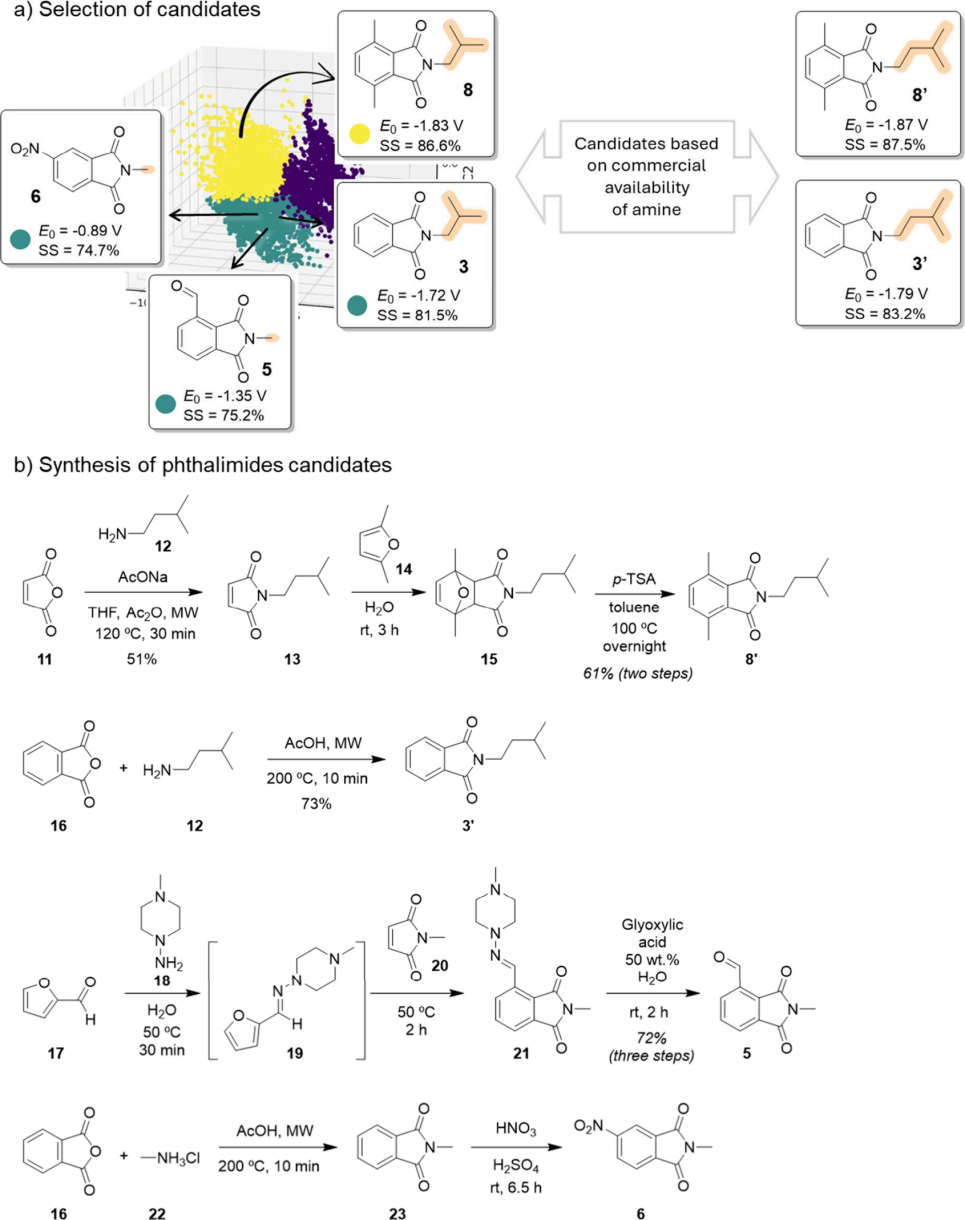
(a) Selected phthalimide candidates with optimal and sup-optimal computed physicochemical properties. (b) Synthetic routes to selected biobased phthalimide candidates.

The synthesis of phthalimide **8′** followed a previous synthetic strategy.^23^ Using furan and maleimide derivatives offers several advantages, particularly due to the possibility of sourcing these starting materials from renewable feedstocks.^34,35^ Grounded by this strategy, the condensation between maleic anhydride (**11**) and isopentylamine (**12**) furnish *N*-isopentylmaleimide (**13**) (Figure 8b). By adopting a Diels–Alder (DA) reaction between **13** and 2,5-dimethylfuran (**14**) in aqueous medium, the corresponding DA adduct (**15**) was obtained in 3 h (Figure 8b). After extraction, the crude product was directly subjected to aromatization using *p*-TSA and phthalimide **8′** was isolated in 61% yield over two steps. The target phthalimide **3′** was synthesized from the condensation reaction of phthalic anhydride (**16**) and **12** in acetic acid under microwave irradiation for 10 min, affording compound **3′** in 73% yield.

The synthesis of phthalimide **5** was more challenging, as the direct DA reaction with furfural is notoriously inefficient due to a HOMO–LUMO mismatch, a well-known limitation in routes to bioaromatics.^23^ To overcome this, an indirect activation strategy was employed by conversion of furfural (**17**) into the corresponding hydrazone derivative **19** generating a more electron-rich diene, enabling a DA-dehydration cascade with maleic anhydride (**20**).^23d^ Subsequent hydrolysis using 50 wt % aqueous glyoxylic acid regenerated the aldehyde functionality, affording **5** in 72% overall yield (two steps). The synthetic route for phthalimide **6** followed a similar strategy of phthalimide **3′**, employing methylamine (**22**) as the amine source. Nitration was then performed via a classical electrophilic aromatic substitution, affording phthalimide **6** in 34% yield.

The electrochemical performance of the synthesized biobased-phthalimide **8′**, **3′**, **5**, and **6** was investigated. Among these, compound **8′** was identified as a top-performing candidate in our computational screening, while the others were selected to experimentally assess lower-performing or intermediate cases, thus validating the predictive robustness of our design strategy. In addition, phthalimide **8′** showed extremely high solubility (>3 mol L^–1^ in acetonitrile), which is desirable to reach RFBs with high power and energy densities. The electrochemical behavior of 10 mmol L^–1^ phthalimide **8′**, **3′**, **5**, and **6** in acetonitrile containing 100 mmol L^–1^ TBAP, were investigated by cyclic voltammograms (CV) on glassy carbon electrode (Figure 9a). The electrochemical behavior of **8′** and **3′** are very similar, with the CV at 50 mV s^–1^ shows a single well-defined redox couple as expected, as phthalimides undergo to reduction/oxidation reactions involving one electron. In contrast, phthalimides **5** and **6** showed multiple redox processes, indicating they can undergo chemical reactions and/or electronic molecular rearrangements following electron transfer, which is not desirable for application in RFBs. These additional peaks likely arise from redox activity of the aromatic substituents (aldehyde and nitro groups), which undergo oxidation or reduction at distinct potentials.

**Figure 9:**
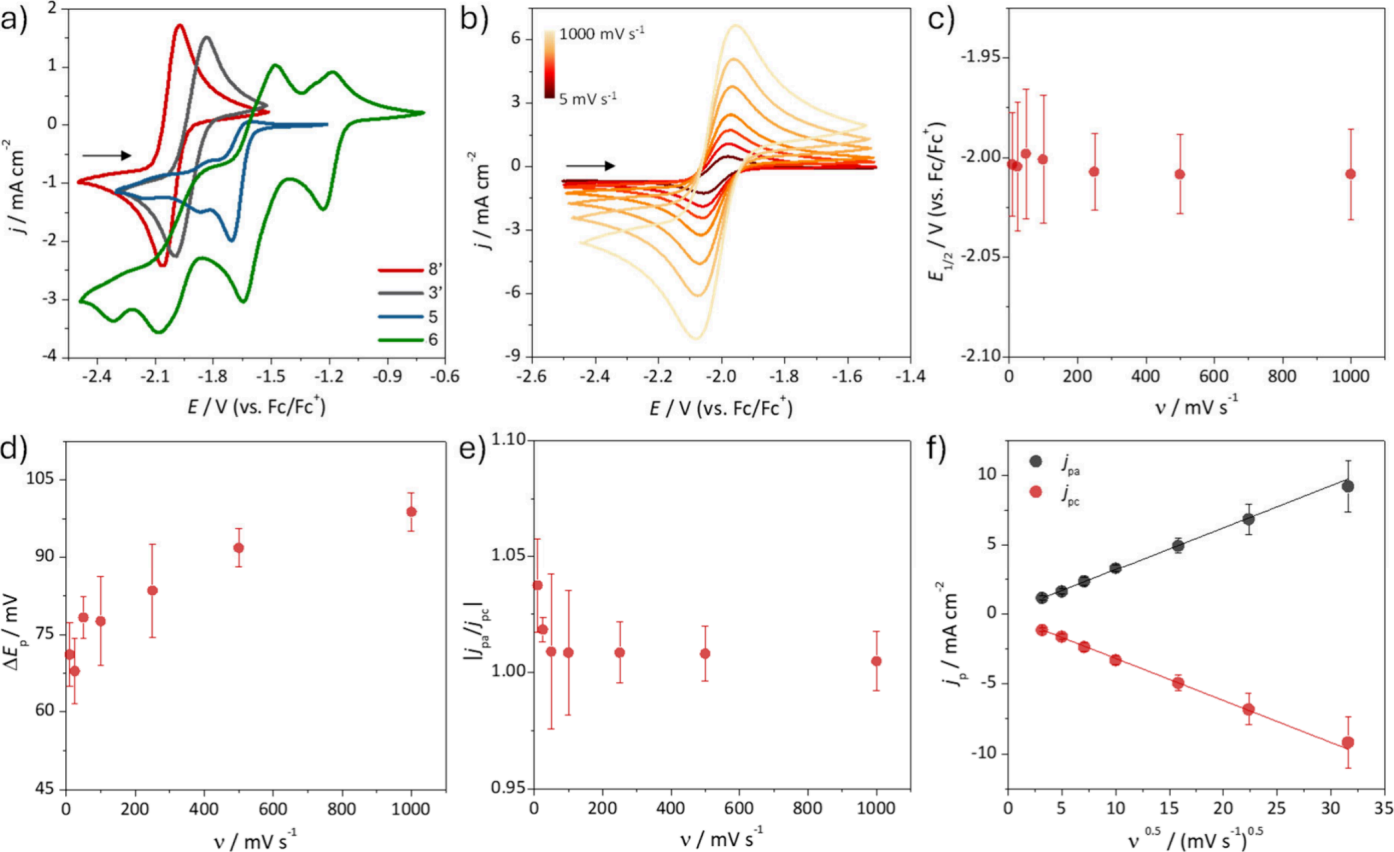
(a) Representative CVs at 50 mV s^–1^ of 10 mmol L^–1^ phthalimide **8′**, **3′**, **5**, and **6**. (b) Cyclic voltammograms of 10 mmol L^–1^ phthalimide **8′** at 10, 25, 50, 100, 250, 500, and 1000 mV s^–1^. Dependence of the average and related standard deviation values of (c) *E*_1/2_, (d) Δ*E*_p_, (e) |*j*_pa_/*j*_pc_|, and (f) *j*_p_ with the scan rate for phthalimide **8′** (based on b). All cyclic voltammogram plots are reported according to (IUPAC convention) and recorded in acetonitrile containing 100 mmol L^–1^ TBAP, as electrolyte, under inert atmosphere, at 25.0 ± 0.1 °C, and using glassy carbon disk, Pt plate, and Ag/Ag^+^ as working, counter, and reference electrodes respectively. Subsequently, all potentials values were converted against Fc/Fc^+^.

For phthalimides **8′** and **3′** the oxidation (*E*_pa_) and reduction (*E*_pc_) peak potentials are equal to −1.959 ± 0.026 V and −2.037 ± 0.027 V, and −1.826 ± 0.071 V and −1.902 ± 0.074 V, respectively, giving an average potential (*E*_1/2_) of −1.998 ± 0.033 V, and −1.864 ± 0.072 V and a peak-to-peak separation (Δ*E*_p_) of 78 ± 4 mV, and 76 ± 23 mV for **8′** and **3′**, respectively. The experimentally determined redox potential of phthalimide **8′** and **3′** closely matches the computed value (*E*_0,calc_ = 1.87 and 1.79 V for **8′** and **3′**, respectively), with deviations of only 6.4% and 6.2%, respectively. The obtained Δ*E*_p_ values slightly larger than 57 mV (expected value for one-electron process and freely diffusing species) indicates quasi-reversible electrochemical reactions of phthalimides **8′** and **3′**. This behavior is confirmed by the electrochemical parameters from CVs at different scan rates (Figure 9b, and Figure S4a in SI). The *E*_1/2_ and Δ*E*_p_ values slightly increased, respectively, from −1.998 to −2.008 V and from 68 to 99 mV for phthalimide **8′** and from −1.884 to −1.860 V and from 74 to 141 mV for phthalimide **3′**, respectively, with the increase of scan rate from 10 to 1000 mV s^–1^ (Figure 9c-d, and Figure S4b-c in SI). In addition, the ratio between the anodic­(*j*_pa_) and cathodic (*j*_pc_) peak currents remained very close to 1.0 in the studied scan rate range (Figure 9e, and Figure S4e in SI), suggesting reduced or oxidized phthalimides **8′** and **3′** are not consumed by subsequent homogeneous chemical reactions.

The *j*_pa_ and *j*_pc_ values reached 2.355 ± 0.258 mA cm^–2^ and −2.340 ± 0.316 mA cm^–2^ for phthalimide **8′** and 2.838 ± 0.919 mA cm^–2^ and −2.823 ± 0.905 mA cm^–2^ for phthalimide **3′** at 50 mV s^–1^ and increased with the scan rate (Figure 9b, and Figure S4b in SI). For both phthalimides, the *j*_pa_ and *j*_pc_ values are controlled by the diffusion of oxidized and reduced species to the electrode surface, as confirmed by the linear relationship of the peak current density values with the square root of the scan rate (Figure 9f, and Figure S 4f in SI), according to Randles–Sevcik equation. Through this equation, the phthalimides **8′** and **3′** diffusion coefficients were calculated in acetonitrile to be 1.25 × 10^–5^ cm^2^ s^–1^ and 1.43 × 10^–5^ cm^2^ s^–1^, respectively, for both oxidized and reduced species (see calculation in the Supporting Information). This high diffusion coefficient compared to other previously reported phthalimide derivatives in acetonitrile^22c^ indicates improved mass transport (>10^–7^ cm^2^ s^–1^), which is crucial for RFB operation.^36^ Also, the electron transfer rate constant (*k* ^0^) values were estimated to be 5.5 × 10^–3^ s^–1^ for phthalimide **8′** and 1.9 × 10^–2^ s^–1^ phthalimide **3′**, according to Nicholson’s analysis and the CV at 50 mV s^–1^ (see calculation in the Supporting Information).^22c,37^ This value agrees with the previously reported values for other phthalimide derivatives.^22c^

To evaluate the electrochemical stability of phthalimide **8′** over successive oxidation/reduction cycles as occur in a RFB, the system was submitted to 2,000 potential cycles at 50 mV s^–1^ (Figure 10a) and the electrochemical parameters were analyzed over the cycles (Figure 10b-d). The redox peak shape remained well-defined over 2,000 potential cycles, with *E*_1/2_ and Δ*E*_p_ values varying by less than 1%. The peak current values and the |*j*_pa_/*j*_pc_| ratio showed less than 10% fade after 2,000 continuous cycles. All those parameters indicate high phthalimide **8′** electrochemical stability toward successive oxidation/reduction cycles. Also, the anolyte was galvanostatically charged/discharged at ±20 mA cm^–2^ and 1000 rpm (Figure 10f) until its total capacity (6.27 C, for 6.5 mL of 10 mmol L^–1^ phthalimide **8′**), and then, the cycled solution was analyzed by spectroscopic techniques. No significant change in the UV–vis (Figures S7 in SI) of the cycled phthalimide **8′** solution was observed, revealing no chemical reactions involving occur. This molecular stability can contribute to further long-term operation of a RFB, with small capacity fading over successive cycles.

**Figure 10:**
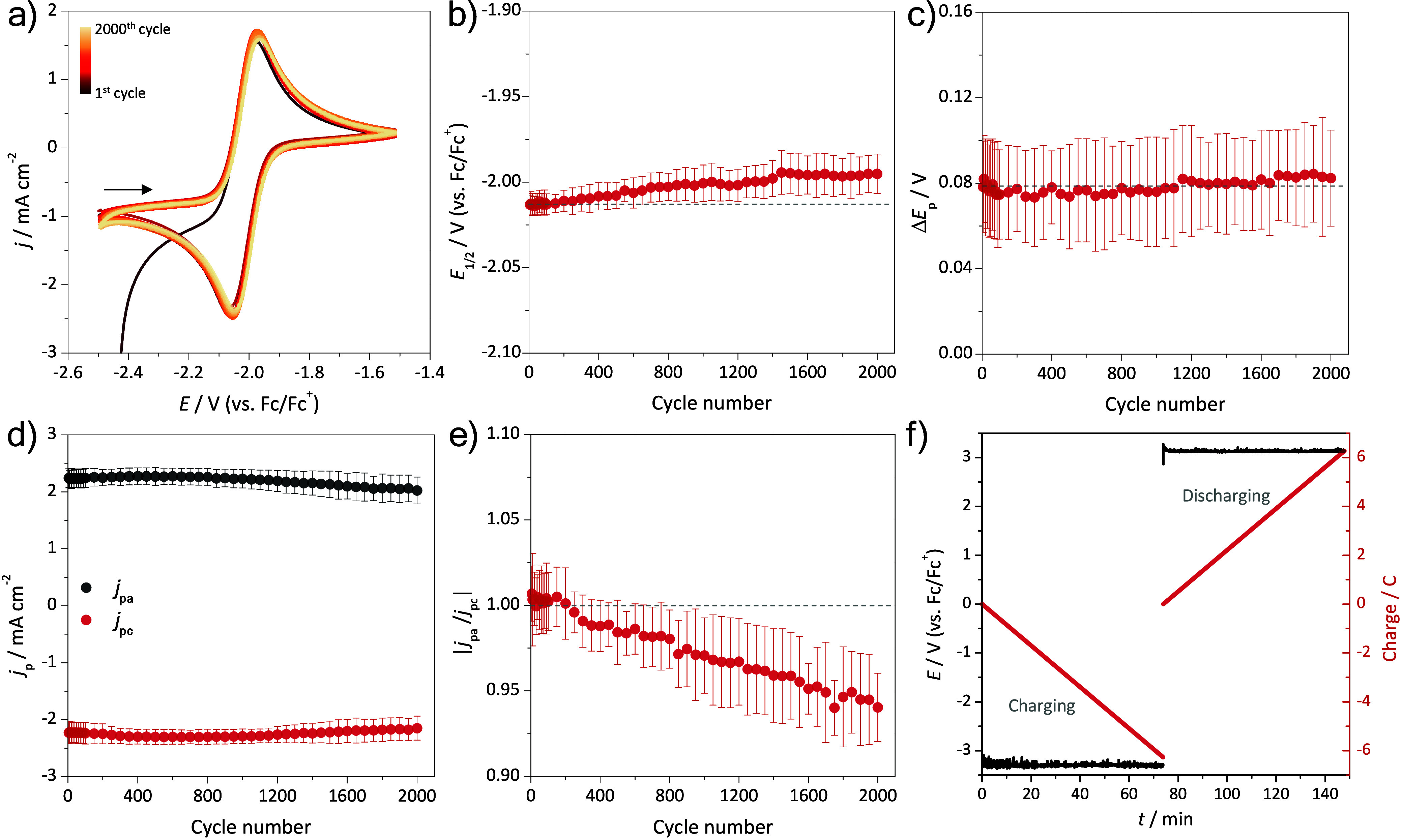
(a) Successive CVs (IUPAC convention) at 50 mV s^–1^ of 10 mmol L^–1^ phthalimide **8′** in acetonitrile containing 100 mmol L^–1^ TBAP. Dependence of the average and related standard deviation values of (b) *E*_1/2_, (c) Δ*E*_p_, (d) *j*_p_, and (e) |*j*_pa_/*j*_pc_| with the scan rate (based on a). Dashed gray lines represent the initial value of each parameter. (f) Galvanostatic charging/discharging cycle at ± 20 mA cm^–2^ and 1000 rpm. All measurements were performed under inert atmosphere, at 25.0 ± 0.1 °C, and using glassy carbon disk, Pt plate, and Ag/Ag^+^ as working, counter, and reference electrodes respectively. Subsequently, all potentials values were converted against Fc/Fc^+^.

Similarly, the electrochemical stability of phthalimide **3′** over successive oxidation/reduction cycles was investigated and the electrochemical parameters were analyzed over the cycles (Figure S5 in SI). The redox peak shape remained well-defined over 2,000 potential cycles, however *E*_1/2_ and Δ*E*_p_ values varied more significantly than those obtained with phthalimide **8′**. The peak current values and the |*j*_pa_/*j*_pc_| ratio showed less than 10% fade after 2,000 continuous cycles. Also, phthalimide **3′**-based anolyte was galvanostatically charged/discharged at ± 20 mA cm^–2^ and 1000 rpm (Figure S8a in SI) until 80% of its total capacity (6.27 C), and then, the cycled solution was analyzed by UV–vis spectroscopy (Figure S8b in SI). Significant change in the UV–vis spectrum of the cycled phthalimide **3′** solution was observed after cycling, revealing chemical reactions can occur during the charge/discharging process. This result combined with the instable voltametric behavior of phthalimide **3′** suggests this compound is unsuitable for application in RFB, as the instability can lead to significant capacity fading over the battery operation. These findings highlight the critical role of substitution at R^1^/R^1^′ in enhancing molecular stability.

Differently to phthalimides **8′** and **3′**, phthalimide **5** showed three quasi-reversible redox processes (*E*_1/2_ values equal to −2.067 ± 0.016 V, −1.814 ± 0.039 V, and −1.640 ± 0.036 V) and phthalimide **6** presented three main oxidation (−1.816 ± 0.027 V, −1.482 ± 0.019 V, and −1.152 ± 0.061 V) and four reduction (−2.320 ± 0.036 V, −2.080 ± 0.053 V, −1.633 ± 0.044 V, and −1.210 ± 0.053 V) processes in the potential range investigated (Figure 9a), indicating irreversible electrochemical behavior for some of single electron transfer steps. The instable voltametric behavior of phthalimides **5** and **6** over 2,000 successive redox cycles (Figure S6, SI), along with their UV–vis spectra before and after galvanostatic charge/discharge at ± 20 mA cm^–2^ up to 80% state-of-charge (Figure S8c-f, SI), provides clear evidence of their chemical and electrochemical degradation, in line with the low SS values predicted computationally and consistent with the known instability of their functional groups.^38^ Therefore, phthalimides **5** and **6** are not promising for application as anolytes in RFBs.

## 3 Conclusions

This study demonstrates the power of high-throughput computational screening phthalimide-based molecules for redox flow batteries. By evaluating critical properties such as redox potential, fractional spin density, and percent buried volume, we identified a subset of highly promising candidates for anolyte applications. These molecules not only meet the requirements of RFBs but also align with sustainability goals through their biobased synthetic routes. Our computational screening of 5,705 phthalimide derivatives highlights key structure–property relationships governing their suitability as RFB anolytes: (i) redox potentials are strongly modulated by substituent electronics, with EDGs driving potentials to more negative values; (ii) spin delocalization is robust across most derivatives, with the phthalimide core dominating the radical character. Exceptions arise with strong EWGs (e.g., NO_2_), which localize spin density; (iii) steric shielding (%*BV*) is critical for kinetic persistence and is well-correlated across key radical sites (O1, C2, C3). The yellow cluster from our K-Means clustering analysis represents the most promising candidates, exhibiting an optimal balance of highly negative *E*_0_, high %*BV*, and delocalized spin density. These findings provide a roadmap for experimental validation and further molecular design, emphasizing EDG-rich phthalimides for more negative redox potentials and sterically hindered substituents to improve radical longevity.

As a proof of concept, we selected four synthetically accessible biobased phthalimide candidates from this optimal region. Particularly, as predicted, phthalimide **8′** exhibited electrochemical properties aligned with RFB anolyte requirements. Its high solubility in acetonitrile, compared to vanadium ions in aqueous RFBs, enables the formulation of high-energy-density electrolytes, while cyclic voltammetry results showed a quasi-reversible redox process with great stability. The diffusion coefficient and electron transfer rate constant highlight the favorable mass transport and kinetic characteristics of the phthalimide derivative. Notably, its electrochemical performance over 2,000 continuous redox cycles and galvanostatic cycling revealed minimal electrochemical and no chemical decompositions, confirming its robust long-term stability. These results position phthalimide **8′** as a strong anolyte candidate for sustainable and high-performance nonaqueous RFB applications.

Finally, the findings presented in this study underscore the potential of computational chemistry to accelerate materials discovery, reducing reliance on time-intensive and costly experimental methods. Future work will focus on synthesizing and experimentally validating the outros top-performing candidates identified in this study, bridging the gap between computational predictions and real-world applications. This approach represents a significant step toward the development of sustainable energy storage solutions, leveraging the power of molecular design and green chemistry principles.

## 4 Experimental Section

All reagents and solvents were purchased from commercial sources (Merck, Sigma-Aldrich) and were used as received except acetonitrile (≥99.9%) and tetra-n-butylammonium perchlorate (TBAP, 99%), which were purchased from Honeywell and Alfa Aesar, respectively. Analytical thin layer chromatography (TLC) was performed using 250 μm silica gel Merck DC Kieselgel 60 (230–400 mesh) precoated plates and performed using UV light. Flash column chromatography was performed using 60 Å, 70–230 mesh Aldrich Co silica gel. Microwave reactions were performed in sealed tubes using a self-tuning CEM Discover focused monomode microwave synthesizer, with temperature monitored in real time by the built-in infrared sensor. Reactions were conducted at the specified temperatures under a fixed power of 70 W. ^1^H and ^13^C NMR spectra were measured in CDCl_3_ using a Bruker Advance 400 (9.4 T) and NMReady Nanalysis (1.4 T) instrument. Chemical shifts are reported relative to tetramethylsilane reported in ppm. High resolution mass spectra (HRMS) were obtained using an Waters Acquity UPLC H-Class coupled with a Waters Xevo G2-XS QToF mass spectrometer equipped with an electrospray ionization (ESI) interface. Infrared spectra were obtained on a Shimadzu FT-IR spectrometer IRSpirit at 4 cm^–1^ resolution and are reported in cm^–1^. UV–vis absorption spectra were recorded using a Shimadzu UV-2600 spectrophotometer in diffuse reflectance mode.

### Synthesis of *N*-Isopentylmaleimide (13):^39^

In a 100 mL round-bottom flask, maleic anhydride **11** (1.5 g, 15 mmol), THF (30 mL), and isopentylamine **12** (650 μL, 5.6 mmol, 0.37 equiv) was added dropwise under stirring at room temperature. After 15 min, acetic anhydride (33 mL) was added, and the solution was divided into three microwave tubes (U_35_). Sodium acetate (0.49 g, 5.9 mmol) was added to each tube, and the mixtures were heated in a microwave reactor at 120 °C for 30 min. Upon cooling, the reaction mixtures turned dark and formed a significant amount of solid. Ethyl acetate (AcOEt) was added (15 mL) to dissolve the organic components. The combined organic phases, from each tube, were washed with saturated aqueous NaHCO_3_ solution (3 × 10 mL). The aqueous layers were back-extracted with additional ethyl acetate (3 × 10 mL). The combined organic extracts were dried over anhydrous Na_2_SO_4_, filtered, and concentrated under reduced pressure. To remove excess acetic anhydride, azeotropic evaporation was carried out by successive dilution of the crude residue in hexane (5 mL) followed by concentration, and then toluene (5 mL), followed again by concentration. The resulting residue was purified by flash column chromatography using a gradient elution of hexane/EtOAc/CH_2_Cl_2_ (8:1:1 → 7:2:1 → 6:3:1), affording a dark brown oil (0.6 g). A second purification step using pure CH_2_Cl_2_ as eluent yielded the target compound **13** in 51% yield as a yellow oil (0.4808 g, 2.86 mmol).^40^ ^1^H NMR (400 MHz, CDCl_3_) δ 6.69 (s, 1H), 3.57–3.49 (m, 1H), 1.55 (dt, *J* = 12.9, 6.5 Hz, 1H), 1.47 (q, *J* = 7.2 Hz, 1H), 0.94 (d, *J* = 6.4 Hz, 6H). ^13^C­{^1^H} NMR (101 MHz, CDCl_3_) δ 170.9, 134.1, 37.2, 36.3, 25.8, 22.3.

### Synthesis of 2-Isopentyl-4,7-dimethylisoindoline-1,3-dione (8′):^34,36^

In a 25 mL round-bottom flask, N-isopentylmaleimide **13** (0.49 g, 3.3 mmol), 2,5-dimethylfuran **14** (3.4 mL, 31.2 mmol, 9.4 equiv), and water (1.9 mL) were combined and stirred at room temperature for 3 h. Upon completion, additional water (5 mL) was added, and the aqueous layer was extracted with ethyl acetate (3 × 5 mL). The combined organic layers were dried over anhydrous Na_2_SO_4_, filtered, and concentrated under reduced pressure to afford the crude Diels–Alder adduct mixture (**15**) as a yellow solid (0.7195 g, 2.73 mmol). Without further purification, the crude product was dissolved in toluene (22 mL), and *p*-toluenesulfonic acid monohydrate (*p*-TSA·H_2_O, 0.26 g, 1.3 mmol, 0.5 equiv) was added. The reaction mixture was refluxed under an argon atmosphere at 100 °C, using an oil bath overnight. After cooling to room temperature, saturated aqueous NaHCO_3_ solution (15 mL) was added, and the aqueous layer was extracted with toluene (3 × 15 mL). The combined organic layers were dried over Na_2_SO_4_, filtered, and concentrated. The crude residue was purified by flash chromatography using a gradient of hexane/ethyl acetate (1:0 → 9:1 → 8:2), yielding the target phthalimide **8′** as a yellow solid (0.4099 g, 1.67 mmol, 61% yield in two steps). IV (KBr, cm^–1^): 2963, 2951, 1693, 1396, 1386. UV–vis: λ_max_ = 315 nm. ^1^H NMR (400 MHz, CDCl_3_) δ 7.29 (s, 2H), 3.77–3.59 (m, 2H), 2.64 (s, 6H), 1.70–1.59 (m, 1H), 1.54 (q, *J* = 7.2 Hz, 2H), 0.97 (d, *J* = 6.4 Hz, 6H). ^13^C­{^1^H} NMR (101 MHz, CDCl_3_) δ 169.3, 135.9, 135.2, 129.0, 37.4, 36.0, 26.0, 22.4, 17.3. (ESI-TOF) *m* **/** *z*: [M + H]^+^ Calcd for C_15_H_20_NO_2_: 246.1489; Found: 246.1487.

### Synthesis of 2-Isopentylisoindoline-1,3-dione (3′):^22a^

In a microwave tube (U_10_) was added phthalic anhydride (**16**) (153 mg, 1 mmol), acetic acid (1.4 mL) and isopentylamine (**12**) (0.09 g, 120 μL, 1 mmol, 1 equiv). The tube was filled with argon and heated for 10 min to 200 °C in the microwave. The solution was poured into saturated sodium carbonate and extracted with DCM/MeCN (5:1 in 10 mL). The organic phase was washed again with sodium carbonate and demineralized water (3 × 10 mL). The combined organic layers were dried over Na2SO4, filtered, and concentrated under reduced pressure. The crude residue was purified by flash chromatography using hexane/ethyl acetate (9:1) yielded the target compound in 73% yield as a colorless oil (164 mg, 0.7 mmol). IV (KBr, cm^–1^): 2959, 2873, 1701, 1394, 704. UV–vis: λ_max_ = 219 nm. ^1^H NMR (400 MHz, CDCl_3_) δ 7.84 (dd, *J* = 5.4, 3.1 Hz, 2H), 7.71 (dd, *J* = 5.5, 3.1 Hz, 2H), 3.70 (t, *J* = 7.3 Hz, 2H), 1.68 – 1.60 (m, 1H), 1.56 (dt, *J* = 8.4, 6.2 Hz, 2H), 0.97 (d, *J* = 6.2 Hz, 6H). ^13^C­{^1^H} NMR (101 MHz, CDCl_3_) δ 168.4, 133.8, 132.2, 123.1, 123.1, 37.3, 36.5, 25.9, 22.4.

### Synthesis of 2-Methyl-1,3-dioxoisoindoline-4-carbaldehyde (5):^23c,41,42^

In a 250 mL round-bottom flask was added furfural (**17**) (1.86 g, 1.6 mL, 19.3 mmol), H_2_O (46 mL) and 1-amino-4-methylpiperazine (**18**) (2.68 g, 2.8 mL, 23.28 mmol, 1.2 equiv), the mixture was heated using an oil bath at 50 °C for 30 min. *N*-methyl maleimide (**20**) (2.15g, 19.5 mmol, 1.01 equiv) was added and the reaction continue with the heat at 50 °C with an oil bath, for 2 h. The flask with the mixture was cooled and the precipitate was collected by filtration under reduced pressure, washed with cold water and dried in the desiccator to afford the crude 2-methyl-4-(((4-methylpiperazin-1-yl)­imino)­methyl)­isoindoline-1,3-dione (**21**) as yellow solid (3.71 g, 12.9 mmol). Without any purification of solid, the crude was transferred to a 100 mL round-bottom flask and was introduced 50% aqueous glyoxylic acid (33.5 g, 25 mL, 0.45 mol, 35 equiv) and the solution was stirred at room temperature for 2 h. Then, additional water (50 mL) was added, and the aqueous layer was extracted with dichloromethane (3 × 50 mL). The combined organic layers were dried over anhydrous Na_2_SO_4_, filtered, and concentrated under reduced pressure. The crude residue was purified by flash chromatography using a gradient of hexane/ethyl acetate (7:3 → 6:4 → 5:5→ 4:6), yielding 2-methyl-1,3-dioxoisoindoline-4-carbaldehyde (**5**) as a pale pink solid (1.78 g, 9.40 mmol, 72% yield in three steps). IV (KBr, cm^–1^): 2950, 2924, 2892, 1687, 1373, 1004, 739. UV–vis: λ_max_ = 227 nm. ^1^H NMR (400 MHz, CDCl_3_) δ 11.03 (s, 1H), 8.25 (d, *J* = 7.8 Hz, 1H), 8.09 (d, *J* = 7.4 Hz, 1H), 7.85 (t, *J* = 7.6 Hz, 1H), 3.24 (s, 3H). ^13^C­{^1^H} NMR (101 MHz, CDCl_3_) δ 188.7, 167.8, 167.3, 134.2, 133.5, 133.0, 132.1, 131.3, 127.9, 29.7, 24.2. HRMS (ESI-TOF) *m* **/** *z*: [M + H]^+^ Calcd for C_10_H_8_NO_3_: 190,0499; Found: 190,0496.

### Synthesis of 2-Methyl-5-nitroisoindoline-1,3-dione (6):^22a,43^

In a microwave tube (U_35_) was added phthalic anhydride (**16**) (2.09 g, 14 mmol), acetic acid (18.5 mL) and methylamine hydrochloride (**22**) (1.05 g, 1 mmol, 1.1 equiv). In the microwave, the mixture was heated for 10 min to 200 °C. Thereafter, the tube was cooled and the precipitate collected by filtration under reduced pressure, washed with cold methanol and dried in the desiccator. The 2-methylisoindoline-1,3-dione (**23**) formed as a white solid (2.14 g) was not purified. Part of this crude (1.12 g, 6.9 mmol) was added in a 25 mL round-bottom flask and dissolved in H_2_SO_4_ (2.7 mL). HNO_3_ (1.38 mL) was poured dropwise over 15 min to this mixture, and the reaction was stirred at room temperature for 6.5 h. The mixture was poured into crushed ice and then filtrated under reduced pressure. The solid was recrystallized with methanol obtaining the 2-methyl-5-nitroisoindoline-1,3-dione (**6**) (481.2 mg, 2.33 mmol, 34% yield) as white needles. IV (KBr, cm^–1^): 1709, 1694, 1527,1063, 1011,718. UV–vis: λ_max_ = 208 nm. ^1^H NMR (400 MHz, CDCl_3_) δ 8.67 (d, *J* = 1.9 Hz, 1H), 8.64–8.58 (m, 1H), 8.06 (d, *J* = 8.1 Hz, 1H), 3.26 (s, 3H). ^13^C­{^1^H} NMR (101 MHz, CDCl_3_) δ 166.3, 166.0, 151.7, 136.6, 133.6, 129.2, 124.4, 118.6, 24.6.

### Electrochemical Measurements

Electrochemical measurements were performed in a potentiostat/galvanostat Metrohm PGSTAT 302N controlled by NOVA 2.1 software and connected to a traditional jacket glass electrochemical cell. A glassy carbon electrode (geometric area = 0.0707 cm^2^), a platinum plate, and an Ag/Ag^+^ electrode (10 mmol L^–1^ AgNO_3_ and 10 mmol L^–1^ TBAP in acetonitrile) were used as working, counter and reference electrodes, respectively. Before each use, glassy carbon electrode was polished subsequently in 0.3 and 0.05 μm alumina slurries, followed by sonication in deionized water for 3 min. The electrochemical cell was cleaned in 25 mmol L^–1^ KMnO_4_ aqueous solution for 12 h, followed by HCl/H_2_O_2_/H_2_O (1/1/5, v/v/v) solution for 30 min, rinsed in deionized water thrice, and finally in boiling deionized water for 30 min. All measurements were performed in a glovebag filled with N_2_ or Ar, and in a solution of 100 mmol L^–1^ tetrabutylammonium perchlorate (TBAP) in acetonitrile containing 10.0 mmol L^–1^ of phthalimides 1, 2, 3, or 4. The electrochemical cell temperature was controlled at 25.0 ± 0.1 °C, by using a thermostatic bath. All potential values were recorded against the Ag/Ag^+^ reference electrode (*E*_Ag/Ag^+^_) and, subsequently, converted against Fc/Fc^+^ (*E*_Fc/Fc^+^_) by the following equation: *E*_Fc/Fc^+^_ = *E*_Ag/Ag^+^_ – 0.114, where all potentials are in volts (V). The measured currents were normalized by the geometric area of the working electrode, for reporting the current density (*j*) values.

### Solubility Test

Solubility of phthalimide **1** was measured in acetonitrile by preparing 100 μL of 0.509 mol L^–1^ in a 1 mL-vial at 25 °C. Then, successive additions of 5 mg were performed, and the solution translucency was observed.

## Supplementary Material











## Data Availability

The data underlying this study are available in the published article and its .

## Notes

Spreadsheet summarizing the electronic energies, Gibbs free energy corrections, and the number of imaginary frequencies (ZIP)
Spreadsheet summarizing the name of the chemical structures and calculated parameters. (ZIP)
Cartesian coordinates of all optimized structures (ZIP)
FAIR data, including the primary NMR FID files, for compounds **8′**, **3′**, **5** and **6** (ZIP)
^1^H NMR and ^13^C NMR spectra of all compounds. Additional electrochemical data. Computational methods. Additional statistical data analysis. (PDF)

## List of Abbreviations:

RFB: Redox Flow Battery
ORFB: Organic Redox Flow Battery
AORFB: Aqueous Organic Redox Flow Battery
NAORFB: Nonaqueous Organic Redox Flow Battery
ROM: Redox-Active Organic Molecule
%BV: Percent Buried Volume
FSD: Fractional Spin Density
NBO: Natural Bond Orbital
DFT: Density Functional Theory
EDG: Electron Donating Group
EWG: Electron Withdrawing Group
PCA: Principal Component Analysis
SS: Stability Score
