# Supplementary material for: Computational Screening of Electroactive Biobased-Phthalimide Molecules for Redox Flow Batteries
Source: J Org Chem. 2026 Jan 6;91(2):842–58. doi: 10.1021/acs.joc.5c01283 (PMC12814552; doi:10.1021/acs.joc.5c01283)
Supplement: Supplementary file 5 [file jo5c01283_si_005.pdf]

Supporting Information

Computational Screening of Electroactive Biobased-Phthalimide Molecules for Redox Flow Batteries

Alex S. Moraes,[†a] Rafaela Binda da Silva,[†a] Murilo A. Dada,[†a] Giovanna Tâmega, Luana Cristina Italiano Faria,[b] Raphaella Von Stein,[a] Graziela C. Sedenho,[b]* Ernesto C. Pereira,[a]* Marco A. B. Ferreira[a]*

[a]*Department of Chemistry, Federal Univeristy of São Carlos (UFSCar), São Carlos, São Paulo CEP 13565-905, Brazil.*
[b]*São Carlos Institute of Chemistry, University of São Paulo (USP), São Carlos, SP 13566-590, Brazil.*

E-mail: grazielasedenho@gmail.com; ernesto@ufscar.br; marco.ferreira@ufscar.br
[†] *These authors contributed equally to this work and share first authorship*

CONTENT

Contents

S1. Functionalization strategies for green substituents....2
S2. Benchmark of Computational Methods for Redox Potential Calculations....6
S3. Statistical Analyses and Calculated Parameters....11
S3.1 Histograms....17
S3.2 Correlations....19
S3.3 Study of stability: radical dimerization....22
S3.1.2 Multivariate Model....25
S4. Electrochemical Characterization of the Phthalimides Candidates 3', 5 and 6....28
S5. Diffusion coefficient and electron transfer constant calculation....31
S6. Electrolyte spectroscopic characterization after galvanostatic cycling....31
S7. Experimental informations of the CEM Discover® focused microwave....34
S8. Two-dimensional PCA projection of the phthalimide chemical space....36
S9. Spectra for synthesized phthalimides and intermediates....37

S1. Functionalization strategies for green substituents

Furan derivatives exhibit broad chemical reactivity, enabling several functional transformations. Starting from furfural, common modifications include oxidation, reduction, reductive amination, acetal formation, and other side-chain functionalizations. Table S1 presents representative transformations used to access the green substituents explored in this study, using biomass-derived furans or aromatics as precursors.

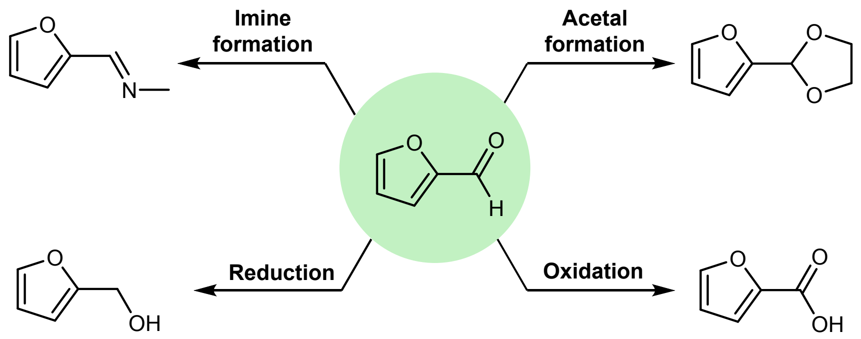

Table S1. List of functionalizations used to obtain biobased substituents.

Reaction type	Reaction	References
Reduction of furfural	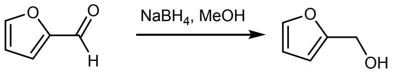	1
	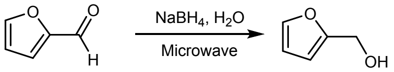	2
Esterification of furfuryl alcohol	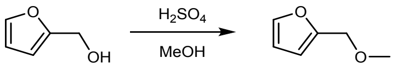	3
	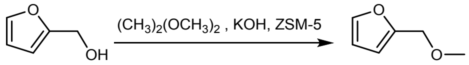	4
	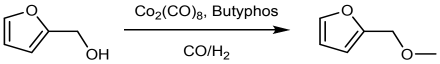	5
Oxidation of furfural	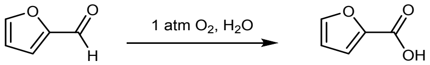	6

[1] Singha, R.; Ray, J. K. Selective Acetylation of Primary Alcohols by Ethyl Acetate. *Tetrahedron Lett.* **2016**, *57* (48), 5395–5398. DOI: 10.1016/j.tetlet.2016.10.088.

[2] Zeynizadeh, B.; Setamdideh, D. Water as a Green Solvent for Fast and Efficient Reduction of Carbonyl Compounds with NaBH4 under Microwave Irradiation. *J. Chin. Chem. Soc.* **2005**, *52* (6), 1179–1184. DOI:10.1002/jccs.200500169.

[3] Mader, S.; REINING, S.; Boehling, R.; Stegmann, V.; Starck, D. Process for Forming Alkyl Ester of Levulinic Acid. US20250002444A1, January 2, **2025**. https://patents.google.com/patent/US20250002444A1/en (accessed 2025-05-15).

[4] Chaffey, D. R.; Davies, T. E.; Taylor, S. H.; Graham, A. E. Etherification Reactions of Furfuryl Alcohol in the Presence of Orthoesters and Ketals: Application to the Synthesis of Furfuryl Ether Biofuels. *ACS Sustainable Chem. Eng.* **2018**, *6* (4), 4996–5002. DOI: 10.1021/acssuschemeng.7b04636.

[5] Delolo, F. G.; Fessler, J.; Neumann, H.; Junge, K.; dos Santos, E. N.; Gusevskaya, E. V.; Beller, M. Cobalt-Catalysed Reductive Etherification Using Phosphine Oxide Promoters under Hydroformylation Conditions. *Chem. Eur. J.* **2022**, *28* (11), e202103903. DOI: 10.1002/chem.202103903.

[6] Zhang, Y.; Cheng, Y.; Cai, H.; He, S.; Shan, Q.; Zhao, H.; Chen, Y.; Wang, B. Catalyst-Free Aerobic Oxidation of Aldehydes into Acids in Water under Mild Conditions. *Green Chem.* **2017**, *19* (23), 5708–5713. DOI: 10.1039/C7GC02983G.

Esterification of furoic acid	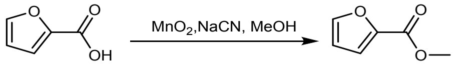	7
	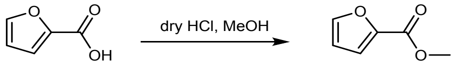	8
Imine formation via condensation	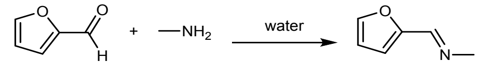	9
	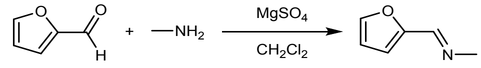	10
	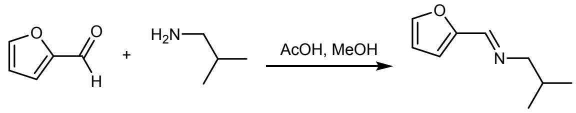	11
	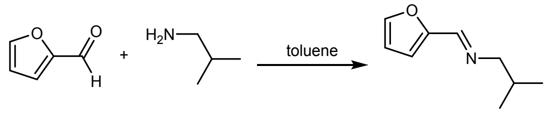	12

[7] Kozlov, K. S.; Romashov, L. V.; Ananikov, V. P. A Tunable Precious Metal-Free System for Selective Oxidative Esterification of Biobased 5-(Hydroxymethyl)Furfural. *Green Chem.* **2019**, *21* (12), 3464–3468. DOI: 10.1039/C9GC00840C.

[8] Cho, J. K.; Lee ,Jae-Soung; Jeong ,Jaewon; Kim ,Bora; Kim ,Baekjin; Kim ,Sangyong; Shin ,Seunghan; Kim ,Hyun-Joong; and Lee, S.-H. Synthesis of Carbohydrate Biomass-Based Furanic Compounds Bearing Epoxide End Group(s) and Evaluation of Their Feasibility as Adhesives. *J. Adhes. Sci. Technol.* **2013**, *27* (18–19), 2127–2138. DOI: 0.1080/01694243.2012.697700.

[9] Nikitina, P. A.; Koldaeva, T. Y.; Zakharko, M. A.; Perevalov, V. P. Synthesis and Study of Prototropic Tautomerism of 2-(2-Furyl)-1-Hydroxyimidazoles. *Aust. J. Chem.* **2020**, *73* (11), 1098–1104. DOI: 10.1071/CH20044.

[10] Song, P.; Li, Q.; Wang, C.; Wu, W.; Mao, X.; Wang, J.; Hu, X. Four-Electron Electrocyclic Ring-Opening/Intermolecular [4+2] Cycloadditions of α-Hydroxycyclobutenones: Stereoselective Synthesis of Multiple Substituted δ-Lactams. *Adv. Syn. Cat.* **2016**, *358* (8), 1208–1212. DOI: 10.1002/adsc.201501152.

[11] Floyd, D. M.; Stein, P.; Wang, Z.; Liu, J.; Castro, S.; Clark, J. A.; Connelly, M.; Zhu, F.; Holbrook, G.; Matheny, A.; Sigal, M. S.; Min, J.; Dhinakaran, R.; Krishnan, S.; Bashyum, S.; Knapp, S.; Guy, R. K. Hit-to-Lead Studies for the Antimalarial Tetrahydroisoquinolone Carboxanilides. *J. Med. Chem.* **2016**, *59* (17), 7950–7962. DOI: 10.1021/acs.jmedchem.6b00752.

[12] Harig, T.; Schlawis, C.; Ziesche, L.; Pohlner, M.; Engelen, B.; Schulz, S. Nitrogen-Containing Volatiles from Marine Salinispora Pacifica and Roseobacter-Group Bacteria. *J. Nat. Prod.* **2017**, *80* (12), 3289–3295. DOI: 10.1021/acs.jnatprod.7b00789.

Reductive amination	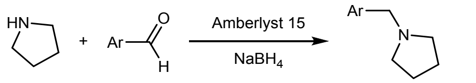	13
	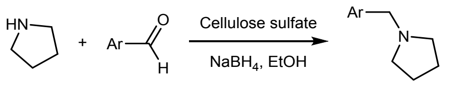	14
Acetal formation	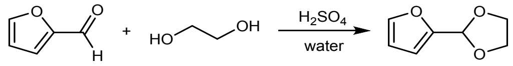	15
Ether Formation	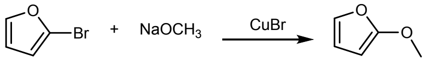	16
Amide formation	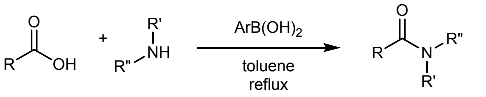	17
Amine formation	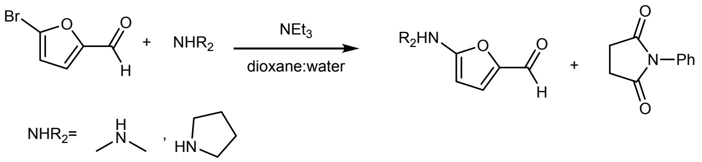	18

---

[13] Alinezhad, H.; Tajbakhsh, M.; Mahdavi, N. One-Pot Reductive Amination of Carbonyl Compounds Using Sodium Borohydride–Amberlyst 15. *Synth. Commun.* **2010**, *40* (7), 951–956. DOI: 10.1080/00397910903026731.

[14] Ranu, B. C.; Majee, A.; Sarkar, A. One-Pot Reductive Amination of Conjugated Aldehydes and Ketones with Silica Gel and Zinc Borohydride. *J. Org. Chem.* **1998**, *63* (2), 370–373. DOI: 10.1021/jo971117h.

[15] РОСТОСТИМУЛИРУЮЩЕЕ СРЕДСТВО ДЛЯ КУЛЬТИВИРОВАНИЯ СУЛЬФАТВОССТАНАВЛИВАЮЩИХ БАКТЕРИЙ. RU2459865C2, August 27, **2012**. https://patenton.ru/patent/RU2459865C2 (accessed 2025-05-15).

[16] Bischof, D.; Zeplichal, M.; Anhäuser, S.; Kumar, A.; Kind, M.; Kramer, F.; Bolte, M.; Ivlev, S. I.; Terfort, A.; Witte, G. Perfluorinated Acenes: Crystalline Phases, Polymorph-Selective Growth, and Optoelectronic Properties. *J. Phys. Chem. C* **2021**, *125* (34), 19000–19012. DOI: 10.1021/acs.jpcc.1c05985.

[17] Ishihara, K.; Ohara, S.; Yamamoto, H. 3,4,5-Trifluorobenzeneboronic Acid as an Extremely Active Amidation Catalyst. *J. Org. Chem.* **1996**, *61* (13), 4196–4197. DOI: 10.1021/jo9606564.

[18] Medimagh, R.; Marque, S.; Prim, D.; Chatti, S.; Zarrouk, H. From Furans to Anilines: Toward One-Pot Two-Step Amination/Diels–Alder Sequences. *J. Org. Chem.* **2008**, *73* (6), 2191–2197. DOI: 10.1021/jo7024916.

S2. Benchmark of Computational Methods for Redox Potential Calculations

To verify the accuracy of different computational methods for the redox potential (E_0) calculation, the redox potential of 30 different molecules with known experimental E_0 values were selected in the literature, and their chemical structures are presented in Figure S1. Molecules 1 to 22 refers to general structures involving aromatic compounds[19], while molecules 23 to 30 are phthalimide-based molecules[20]. All redox potentials were represented against the Fc/Fc^+ reference electrode for comparison with theoretical values.

---

[19] (a) Neugebauer, H.; Bohle, F.; Bursch, M.; Hansen, A.; Grimme, S. Benchmark Study of Electrochemical Redox Potentials Calculated with Semiempirical and DFT Methods. *J. Phys. Chem. A* **2020**, *124* (35), 7166–7176. DOI: 10.1021/acs.jpca.0c05052. (b) Roth, H.; Romero, N.; Nicewicz, D. Experimental and Calculated Electrochemical Potentials of Common Organic Molecules for Applications to Single-Electron Redox Chemistry. *Synlett* **2015**, *27* (05), 714–723. DOI: 10.1055/s-0035-1561297.

[20] (a) Chola, N. M.; Nagarale, R. K. Evaluation and Degradation Mechanism of Phthalimide Derivatives as Anolytes for Non-Aqueous Organic Static Batteries. *New J. Chem.* **2022**, *46* (47), 22593–22601. DOI: 10.1039/D2NJ03495F. (b) Daub, N.; Janssen, R. A. J.; Hendriks, K. H. Imide-Based Multielectron Anolytes as High-Performance Materials in Nonaqueous Redox Flow Batteries. *ACS Appl. Energy Mater.* **2021**, *4* (9), 9248–9257. DOI: 10.1021/acsaem.1c01490.

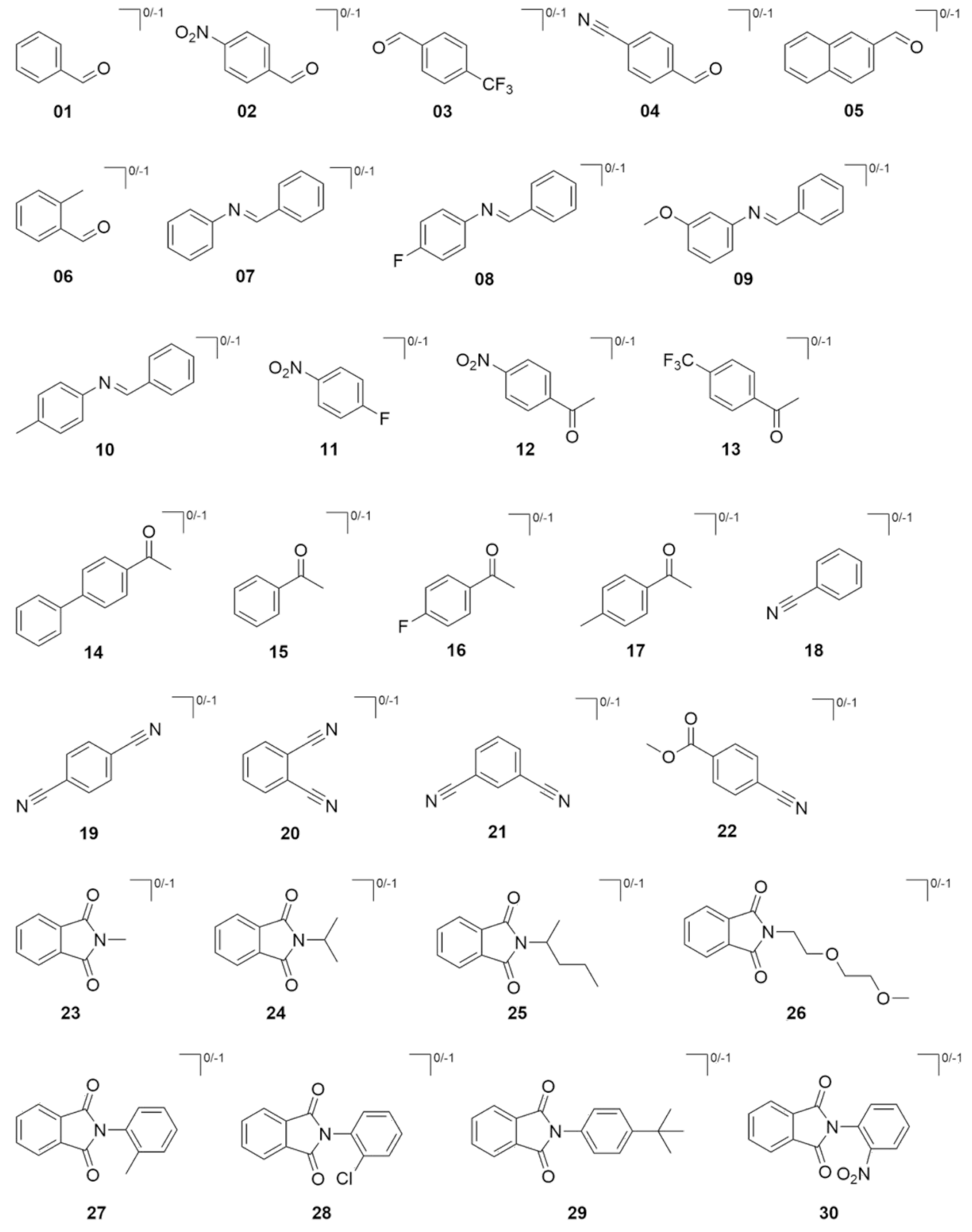

Figure S1

A total of 7 different quantum mechanical levels of theory were compared in this benchmark. The first step was to perform a conformer search to find the lowest energy conformer. After that, the redox potential was calculated according to the following equations

$$E_0 \; = \; -\frac{(\Delta G_{Red} - \Delta G_{Ox})}{nF} - E_0^{Ref} \qquad (1)$$

$$\Delta G = E_{el} + \Delta G_{corr} \qquad (2)$$

where E_0 is the redox potential in Volts (V), ΔG_{Red} and ΔG_{Ox} are the Gibbs free energies of the reduced and oxidized (neutral) species, calculated according to equation (2), E_0^{Ref} is the reference electrode (Fc/Fc^+) redox potential, calculated at the same level of theory, E_{el} is the electronic energy and ΔG_{corr} is the thermal correction to the Gibbs free energy, which includes the zero-point energy (ZPE) and enthalpy and entropy contributions. A similar approach has already been used in the literature[19] to calculate the redox potential.

At the semi-empirical xTB level, both the electronic energy and the thermal corrections were calculated at the GFN2-xTB level. At the *ab-initio* DFT level, two different strategies were used, each for two different exchange-correlation (XC) functionals and for two different sets of basis functions. The first strategy consists in optimizing the geometry with a double-zeta basis-set, def2-SVP, and obtaining the thermal corrections through a vibrational frequency analysis at the same level, while the electronic energy was obtained after a single-point (i.e., no geometry optimization) calculation using a triple-zeta basis-set, def2-TZVP. The two XC functionals used were B97-D3, which is a pure GGA functional that depends on the electronic density and on its gradient; and the M06-2X, which is a meta-GGA functional that depends on the electronic density, its gradient, and its laplacian, making it more accurate, however, more computationally expensive. The second strategy involves obtaining both the thermal correction (after geometry optimization and vibrational frequency analysis) and the electronic energy using the def2-SVP or def2-TZVP basis sets. The latter makes the calculation more time consuming, but improves the accuracy of the results. In summary, the six DFT methods were: M06-2X/def2-TZVP (SP) and B97-D3/def2-TZVP (SP) – with geometry optimizations and thermal corrections using def2-SVP and electronic energies using def2-TZVP, and B97-D3/def2-SVP, M06-2X/def2-SVP, B97-D3/def2-TZVP and M06-2X/def2-TZVP – with both geometry optimizations and electronic energies using def2-SVP or def2-TZVP. The results of these six methods compared to experimental values are shown in Figure S2.

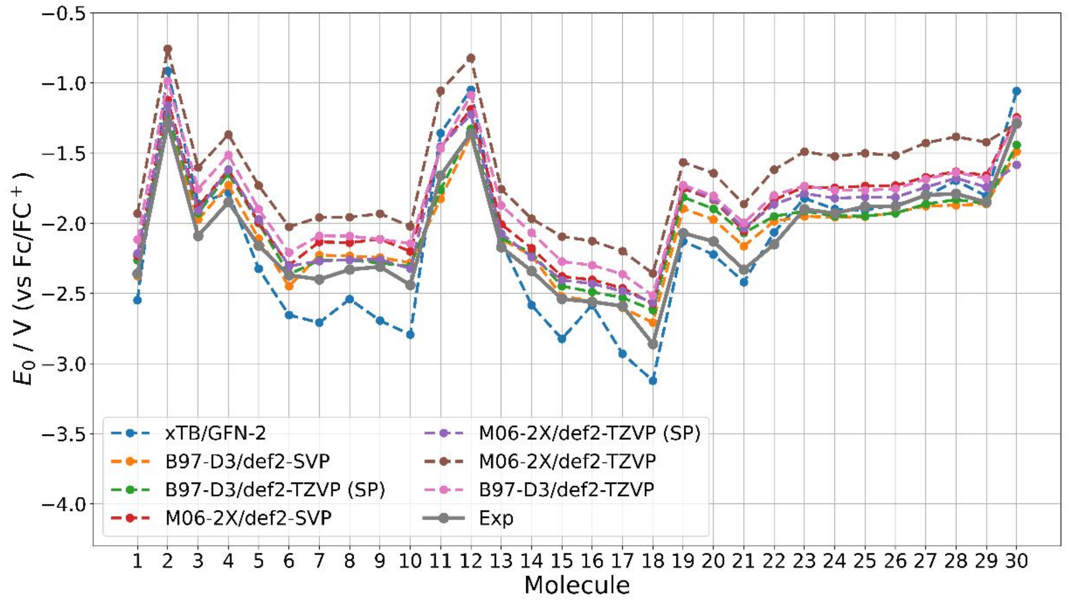

Figure S2

In Figure S2, we can see that all methods follow the general experimental trends, indicating that either one could be used as a pre-screening method based solely on the redox potential. We also analyzed the correlation between each method to the experimental values to obtain a metric (R^2) to indicate the accuracy of the method. These correlations are presented in Figure S3. As we can see, all methods correlate very well with the experimental results, corroborating the results shown in Figure S2.

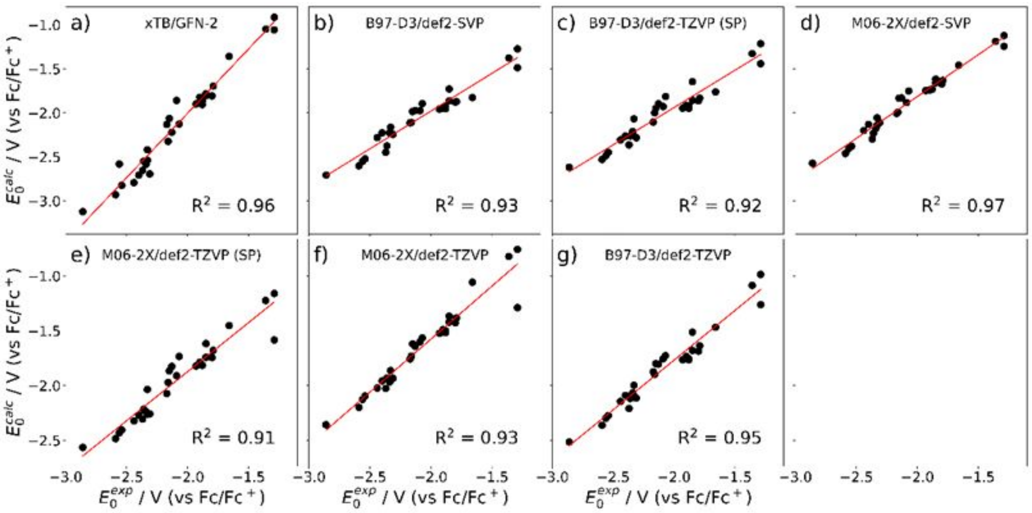

Figure S3

Although xTB and M06-2X/def2-SVP methods have the best R^2 values, they were not chosen for this work due to the following reasons: (i) although xTB is very good for redox potentials, other analyses needed in this work depend on accurate electronic properties, such as fractional atomic spin densities, and since xTB is a semi-empirical method, it cannot provide the accuracy needed for those analyses[21]; and (ii) M06-2X can provide accurate electronic properties, however, the computational cost of using a meta-GGA XC functional can be prohibitive, depending on the size of the dataset. For instance, considering molecule 30, from Figure S1, while the geometry optimization calculation takes 16 minutes using the B97-D3 functional, with the M06-2X it takes 2.2 times longer (35 minutes). Therefore, the method chosen for this work, based on this benchmark, was the B97-D3/def2-TZVP (SP), which has a great R^2 value and a reasonable computational cost.

[21] Cui, Q.; Elstner, M. Density Functional Tight Binding: Values of Semi-Empirical Methods in an Ab Initio Era. *Phys. Chem. Chem. Phys.* **2014**, *16* (28), 14368–14377. DOI: 10.1039/C4CP00908H.

S3. Statistical Analyses and Calculated Parameters

The parameters listed in Table S2 are all the parameters calculated and used for the multidimensional analyses. Individual analyses for each numerical parameter are also presented in the subsequent sections. Within Table S2 is a Figure with a visual representation of the main parameters.

Table S2

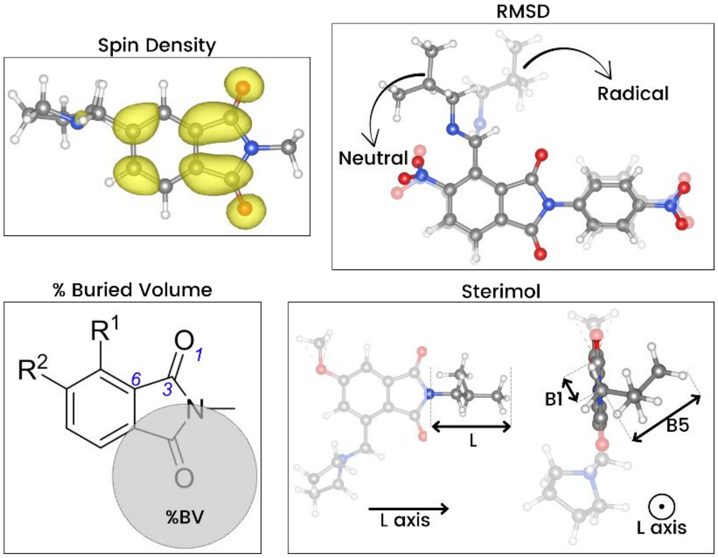

Parameter	Description
R1	R1 functional group
R2	R2 functional group
R3	R3 functional group (attached to nitrogen)
R4 (=R2')	R2' functional group
R5 (=R1')	R1' functional group
E0	Redox potential (V vs. Fc/Fc^{+})
HOMO_ox	HOMO energy of neutral species (eV)
LUMO_ox	LUMO energy of neutral species (eV)
GAP_ox	HOMO-LUMO gap of neutral species (eV)

SOMO_red	SOMO energy of reduced species (eV)
LUMO_red	LUMO energy of reduced species (eV)
ox_electric_dipole_moment	Electric dipole moment of neutral species (D)
red_electric_dipole_moment	Electric dipole moment of reduced species (D)
solvation_energies	Solvation energies of biobased phthalimides (kcal/mol)
frac_spin_01	Fractional spin density of atom 01
frac_spin_03	Fractional spin density of atom 03
frac_spin_04	Fractional spin density of atom 04
frac_spin_06	Fractional spin density of atom 06
frac_spin_07	Fractional spin density of atom 07
frac_spin_08	Fractional spin density of atom 08
frac_spin_12	Fractional spin density of atom 12
frac_spin_13	Fractional spin density of atom 13
frac_spin_14	Fractional spin density of atom 14
greatest_frac_spin_atom	Atom with the greatest fractional spin density
greatest_frac_spin_value	Greatest fractional spin density value
ch_nbo_ox_01	NBO charge on atom 01 of neutral species
ch_nbo_ox_03	NBO charge on atom 03 of neutral species
ch_nbo_ox_04	NBO charge on atom 04 of neutral species
ch_nbo_ox_06	NBO charge on atom 06 of neutral species
ch_nbo_ox_07	NBO charge on atom 07 of neutral species
ch_nbo_ox_08	NBO charge on atom 08 of neutral species

ch_nbo_ox_12	NBO charge on atom 12 of neutral species
ch_nbo_ox_13	NBO charge on atom 13 of neutral species
ch_nbo_ox_14	NBO charge on atom 14 of neutral species
ch_nbo_red_01	NBO charge on atom 01 of reduced species
ch_nbo_red_03	NBO charge on atom 03 of reduced species
ch_nbo_red_04	NBO charge on atom 04 of reduced species
ch_nbo_red_06	NBO charge on atom 06 of reduced species
ch_nbo_red_07	NBO charge on atom 07 of reduced species
ch_nbo_red_08	NBO charge on atom 08 of reduced species
ch_nbo_red_12	NBO charge on atom 12 of reduced species
ch_nbo_red_13	NBO charge on atom 13 of reduced species
ch_nbo_red_14	NBO charge on atom 14 of reduced species
LP1_01_occ	Occupancy of first lone pair on atom 01
LP2_01_occ	Occupancy of second lone pair on atom 01
BD1_01-03_occ	Occupancy of first bonding pair between atoms 01 and 03
BD1nl_01-03_occ	Occupancy of first nonbonding pair between atoms 01 and 03
BD2nl_01-03_occ	Occupancy of second nonbonding pair between atoms 01 and 03
BD2_01-03_occ	Occupancy of second bonding pair between atoms 01 and 03
LP1_01_coef	Coefficient of first lone pair on atom 01
LP2_01_coef	Coefficient of second lone pair on atom 01

BD1_01-03_coef	Coefficient of first bonding pair between atoms 01 and 03
BD1nl_01-03_coef	Coefficient of first nonbonding pair between atoms 01 and 03
BD2_01-03_coef	Coefficient of second bonding pair between atoms 01 and 03
BD2nl_01-03_coef	Coefficient of second nonbonding pair between atoms 01 and 03
sterimol_L_R1_ox	Sterimol L parameter on R1 of neutral species
sterimol_B1_R1_ox	Sterimol B1 parameter on R1 of neutral species
sterimol_B5_R1_ox	Sterimol B5 parameter on R1 of neutral species
sterimol_L_R2_ox	Sterimol L parameter on R2 of neutral species
sterimol_B1_R2_ox	Sterimol B1 parameter on R2 of neutral species
sterimol_B5_R2_ox	Sterimol B5 parameter on R2 of neutral species
sterimol_L_R3_ox	Sterimol L parameter on R3 of neutral species
sterimol_B1_R3_ox	Sterimol B1 parameter on R3 of neutral species
sterimol_B5_R3_ox	Sterimol B5 parameter on R3 of neutral species
sterimol_L_R1_red	Sterimol L parameter on R1 of reduced species
sterimol_B1_R1_red	Sterimol B1 parameter on R1 of reduced species
sterimol_B5_R1_red	Sterimol B5 parameter on R1 of reduced species
sterimol_L_R2_red	Sterimol L parameter on R2 of reduced species
sterimol_B1_R2_red	Sterimol B1 parameter on R2 of reduced species
sterimol_B5_R2_red	Sterimol B5 parameter on R2 of reduced species
sterimol_L_R3_red	Sterimol L parameter on R3 of reduced species

sterimol_B1_R3_red	Sterimol B1 parameter on R3 of reduced species
sterimol_B5_R3_red	Sterimol B5 parameter on R3 of reduced species
bv_ox_01	Percent buried volume on atom 01 of neutral species
bv_ox_03	Percent buried volume on atom 03 of neutral species
bv_ox_04	Percent buried volume on atom 04 of neutral species
bv_ox_06	Percent buried volume on atom 06 of neutral species
bv_ox_07	Percent buried volume on atom 07 of neutral species
bv_ox_08	Percent buried volume on atom 08 of neutral species
bv_ox_12	Percent buried volume on atom 12 of neutral species
bv_ox_13	Percent buried volume on atom 13 of neutral species
bv_ox_14	Percent buried volume on atom 14 of neutral species
bv_red_01	Percent buried volume on atom 01 of reduced species
bv_red_03	Percent buried volume on atom 03 of reduced species
bv_red_04	Percent buried volume on atom 04 of reduced species
bv_red_06	Percent buried volume on atom 06 of reduced species
bv_red_07	Percent buried volume on atom 07 of reduced species
bv_red_08	Percent buried volume on atom 08 of reduced species
bv_red_12	Percent buried volume on atom 12 of reduced species
bv_red_13	Percent buried volume on atom 13 of reduced species
bv_red_14	Percent buried volume on atom 14 of reduced species
mean_bv_ox	Mean percent buried volume of neutral species
mean_bv_red	Mean percent buried volume of reduced species

abs_bv_difference	Absolute difference between neutral and reduced mean percent buried volumes
sascore	Synthetic Accessibility Score
rmsd	RMSD between neutral and reduced species

S3.1 Histograms

Below, the distribution of all numerical parameters is shown. The absolute frequency refers to the total number of occurrences (molecules) for each property value.

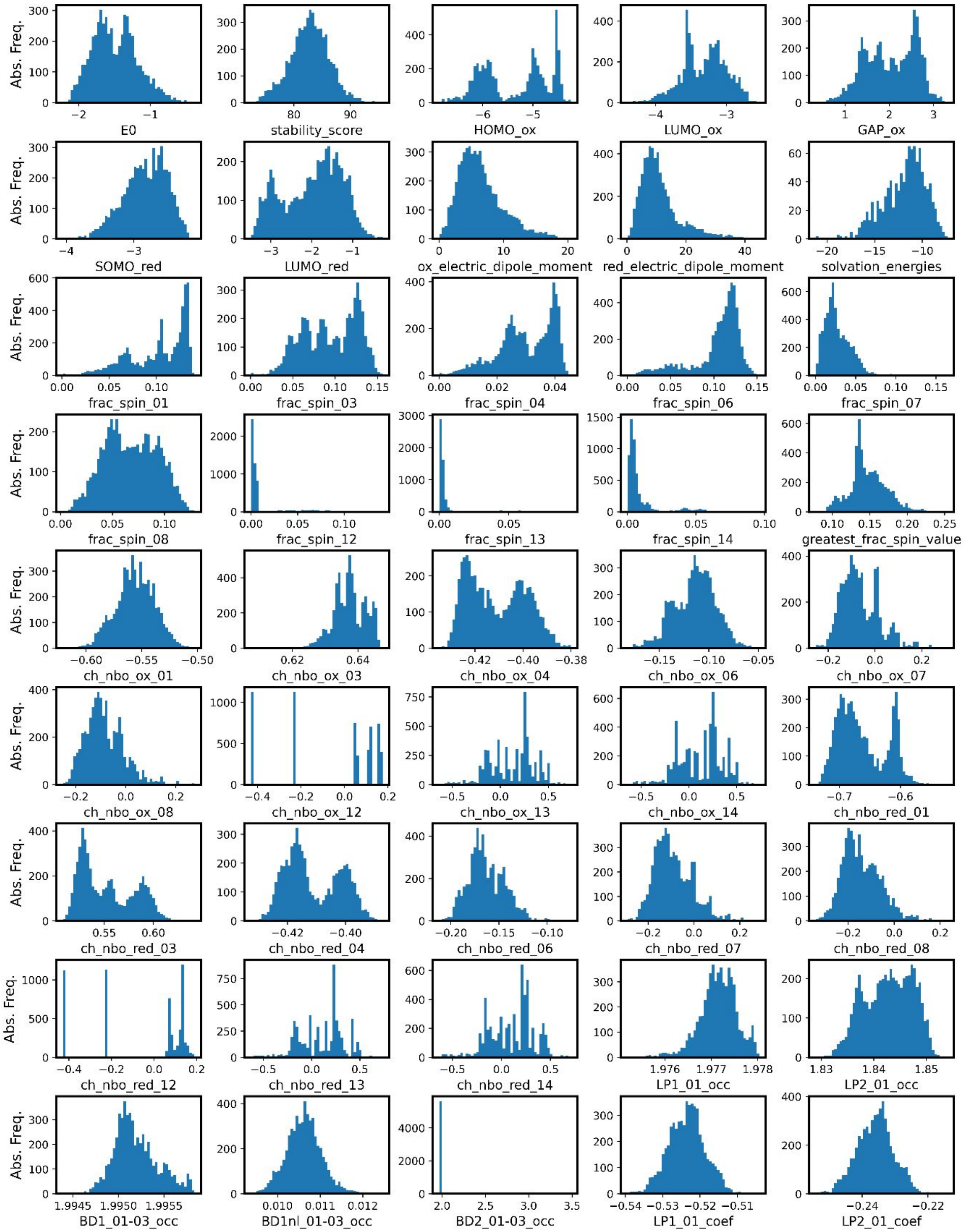

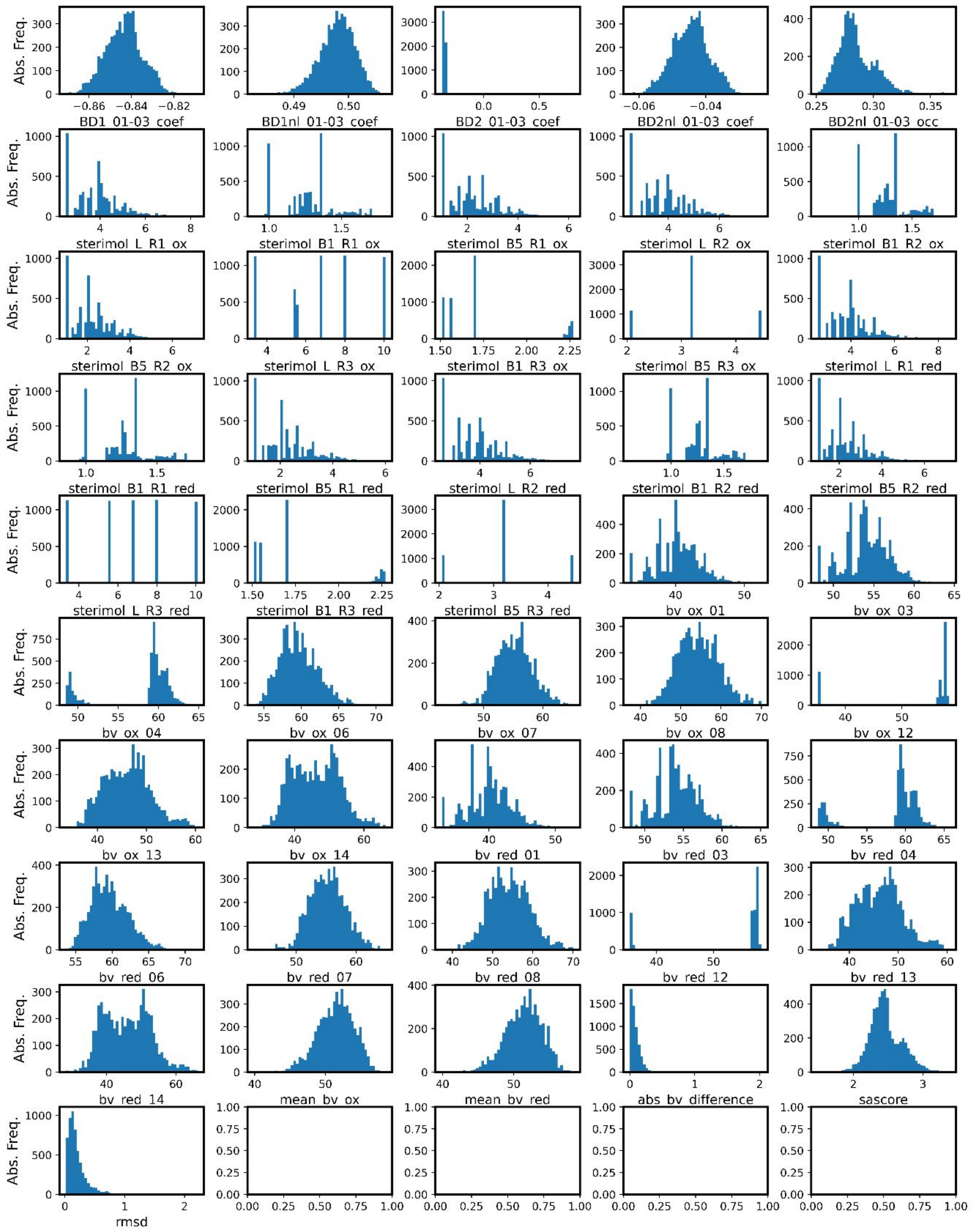

S3.2 Correlations

For all numerical properties, their linear correlation with each other was calculated and every pair of properties with $R^2 > 0.8$ are presented below.

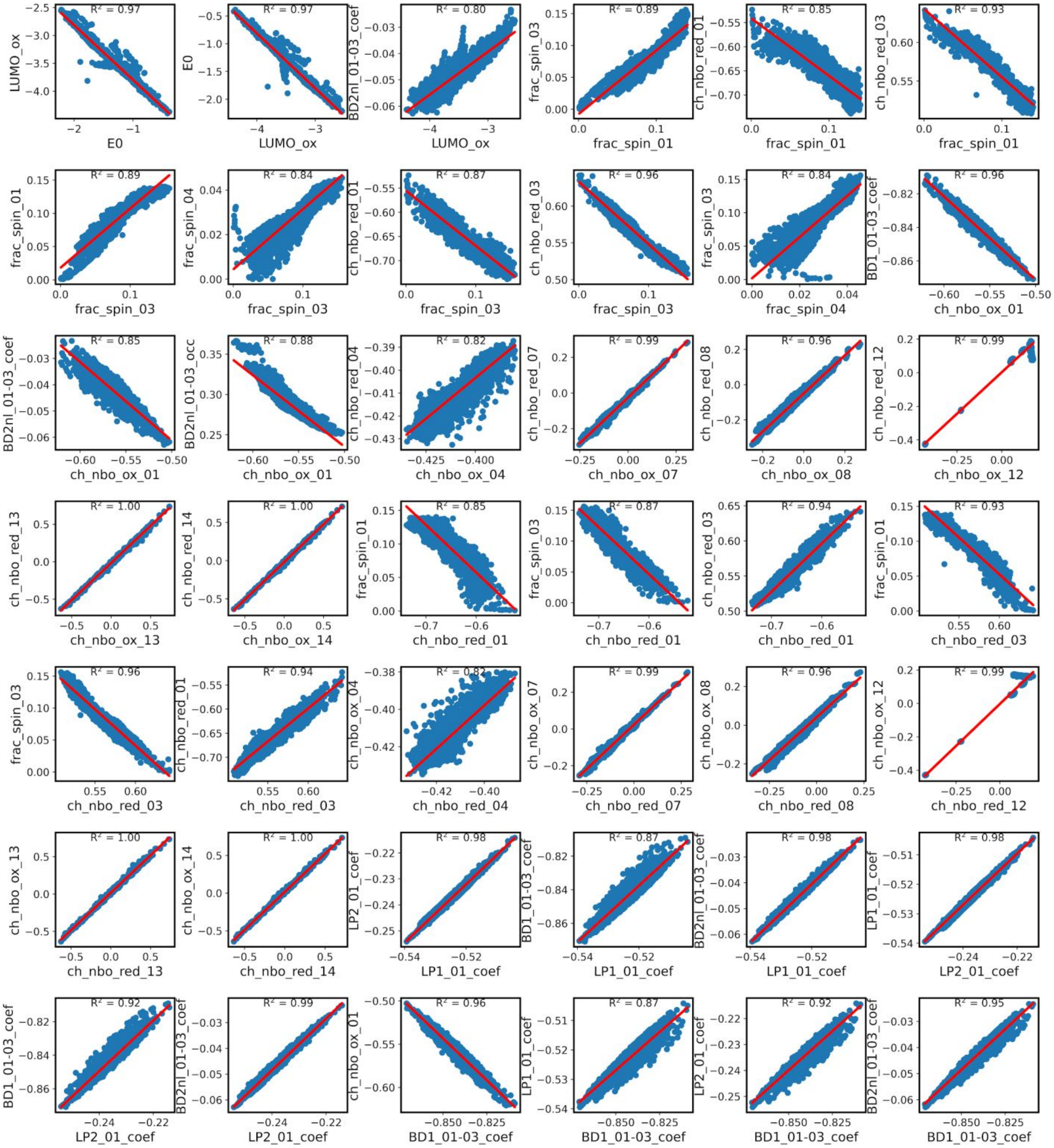

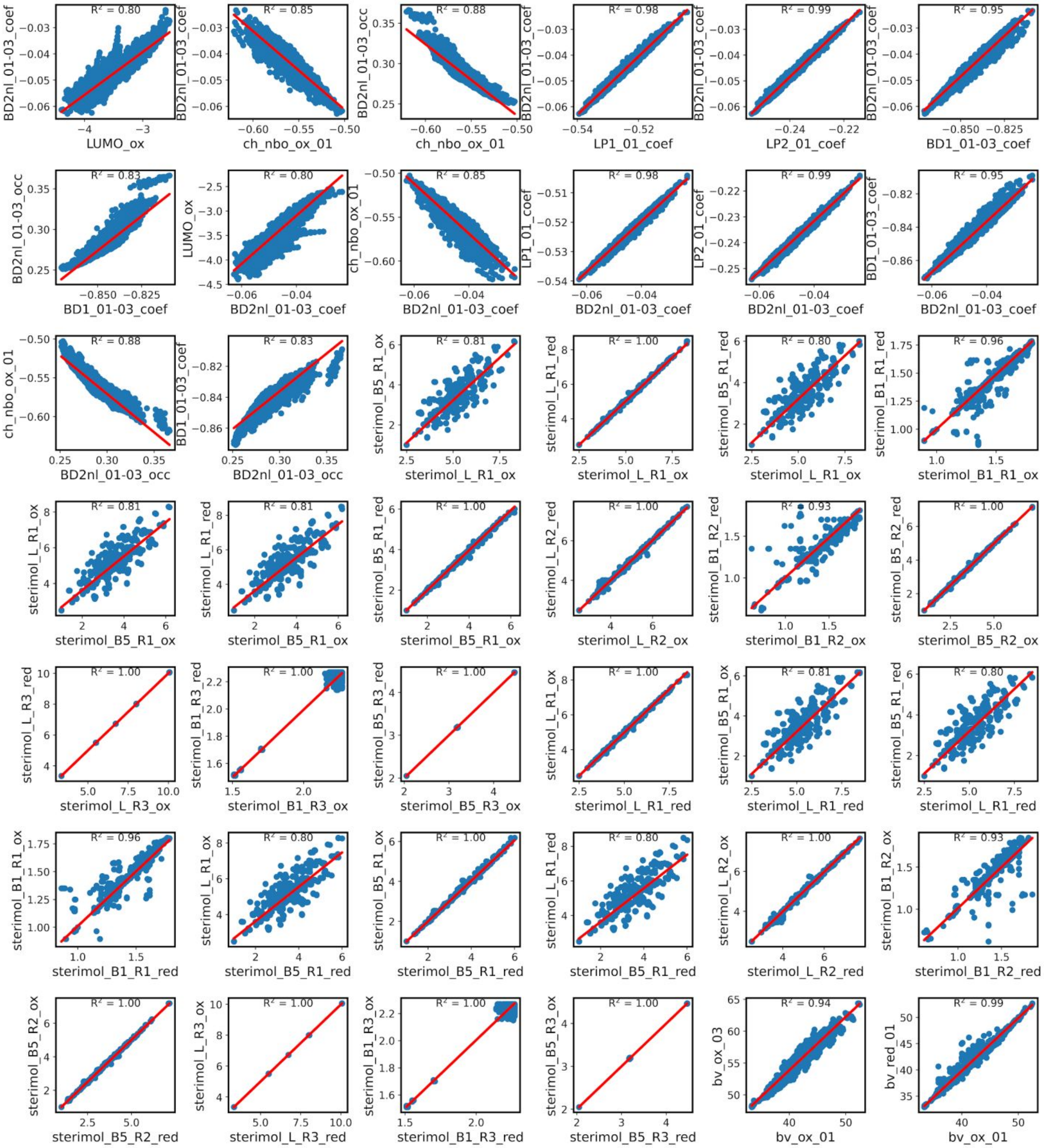
R^2 = 0.80
BD2nl_01-03_coef
LUMO_ox
R^2 = 0.85
BD2nl_01-03_coef
ch_nbo_ox_01
R^2 = 0.88
BD2nl_01-03_occ
ch_nbo_ox_01
R^2 = 0.98
BD2nl_01-03_coef
LP1_01_coef
R^2 = 0.99
BD2nl_01-03_coef
LP2_01_coef
R^2 = 0.95
BD2nl_01-03_coef
BD1_01-03_coef
R^2 = 0.83
BD2nl_01-03_occ
BD1_01-03_coef
R^2 = 0.80
LUMO_ox
BD2nl_01-03_coef
R^2 = 0.85
ch_nbo_ox_01
BD2nl_01-03_coef
R^2 = 0.98
LP1_01_coef
BD2nl_01-03_coef
R^2 = 0.99
LP2_01_coef
BD2nl_01-03_coef
R^2 = 0.95
BD1_01-03_coef
BD2nl_01-03_coef
R^2 = 0.88
ch_nbo_ox_01
BD2nl_01-03_occ
R^2 = 0.83
BD1_01-03_coef
BD2nl_01-03_occ
R^2 = 0.81
sterimol_B5_R1_ox
sterimol_L_R1_ox
R^2 = 1.00
sterimol_L_R1_red
sterimol_L_R1_ox
R^2 = 0.80
sterimol_B5_R1_red
sterimol_L_R1_ox
R^2 = 0.96
sterimol_B1_R1_red
sterimol_B1_R1_ox
R^2 = 0.81
sterimol_L_R1_ox
sterimol_B5_R1_ox
R^2 = 0.81
sterimol_L_R1_red
sterimol_B5_R1_ox
R^2 = 1.00
sterimol_B5_R1_red
sterimol_B5_R1_ox
R^2 = 1.00
sterimol_L_R2_red
sterimol_L_R2_ox
R^2 = 0.93
sterimol_B1_R2_red
sterimol_B1_R2_ox
R^2 = 1.00
sterimol_B5_R2_red
sterimol_B5_R2_ox
R^2 = 1.00
sterimol_L_R3_red
sterimol_L_R3_ox
R^2 = 1.00
sterimol_B1_R3_red
sterimol_B1_R3_ox
R^2 = 1.00
sterimol_B5_R3_red
sterimol_B5_R3_ox
R^2 = 1.00
sterimol_L_R1_ox
sterimol_L_R1_red
R^2 = 0.81
sterimol_B5_R1_ox
sterimol_L_R1_red
R^2 = 0.80
sterimol_B5_R1_red
sterimol_L_R1_red
R^2 = 0.96
sterimol_B1_R1_ox
sterimol_B1_R1_red
R^2 = 0.80
sterimol_L_R1_ox
sterimol_B5_R1_red
R^2 = 1.00
sterimol_B5_R1_ox
sterimol_B5_R1_red
R^2 = 0.80
sterimol_L_R1_red
sterimol_B5_R1_red
R^2 = 1.00
sterimol_L_R2_ox
sterimol_L_R2_red
R^2 = 0.93
sterimol_B1_R2_ox
sterimol_B1_R2_red
R^2 = 1.00
sterimol_B5_R2_ox
sterimol_B5_R2_red
R^2 = 1.00
sterimol_L_R3_ox
sterimol_L_R3_red
R^2 = 1.00
sterimol_B1_R3_ox
sterimol_B1_R3_red
R^2 = 1.00
sterimol_B5_R3_ox
sterimol_B5_R3_red
R^2 = 0.94
bv_ox_03
bv_ox_01
R^2 = 0.99
bv_red_01
bv_ox_01

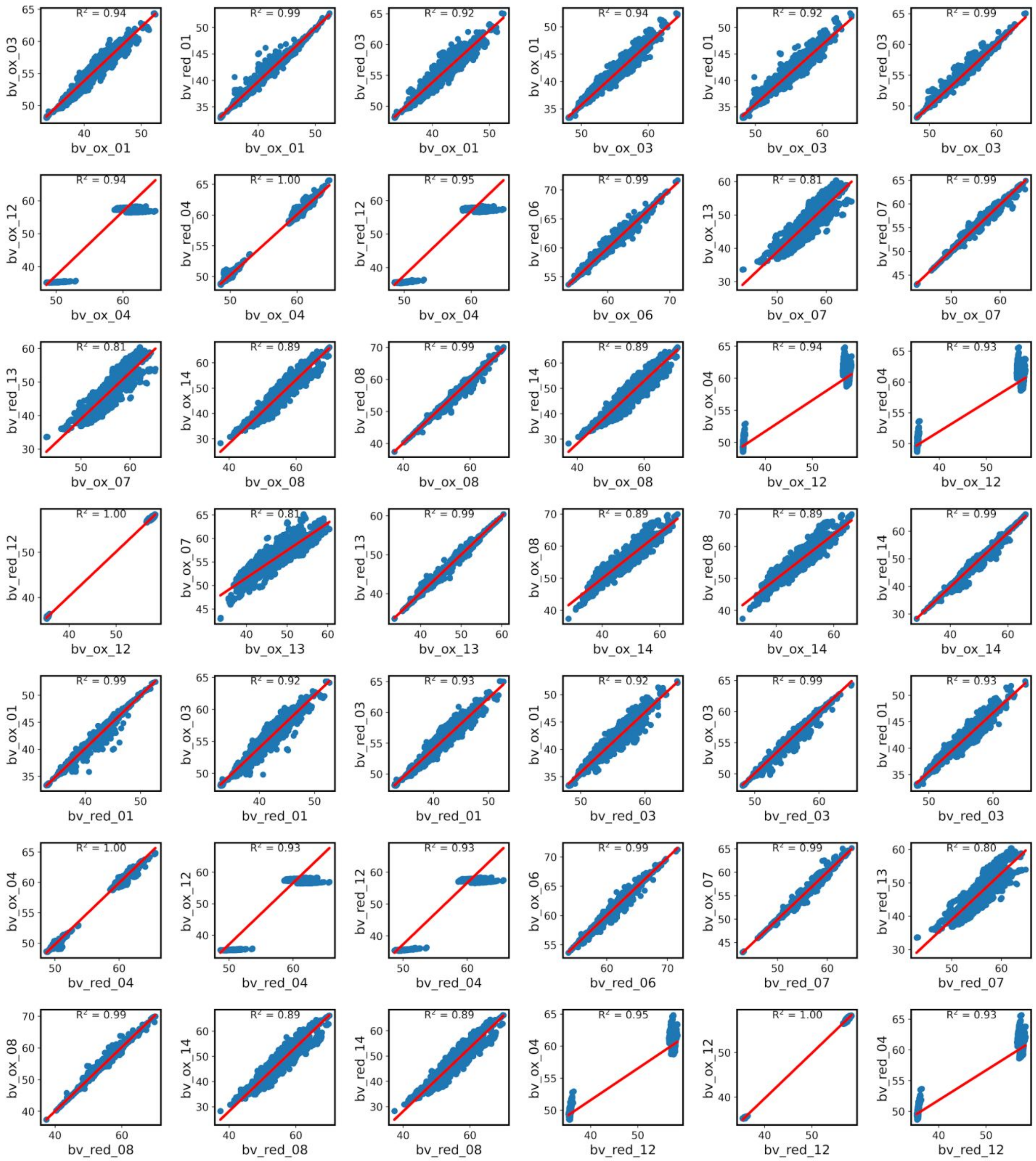
R^2 = 0.94 bv_ox_03 bv_ox_01
R^2 = 0.99 bv_red_01 bv_ox_01
R^2 = 0.92 bv_red_03 bv_ox_01
R^2 = 0.94 bv_ox_01 bv_ox_03
R^2 = 0.92 bv_red_01 bv_ox_03
R^2 = 0.99 bv_red_03 bv_ox_03
R^2 = 0.94 bv_ox_12 bv_ox_04
R^2 = 1.00 bv_red_04 bv_ox_04
R^2 = 0.95 bv_red_12 bv_ox_04
R^2 = 0.99 bv_red_06 bv_ox_06
R^2 = 0.81 bv_ox_13 bv_ox_07
R^2 = 0.99 bv_red_07 bv_ox_07
R^2 = 0.81 bv_red_13 bv_ox_07
R^2 = 0.89 bv_ox_14 bv_ox_08
R^2 = 0.99 bv_red_08 bv_ox_08
R^2 = 0.89 bv_red_14 bv_ox_08
R^2 = 0.94 bv_ox_04 bv_ox_12
R^2 = 0.93 bv_red_04 bv_ox_12
R^2 = 1.00 bv_red_12 bv_ox_12
R^2 = 0.81 bv_ox_07 bv_ox_13
R^2 = 0.99 bv_red_13 bv_ox_13
R^2 = 0.89 bv_ox_08 bv_ox_14
R^2 = 0.89 bv_red_08 bv_ox_14
R^2 = 0.99 bv_red_14 bv_ox_14
R^2 = 0.99 bv_ox_01 bv_red_01
R^2 = 0.92 bv_ox_03 bv_red_01
R^2 = 0.93 bv_red_03 bv_red_01
R^2 = 0.92 bv_ox_01 bv_red_03
R^2 = 0.99 bv_ox_03 bv_red_03
R^2 = 0.93 bv_red_01 bv_red_03
R^2 = 1.00 bv_ox_04 bv_red_04
R^2 = 0.93 bv_ox_12 bv_red_04
R^2 = 0.93 bv_red_12 bv_red_04
R^2 = 0.99 bv_ox_06 bv_red_06
R^2 = 0.99 bv_ox_07 bv_red_07
R^2 = 0.80 bv_red_13 bv_red_07
R^2 = 0.99 bv_ox_08 bv_red_08
R^2 = 0.89 bv_ox_14 bv_red_08
R^2 = 0.89 bv_red_14 bv_red_08
R^2 = 0.95 bv_ox_04 bv_red_12
R^2 = 1.00 bv_ox_12 bv_red_12
R^2 = 0.93 bv_red_04 bv_red_12

S3.3 Study of stability: radical dimerization

To investigate the radical dimerization process, we considered that the radical species derived from the one-electron reduction of the phthalimide undergoes protonation, generating the monomeric intermediate responsible for dimer formation. To ensure that the structures used in the analysis represent energetically favorable conformations, a thorough conformational search was carried out using the CREST algorithm at the GFN2-xTB level. The lowest-energy conformer for each derivative was subsequently optimized using Density Functional Theory (DFT) with the M06-2X functional and the def2-TZVP basis set, as implemented in ORCA 5.0.4. Harmonic vibrational frequency calculations confirmed the nature of the stationary points as minima. All DFT computations included implicit solvation effects using the conductor-like polarizable continuum model (CPCM) for acetonitrile. This computational protocol enabled a reliable comparison of structural features across different derivatives, providing a foundation for identifying linear free energy relationships (LFERs) that can support extrapolation of dimerization reactivity trends within the broader phthalimide chemical space.

S3.1.1 Gibbs Free Energy of Radical Dimerization. Energies in Hartree.

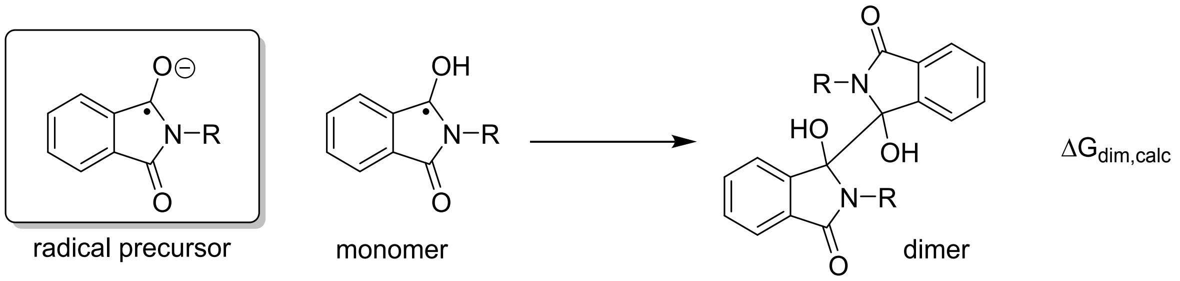

	**monomer**		**dimer**		
**Radical Precursor**	**E(el)**	**G**	**E(el)**	**G**	**$\Delta G_{dim,calc}$**
0221_r2r4_OCH3_C6H5	-973.7546294	-973.5250454	-1947.5940810	-1947.1001672	-31.4234607
0260_r1r5_CH3_C6H4-p-NO2	-1027.8425597	-1027.6216224	-2055.7631210	-2055.2843708	-25.8069620
0363_r2r4_NHCH3_CH3	-742.2964225	-742.0911115	-1484.6768358	-1484.2327525	-31.7077287
0469_r2r4_NHCH3_CH2CH(CH3)2	-860.2242158	-859.9374328	-1720.5274669	-1719.9199276	-28.2768080
0616_r2r4_NO2_C6H5	-1153.7254811	-1153.5572834	-2307.5071009	-2307.1365489	-13.7939515
0633_r2r4_NHC6H4-OCH3_C6H5	-1546.5543167	-1546.1400098	-3093.2045965	-3092.3390199	-37.0232550

0697_r2r4_HN-CO(CH3)_CH3	-969.0303365	-968.8104843	-1938.1324607	-1937.6578848	-23.1652411
0724_r1r5_CHO2(CH2)2_C6H5	-1279.0408579	-1278.7327013	-2558.1422314	-2557.4904399	-15.7110871
0731_r2r4_CHO2(CH2)2_C6H4-p-NO2	-1483.5540947	-1483.2462442	-2967.1901107	-2966.5379287	-28.5141823
0808_r1r5_OCH3_C6H4-p-NO2	-1178.2784082	-1178.0484090	-2356.6237363	-2356.1284219	-19.8317161
0924_r2r4_CHO2(CH2)2_CH2CH(CH3)2	-1205.2317337	-1204.8920497	-2410.5375558	-2409.8213244	-23.3590788
0950_r2r4_NHCH3_C6H4-p-N(CH2)4	-1145.4100453	-1145.0502640	-2290.9057132	-2290.1517231	-32.1254430
1017_r1r5_N(CH2)4_C6H4-p-N(CH2)4	-1378.8326406	-1378.3467046	-2757.7382567	-2756.7295550	-22.6818141
1084_r1r5_OCH3_C6H4-p-N(CH2)4	-1185.1408213	-1184.8064604	-2370.3472987	-2369.6421703	-18.3542889
1422_r1r5_3-py_CH2CH(CH3)2	-1165.0750354	-1164.7444538	-2330.2128590	-2329.5156411	-16.7755378
1463_r1r5_OCH3_CH3	-782.0274820	-781.8468494	-1564.1201968	-1563.7259924	-20.2645722
1575_r1r5_CH2N(CH2)4_CH3	-1054.3516580	-1053.9641147	-2108.7479818	-2107.9409905	-8.0077240
1581_r2r4_HC=N-CH2CH(CH3)2_CH3	-1054.3268851	-1053.9537402	-2108.7238089	-2107.9421352	-21.7462539
1951_r2r4_HC=N-CH2CH(CH3)2_C6H5	-1246.0567004	-1245.6318377	-2492.1886271	-2491.3026423	-24.4520999
2016_r1r5_CH2N(CH3)2_C6H4-p-NO2	-1295.7721638	-1295.4057726	-2591.5877997	-2590.8245879	-8.1844244
2329_r1r5_CH2N(CH3)2_C6H4-p-N(CH2)4	-1302.6311486	-1302.1599584	-2605.3067419	-2604.3314073	-7.2104293
2422_r2r4_CO2CH3_CH2CH(CH3)2	-1126.6784683	-1126.3997813	-2253.4181889	-2252.8281336	-17.9285927
2434_r1r5_CHO_C6H5	-971.3727844	-971.1902832	-1942.7906374	-1942.3872857	-4.2164120
2536_r1r5_3-py_CH3	-1047.1453953	-1046.8966805	-2094.3625474	-2093.8286961	-22.1731486
2563_r1r5_CHO_CH3	-779.6426571	-779.5079843	-1559.3277212	-1559.0258905	-6.2261242
2583_r1r5_NHCH3_CH2CH(CH3)2	-860.2242384	-859.9381643	-1720.5211455	-1719.9151895	-24.3856341
2595_r2r4_N(CH2)4_C6H4-p-N(CH2)4	-1378.8284188	-1378.3429646	-2757.7459808	-2756.7383287	-32.8812280
2639_r2r4_CN_C6H4-p-N(CH2)4	-1140.5907295	-1140.3223374	-2281.2398300	-2280.6702120	-16.0248293
2657_r2r4_CH2OCH3_C6H4-p-N(CH2)4	-1263.7437233	-1263.3533286	-2527.5677379	-2526.7528535	-28.9886548
2934_r1r5_CH2N(CH2)4_CH2CH(CH3)2	-1172.2780550	-1171.8091295	-2344.5996886	-2343.6307489	-7.8375623
2993_r1r5_CO2CH3_C6H4-p-NO2	-1405.0062085	-1404.7603395	-2810.0689988	-2809.5388818	-11.4224237
3117_r2r4_CO-N(CH2)4_C6H4-p-NO2	-1598.7023522	-1598.3057312	-3197.4838155	-3196.6549829	-27.3095021
3203_r2r4_CH3_C6H4-p-NO2	-1027.8438347	-1027.6223311	-2055.7703773	-2055.2942496	-31.1165520
3524_r1r5_CO2CH3_C6H4-p-N(CH2)4	-1411.8695559	-1411.5194952	-2823.7814392	-2823.0410100	-1.2672743
4035_r1r5_OCH3_C6H5	-973.7585417	-973.5275366	-1947.5829114	-1947.0880053	-20.6651742
4400_r1r5_NO2_CH3	-961.9987370	-961.8803351	-1924.0428950	-1923.7712392	-6.6321918
4489_r1r5_CN_C6H4-p-N(CH2)4	-1140.5867574	-1140.3195841	-2281.2372122	-2280.6677125	-17.9118382

4635_r1r5_NO2_CH2CH(CH3)2	-1079.9265376	-1079.7262479	-2159.8942642	-2159.4594635	-4.3723481
4781_r1r5_NHCH3_CH3	-742.2965196	-742.0928015	-1484.6631455	-1484.2208402	-22.1116464
4978_r1r5_CN_CH2CH(CH3)2	-855.4003748	-855.2061624	-1710.8606887	-1710.4385923	-16.4831183
5141_r1r5_NO2_C6H5	-1153.7278820	-1153.5603787	-2307.5035480	-2307.1317194	-6.8787403
5344_r2r4_NO2_CH2CH(CH3)2	-1079.9246601	-1079.7237461	-2159.8965725	-2159.4627523	-9.5758514
5442_r1r5_OCH3_CH2CH(CH3)2	-899.9553213	-899.6927643	-1799.9700947	-1799.4130023	-17.2400454
5588_r1r5_CO2CH3_CH3	-1008.7567138	-1008.5610335	-2017.5598375	-2017.1300834	-5.0303295

S3.1.2 Multivariate Model

Linear regression model (robust fit):
    y ~ 1 + frac_spin_03 + sterimol_B5_R1_ox

Estimated Coefficients:

	Estimate	SE	tStat	pValue
(Intercept)	-18.847	0.59523	-31.664	9.9672e-26
frac_spin_03	-6.7753	0.60567	-11.187	1.3616e-12
sterimol_B5_R1_ox	5.5507	0.60567	9.1647	1.8319e-10

Number of observations: 35, Error degrees of freedom: 32
Root Mean Squared Error: 3.52
R-squared: 0.859, Adjusted R-Squared: 0.85
F-statistic vs. constant model: 97.6, p-value = 2.39e-14

Model_fit =
Linear regression model (robust fit):
    y ~ 1 + x1

Estimated Coefficients:

	Estimate	SE	tStat	pValue
(Intercept)	-2.7752	1.241	-2.2362	0.032216
x1	0.85977	0.060022	14.324	1.0145e-15

Number of observations: 35, Error degrees of freedom: 33
Root Mean Squared Error: 3.34
R-squared: 0.862, Adjusted R-Squared: 0.857
F-statistic vs. constant model: 205, p-value = 1e-15
pred R^2 = 0.75536
Q^2 = 0.82674
Averaged K-fold Q^2 = 0.81812

Table S4. Training Set and Test Set dimerization reaction. Gibbs Free Energies in kca.mol^{-1}.

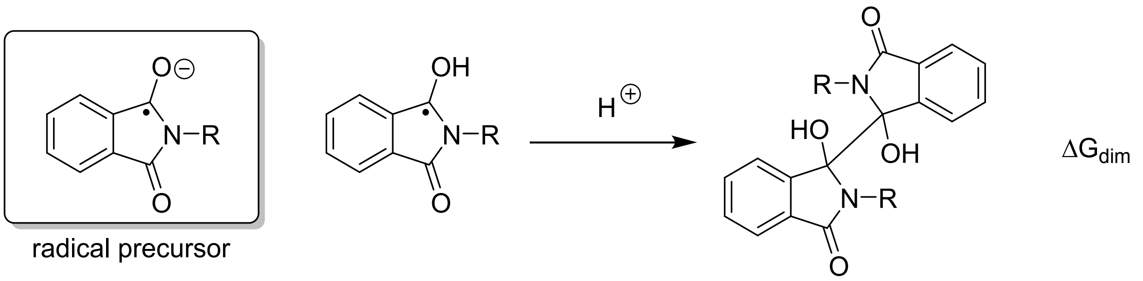

**Radical precursor**	**$\Delta G_{dim,calc}$**	**$\Delta G_{dim,pred}$**
Training Set		
0221_r2r4_OCH3_OCH3_C6H5	-31.42	-31.16
0260_r1r5_CH3_CH3_C6H4-p-NO2	-25.29	-24.17
0363_r2r4_NHCH3_NHCH3_CH3	-31.71	-32.34
0469_r2r4_NHCH3_NHCH3_CH2CH(CH3)2	-28.28	-31.97
0633_r2r4_NHC6H4-OCH3_NHC6H4-OCH3_C6H5	-37.02	-30.27
0697_r2r4_HN-CO(CH3)_HN-CO(CH3)_CH3	-23.17	-27.71
0724_r1r5_CHO2(CH2)2_CHO2(CH2)2_C6H5	-15.71	-14.19
0731_r2r4_CHO2(CH2)2_CHO2(CH2)2_C6H4-p-NO2	-28.51	-27.63
0924_r2r4_CHO2(CH2)2_CHO2(CH2)2_CH2CH(CH3)2	-23.36	-26.60
0950_r2r4_NHCH3_NHCH3_C6H4-p-N(CH2)4	-32.13	-31.85
1017_r1r5_N(CH2)4_N(CH2)4_C6H4-p-N(CH2)4	-22.68	-17.35
1084_r1r5_OCH3_OCH3_C6H4-p-N(CH2)4	-18.35	-17.83
1422_r1r5_3-py_3-py_CH2CH(CH3)2	-16.78	-16.84
1463_r1r5_OCH3_OCH3_CH3	-20.26	-18.38
1575_r1r5_CH2N(CH2)4_CH2N(CH2)4_CH3	-8.01	-9.67
1581_r2r4_HC=N-CH2CH(CH3)2_HC=N-CH2CH(CH3)2_CH3	-21.75	-19.20
2016_r1r5_CH2N(CH3)2_CH2N(CH3)2_C6H4-p-NO2	-8.18	-14.05
2329_r1r5_CH2N(CH3)2_CH2N(CH3)2_C6H4-p-N(CH2)4	-7.21	-13.29
2422_r2r4_CO2CH3_CO2CH3_CH2CH(CH3)2	-17.93	-19.96
2434_r1r5_CHO_CHO_C6H5	-4.22	-5.00
2563_r1r5_CHO_CHO_CH3	-6.23	-5.28
2583_r1r5_NHCH3_NHCH3_CH2CH(CH3)2	-24.39	-19.42
2639_r2r4_CN_CN_C6H4-p-N(CH2)4	-16.02	-17.65
2993_r1r5_CO2CH3_CO2CH3_C6H4-p-NO2	-11.42	-16.20
3117_r2r4_CO-N(CH2)4_CO-N(CH2)4_C6H4-p-NO2	-27.31	-25.45
3203_r2r4_CH3_CH3_C6H4-p-NO2	-31.12	-30.20
3524_r1r5_CO2CH3_CO2CH3_C6H4-p-N(CH2)4	-1.27	-14.19
4035_r1r5_OCH3_OCH3_C6H5	-20.67	-18.15
4400_r1r5_NO2_NO2_CH3	-6.63	-4.85
4489_r1r5_CN_CN_C6H4-p-N(CH2)4	-17.91	-17.49
4635_r1r5_NO2_NO2_CH2CH(CH3)2	-4.37	-4.89

4781_r1r5_NHCH3_NHCH3_CH3	-22.11	-19.90
4978_r1r5_CN_CN_CH2CH(CH3)2	-16.48	-17.51
5141_r1r5_NO2_NO2_C6H5	-6.88	-4.67
5344_r2r4_NO2_NO2_CH2CH(CH3)2	-9.58	-14.31
Test Set		
0616_r2r4_NO2_NO2_C6H5	-13.79	-14.62
0808_r1r5_OCH3_OCH3_C6H4-p-NO2	-19.83	-18.56
1951_r2r4_HC=N-CH2CH(CH3)2_HC=N-CH2CH(CH3)2_C6H5	-24.45	-19.49
2536_r1r5_3-py_3-py_CH3	-22.17	-17.41
2595_r2r4_N(CH2)4_N(CH2)4_C6H4-p-N(CH2)4	-32.88	-31.31
2657_r2r4_CH2OCH3_CH2OCH3_C6H4-p-N(CH2)4	-28.99	-27.54
2934_r1r5_CH2N(CH2)4_CH2N(CH2)4_CH2CH(CH3)2	-7.84	-9.19
5442_r1r5_OCH3_OCH3_CH2CH(CH3)2	-17.24	-18.04
5588_r1r5_CO2CH3_CO2CH3_CH3	-5.03	-15.59

Table S5. Classification of Electron-Withdrawing Groups (EWG) and Electron-Donating Groups (EDG) corresponding to Figure 1c of the manuscript, based on Hammett σ⍰ values. In some cases, structurally similar functional groups were used as representative substitutions.

R^1 or R^2	σ_p[22]	classification
NO_2	0,78	EWG
CHO	0,42	
CN	0,66	
CO_2Et	0,45	
HC=N-Ph	0,42	
$CONMe_2$	0,36	
-3py	0,25	
CH_2OMe	0,01	EDG
CH_2NMe_2	0,01	
NHCOH	0	
CH_3	-0,17	
OCH_3	-0,27	
NMe2	-0,83	

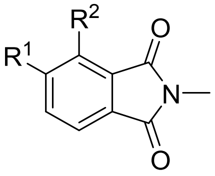

[22] Hansch, C.; Leo, A.; Taft, R. W. *Chem. Rev.* **1991**, *91*, 165–195

S4. Electrochemical Characterization of the Phthalimides Candidates 3', 5 and 6

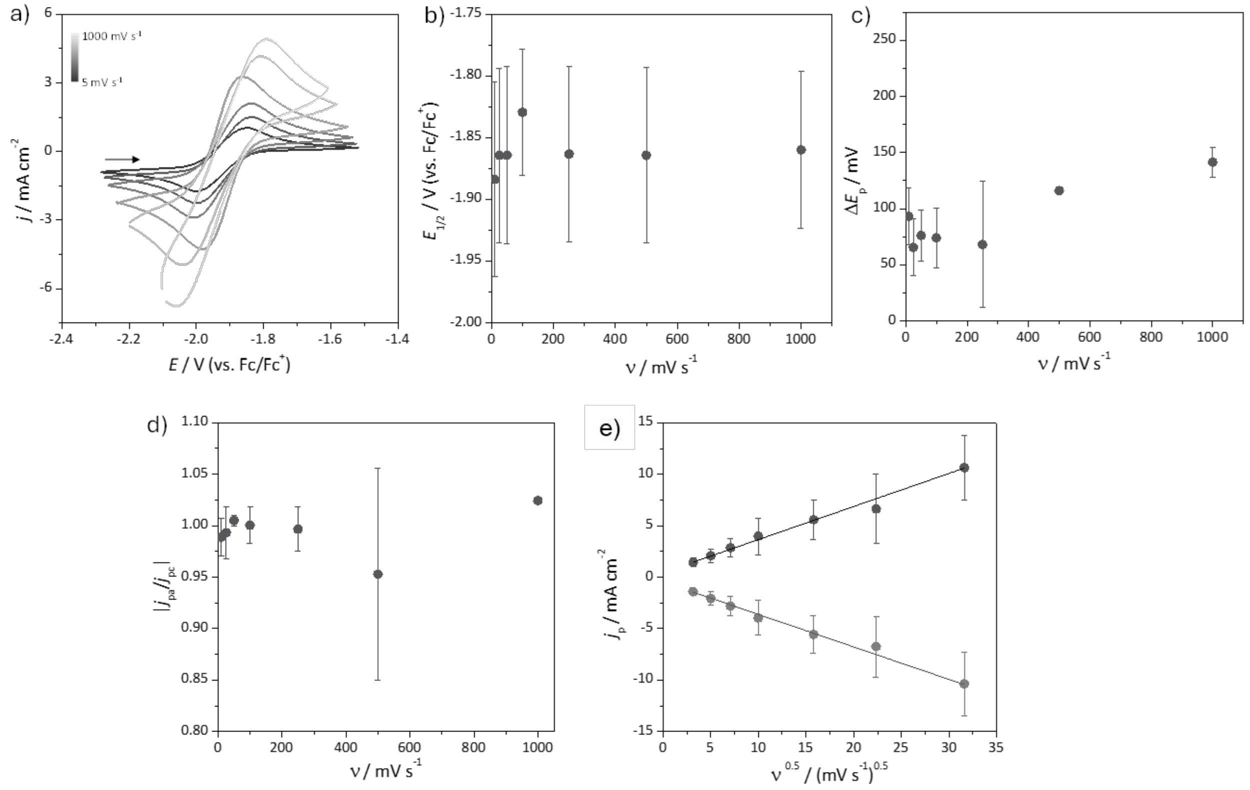

Figure S4. a) Cyclic voltammograms of 10 mmol L^{-1} phthalimide **3'** at 10, 25, 50, 100, 250, 500, and 1000 mV s^{-1}. Dependence of the average and related standard deviation values of (b) $E_{1/2}$, (c) ΔE_p, (d) $|j_{pa}/j_{pc}|$, and (e) j_p with the scan rate for phthalimide **3'** (based on a). All cyclic voltammogram plots are reported according to (IUPAC convention) and recorded in acetonitrile containing 100 mmol L^{-1} TBAP, as electrolyte, under inert atmosphere, at 25.0 ± 0.1 °C, and using glassy carbon disk, Pt plate, and Ag/Ag^+ as working, counter, and reference electrodes respectively. Subsequently, all potentials values were converted against Fc/Fc^+.

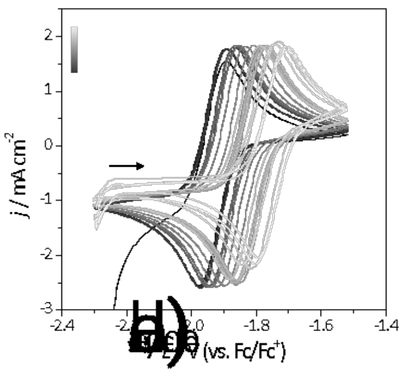

Figure S5. (a) Successive CVs (IUPAC convention) at 50 mV s^{-1} of 10 mmol L^{-1} phthalimide **3'** in acetonitrile containing 100 mmol L^{-1} TBAP. Dependence of the average and related standard deviation values of (b) $E_{1/2}$, (c) ΔE_p, (d) j_p, and (e) $|j_{pa}/j_{pc}|$ with the scan rate (based on a). All measurements were performed under inert atmosphere, at 25.0 ± 0.1 °C, and using glassy carbon disk, Pt plate, and Ag/Ag^+ as working, counter, and reference electrodes respectively. Subsequently, all potentials values were converted against Fc/Fc^+.

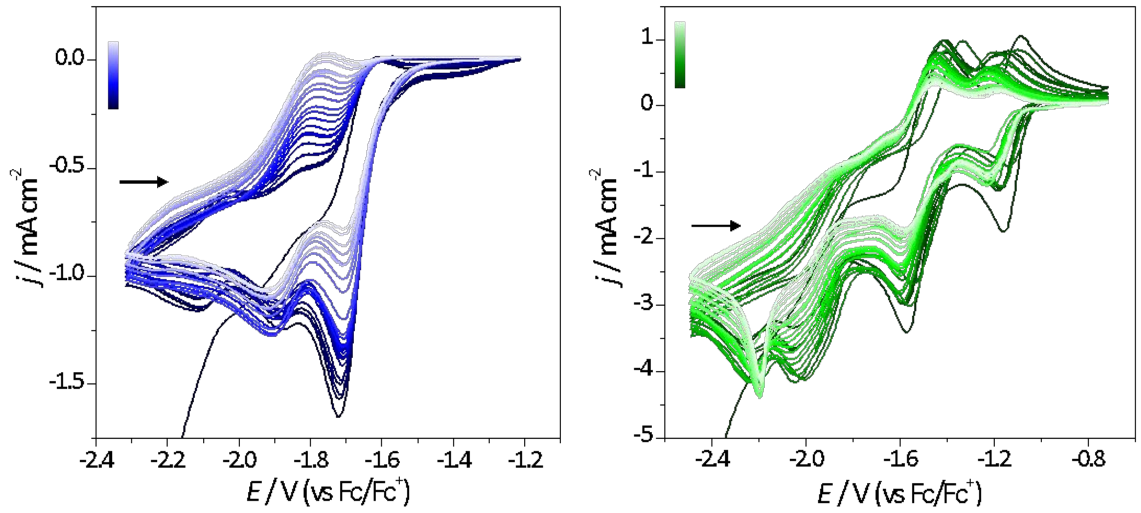

Figure S6. Successive CVs (IUPAC convention) at 50 mV s^{-1} of 10 mmol L^{-1} phthalimides (a) **5** and (b) **6** in acetonitrile containing 100 mmol L^{-1} TBAP. All measurements were performed under inert atmosphere, at 25.0 ± 0.1 °C, and using glassy carbon disk, Pt plate, and Ag/Ag^{+} as working, counter, and reference electrodes respectively. Subsequently, all potentials values were converted against Fc/Fc^{+}.

S5. Diffusion coefficient and electron transfer constant calculation

The phthalimides **8’** and **3’** diffusion coefficient in acetonitrile was calculated by using the Randles–Sevcik equation:

$$j_p = 2.69 \times 10^5 n^{3/2} D^{1/2} C^0 v^{1/2}$$

where, j_p is the peak current density (A cm^{-2}), n is the number of electrons transferred in the reaction, D is the diffusion coefficient (cm^2 s^{-1}), C^0 is the bulk concentration of the redox species in mol cm^{-3}, and v is the scan rate (V s^{-1}).[23] The D can be calculated by the slope of a plot j_p vs. $v^{1/2}$, as:

$$slope = 2.69 \times 10^5 n^{3/2} C D^{1/2}$$

For our system, phthalimides **8’** and **3’** concentration is 1.0 × 10^{-5} mol cm^{-3}, n is 1, and slopes are (9.5 ± 0.2) × 10^{-3} and (1.0 ± 0.1) × 10^{-2} therefore D values are calculated to be 1.25 × 10^{-5} cm^2 s^{-1} and 1.43 × 10^{-5} cm^2 s^{-1}, respectively.

The electron transfer rate constant (k^0) was calculated, according to Nicholson’s analysis, as previously reported.[23,24] Initially, the dimensionless Nicolson number (Ψ) was calculated, as follows:

$$\Psi = \frac{-0.6288 + 0.00021\Delta E_p}{1 - 0.017\Delta E_p}$$

Then, Ψ was used to estimate k^0, according to the following equation:

$$\Psi = k^0 (\pi D n F/RT)^{-1/2} v^{-1/2}$$

where, F is Faraday constant (96,485 C mol^{-1}), R is the gas constant (8.314 J K^{-1} mol^{-1}), and T is the absolute temperature (298 K). Based on the cyclic voltammograms at 50 mV s^{-1}, k^0 were calculated being 5.5 × 10^{-3} s^{-1} and 1.9 × 10^{-2} s^{-1} phthalimides **1** and **2**, respectively.

S6. Electrolyte spectroscopic characterization after galvanostatic cycling

The solutions comprising 10 mmol L^{-1} phthalimides **8’**, **3’**, **5**, and **6** in acetonitrile containing 100 mmol L^{-1} TBAP were analyzed by UV-Vis before and after to be submitted to a charge/discharging cycle. The UV-Vis measurements were performed in a Cary 5G spectrophotometer, by using a

---

[23] Chola, N. M.; Nagarale, R. K. Evaluation and Degradation Mechanism of Phthalimide Derivatives as Anolytes for Non-Aqueous Organic Static Batteries. *New J. Chem.* **2022**, *46*, 22593–22601.

[24] Hu, B.; Liu, T. L. Two-Electron Utilization of Methyl Viologen Anolyte in Nonaqueous Organic Redox Flow Battery. *J. Energy Chem.* **2018**, *27*, 1326–1332.

quartz cuvette with 1 cm path length. Background was recorded in acetonitrile containing 100 mmol L^{-1} TBAP.

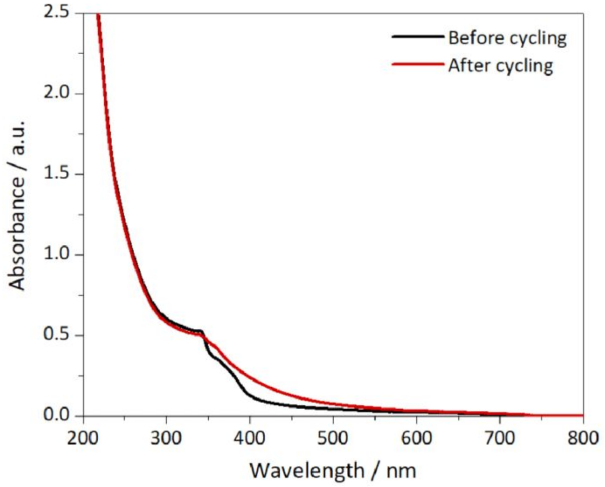

Figure S7. UV-Vis and of 10 mmol L^{-1} phthalimide **1** in acetonitrile containing 100 mmol L^{-1} TBAP, before and after galvanostatic cycling.

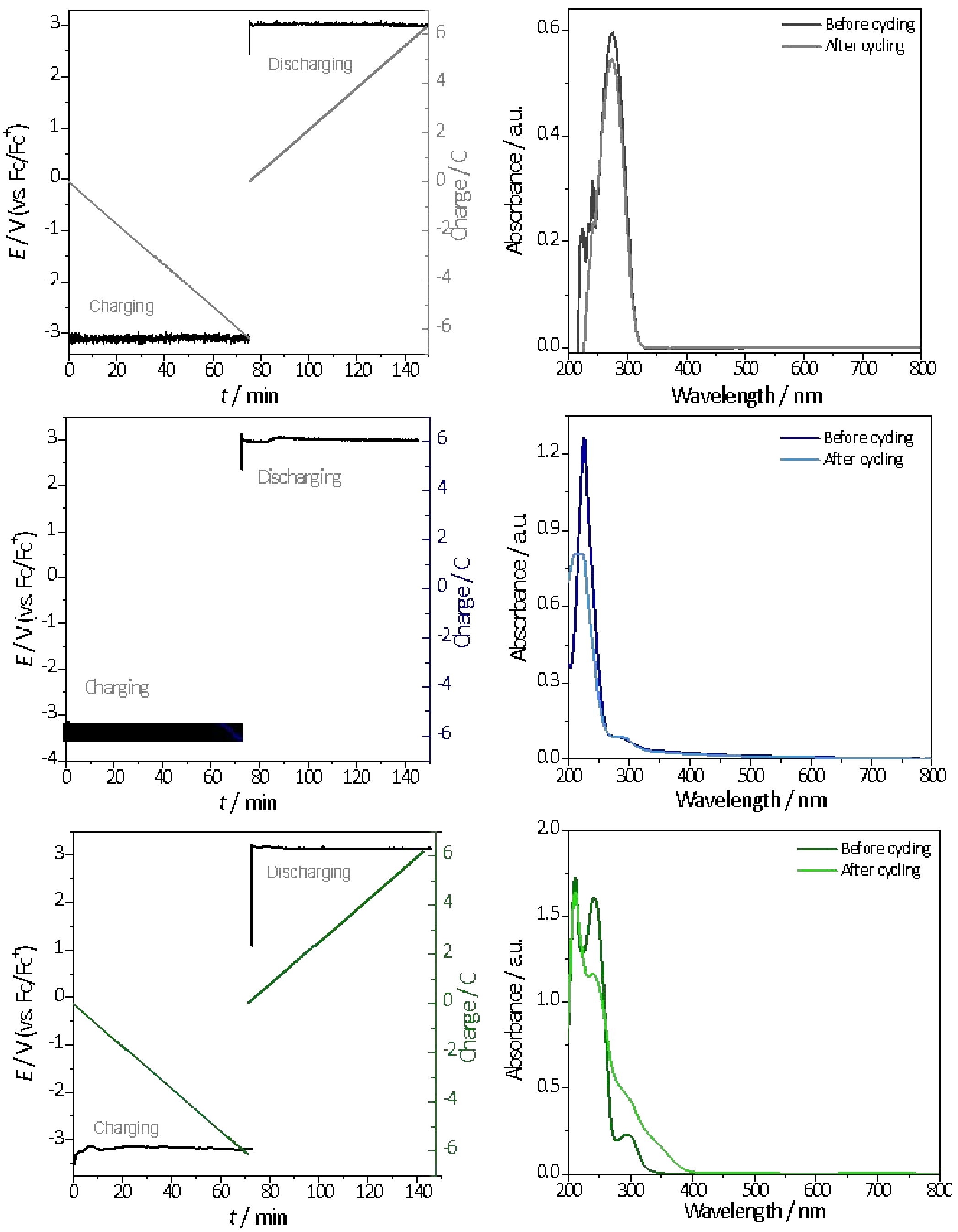

Figure S8. a-c) Galvanostatic charging/discharging cycle of 10 mmol L^{-1} phthalimides **3', 5** and **6** in acetonitrile containing 100 mmol L^{-1} TBAP at ±20 mA cm^{-2} and 1000 rpm, inert atmosphere, 25.0 ± 0.1 ^{o}C, and using glassy carbon disk, Pt plate, and Ag/Ag^{+} as working, counter, and reference electrodes respectively. Subsequently, all potentials values were converted against Fc/Fc^{+}. d-f) UV-Vis spectra of phthalimides **3', 5** and **6** solutions before and after galvanostatic cycling.

S7. Experimental informations of the CEM Discover® focused microwave

Reaction

Teste 18112429 Rafaela-male   User: cem   35mL Vessel   Snap Cap

Method Parameters

Name: Rafaela-male

Type: Conventional

**Temp(C):**	120
**Time(mm:ss):**	30:00

Graphs

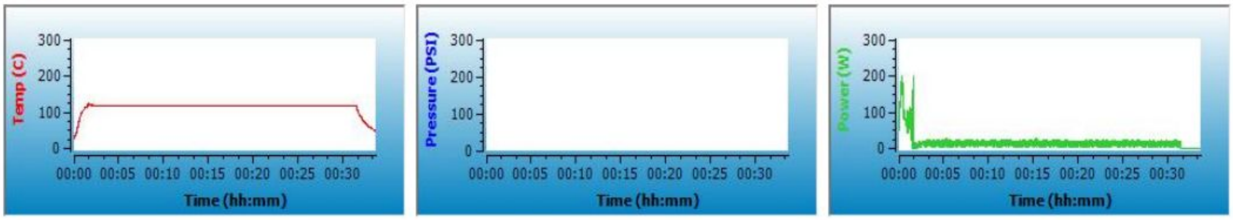

Method Summary

Reaction started:	2/25/2025 9:29:30 AM
Temperature setpoint reached:	2/25/2025 9:30:59 AM
Reaction cooling started:	2/25/2025 10:00:59 AM
Cooling/Reaction ended:	2/25/2025 10:03:08 AM

Reaction Completed Successfully!

Maximum temperature:	127 C
Maximum pressure:	0 PSI
Time to obtain setpoint:	01:29 mm:ss
Time at setpoint:	30:00 mm:ss
Time cooling:	02:09 mm:ss

Figure S9. Data report of the synthesis of N-isopentylmaleimide, irradiated for 30 minutes in a CEM Discover® focused microwave reactor at 120 °C.

Reaction

Teste 181124160 Rafaela User: cem 10mL Vessel Snap Cap

Method Parameters

Name: Rafaela

Type: Conventional

**Temp(C):**	200
**Time(mm:ss):**	10:00

Graphs

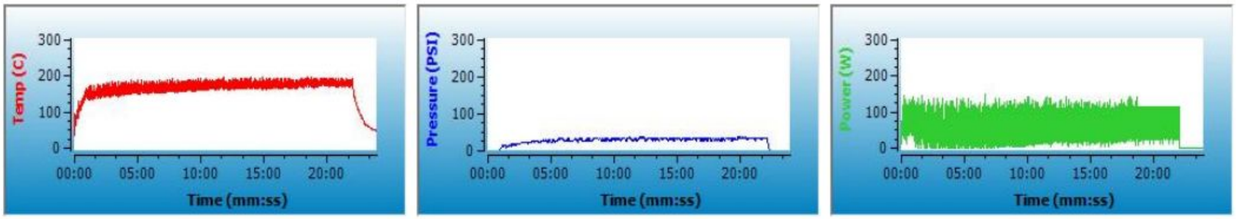

Method Summary

Reaction started: 10/22/2025 3:14:39 PM
Reaction cooling started: 10/22/2025 3:36:53 PM
Cooling/Reaction ended: 10/22/2025 3:38:44 PM

Reaction Completed Successfully!

Maximum temperature: 198 C
Maximum pressure: 35 PSI
Time to try to obtain setpoint: 22:14 mm:ss
Time cooling: 01:51 mm:ss

Figure S10. Data report of the synthesis of 2-Isopentylisoindoline-1,3-dione and 2-methylisoindoline-1,3-dione (both reactions were carried out using the same microwave procedure), irradiated for 10 minutes in a CEM Discover® focused microwave reactor at 200 °C.

S8. Two-dimensional PCA projection of the phthalimide chemical space

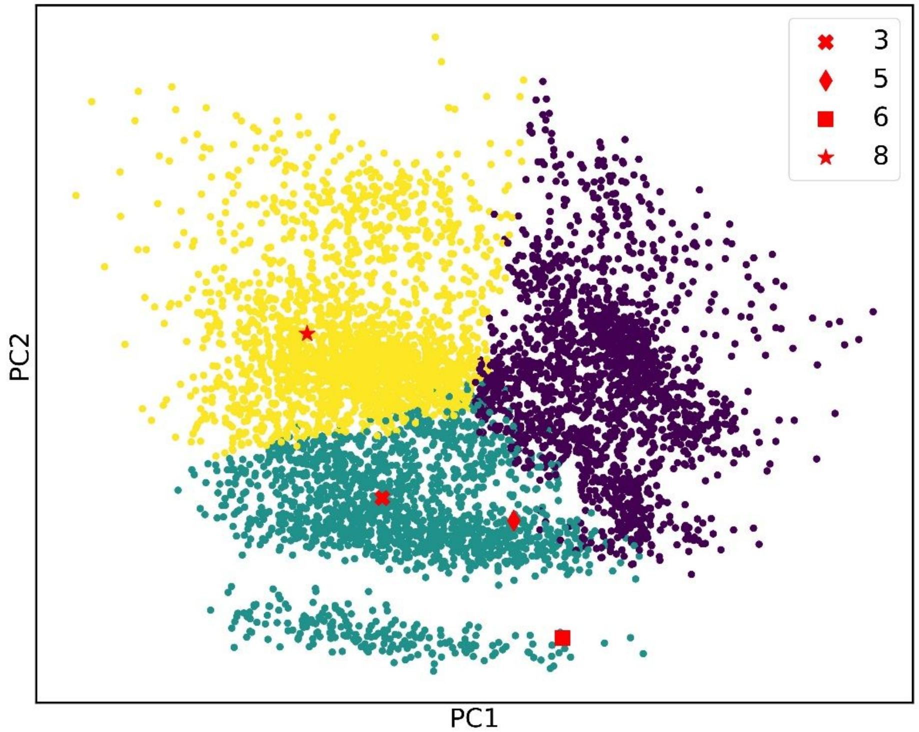

Figure S11. Two-dimensional PCA projection of the phthalimide chemical space showing the same cluster assignment used in Figure 8a. The experimentally investigated compounds (**8′**, **3′**, **5**, and **6**) are highlighted to indicate their location relative to the yellow (top-performing) and green (non-top-performing) regions of the design space.

S9. Spectra for synthesized phthalimides and intermediates

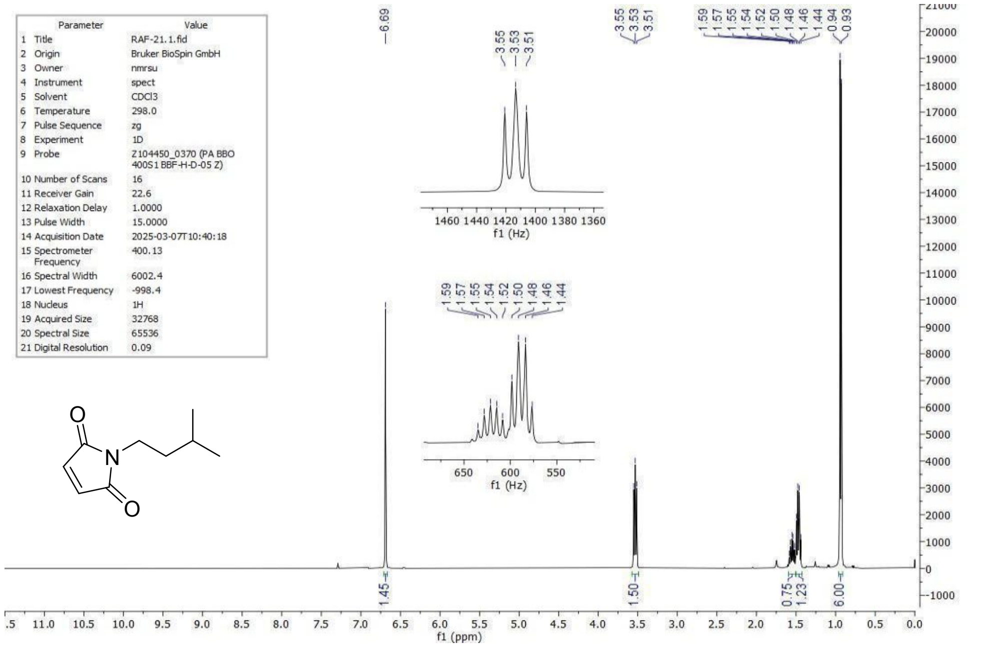

^{1}H NMR spectra (400 MHz, $CDCl_3$) of compound N-isopentylmaleimide **13**.

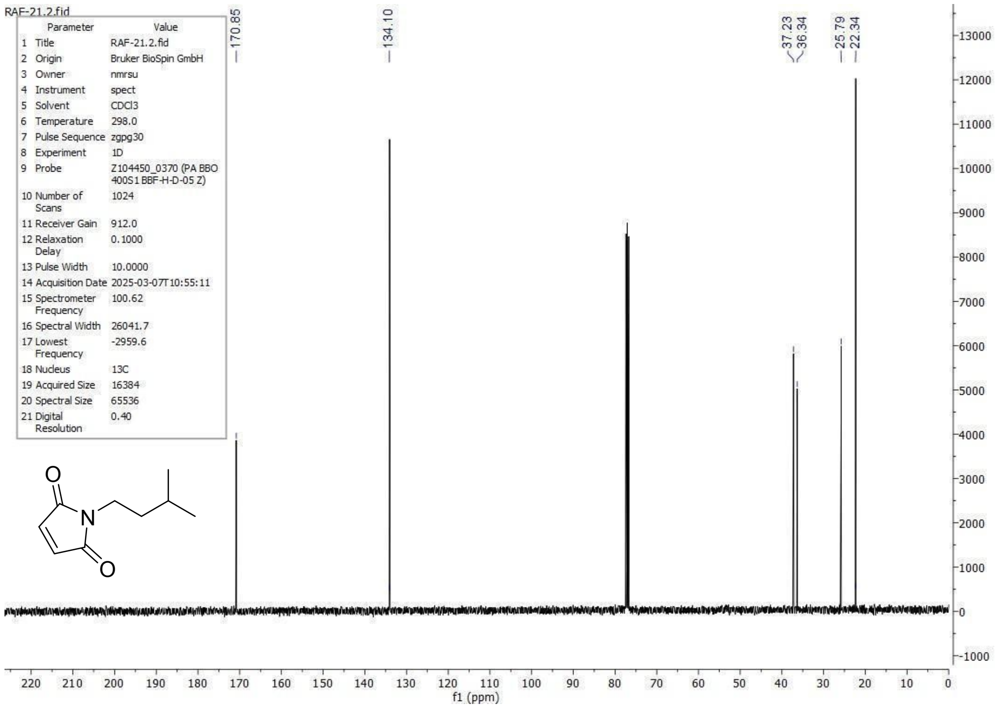

$^{13}C\{^{1}H\}$ spectra (100 MHz, $CDCl_3$) of compound N-isopentylmaleimide **13**.

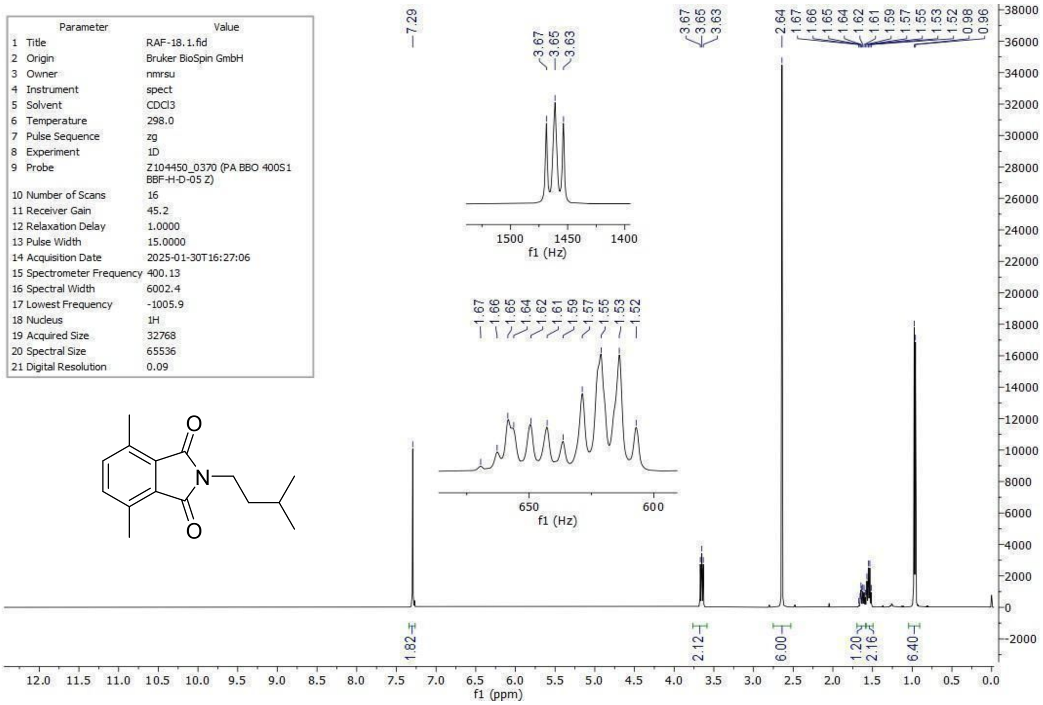

^{1}H NMR spectra (400 MHz, $CDCl_3$) of phthalimide **8’**.

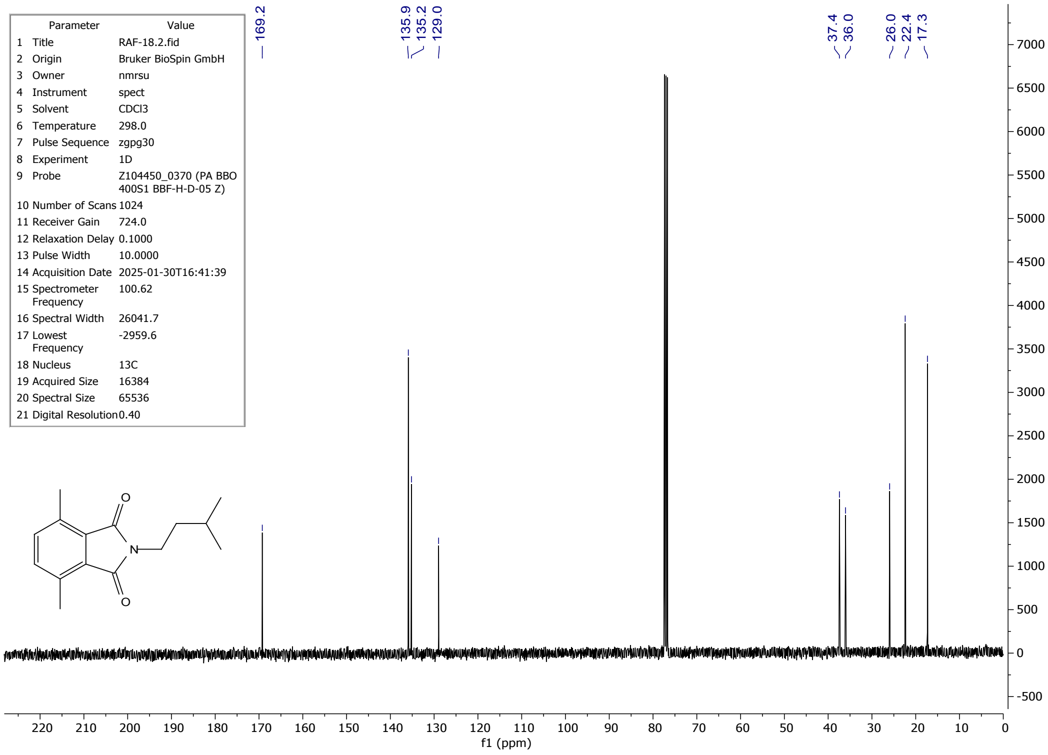

^{13}C{^{1}H} spectra (100 MHz, $CDCl_3$) of phthalimide **8’**.

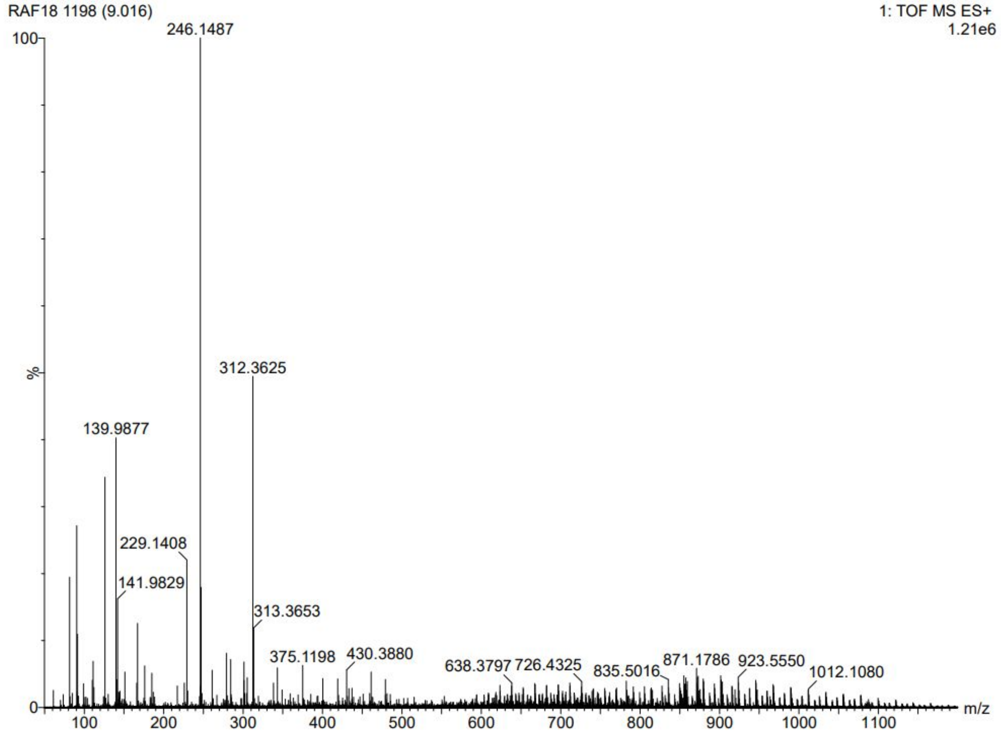

High resolution mass spectra with electrospray ionization of phthalimide **8’**.

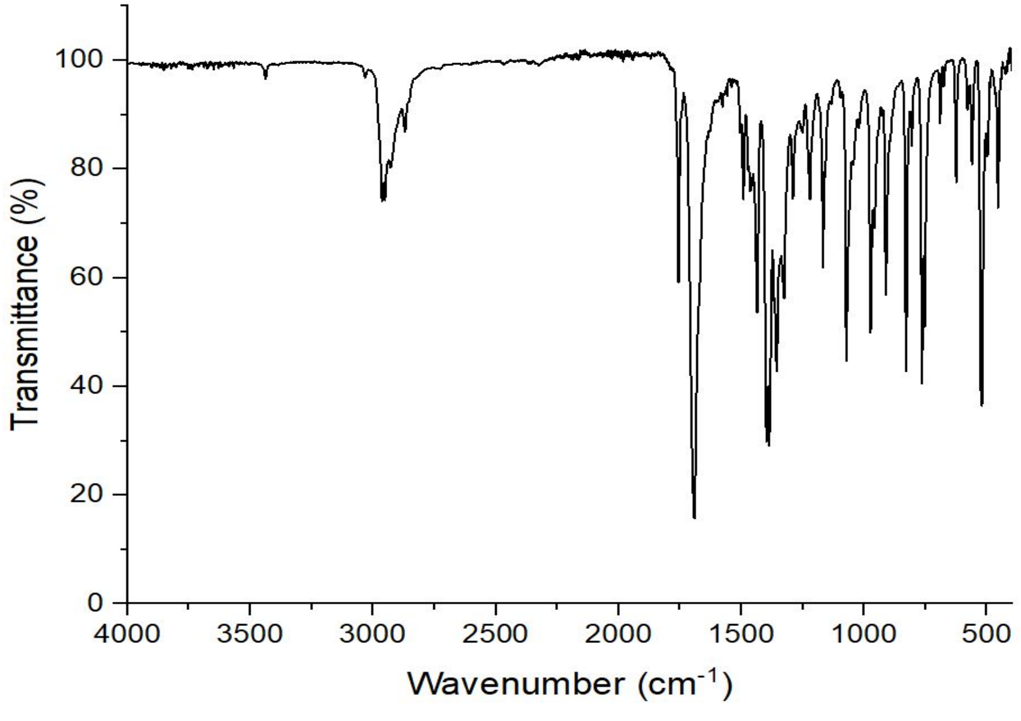

Infrared spectra of phthalimide **8’**.

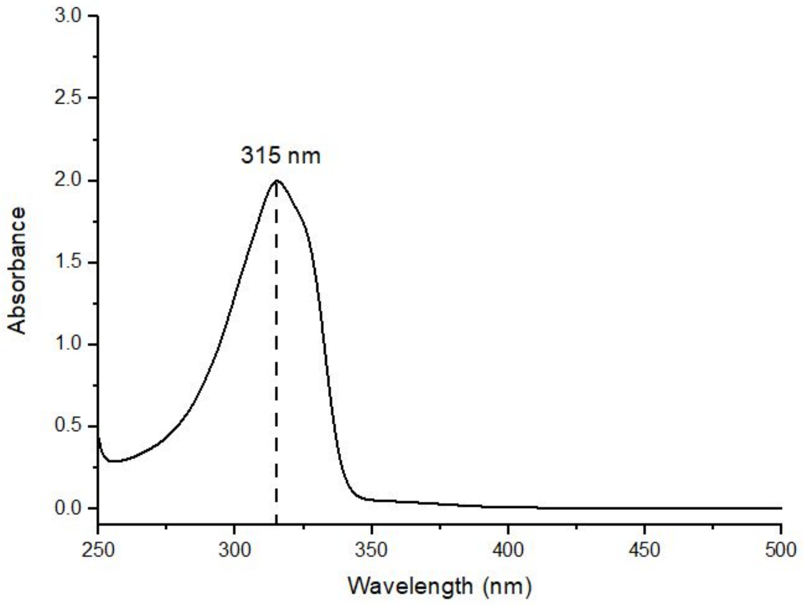

UV-Vis absorption spectra of phthalimide **8’**.

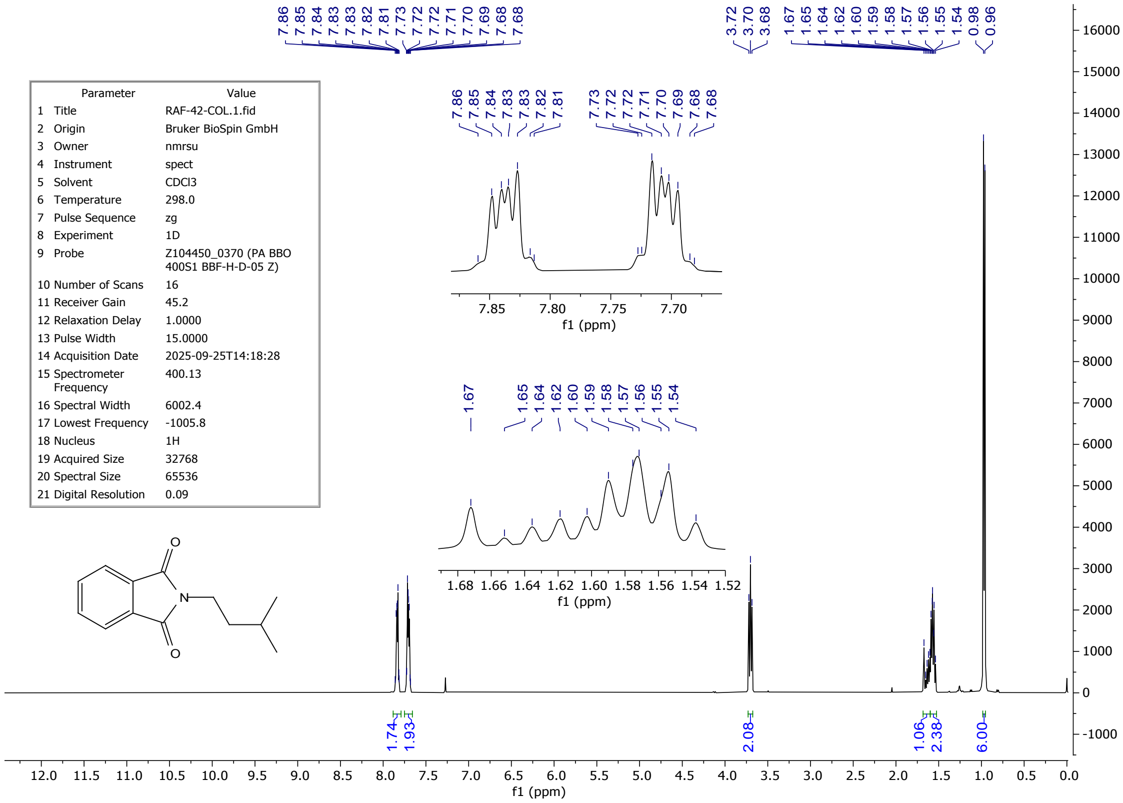

^{1}H NMR spectra (400 MHz, $CDCl_3$) of phthalimide **3’**.

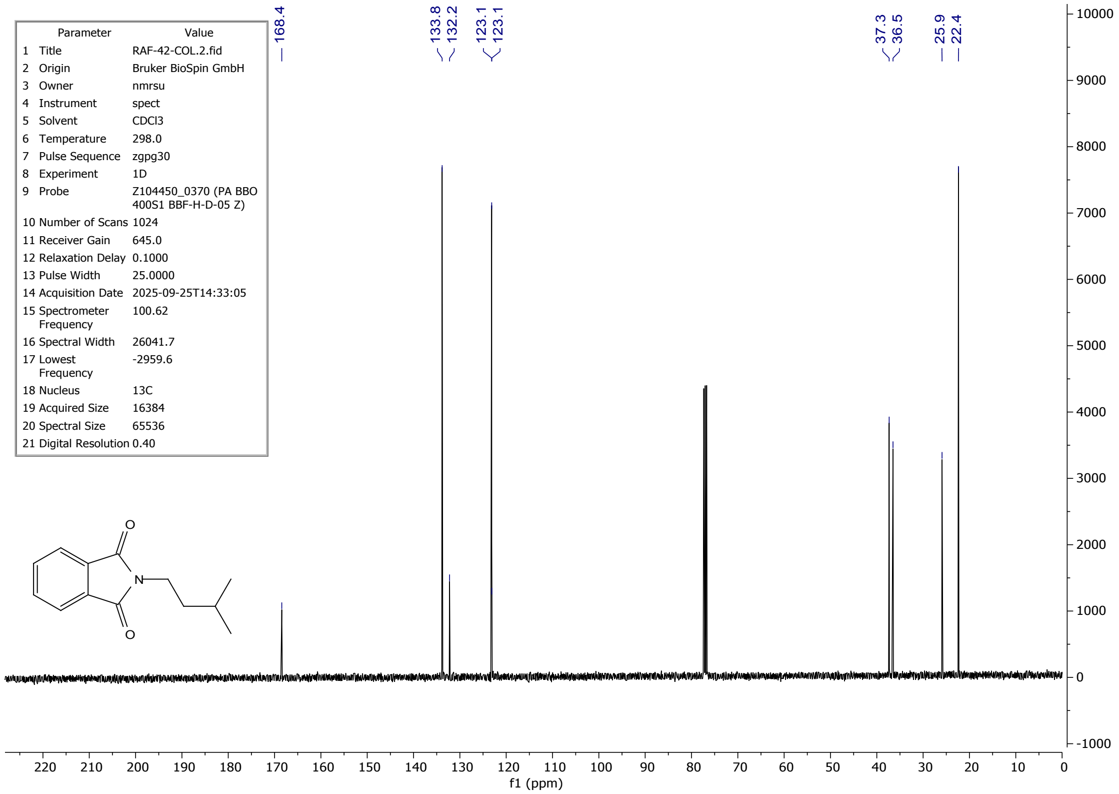

$^{13}C\{^{1}H\}$ spectra (100 MHz, $CDCl_3$) of phthalimide **3’**.

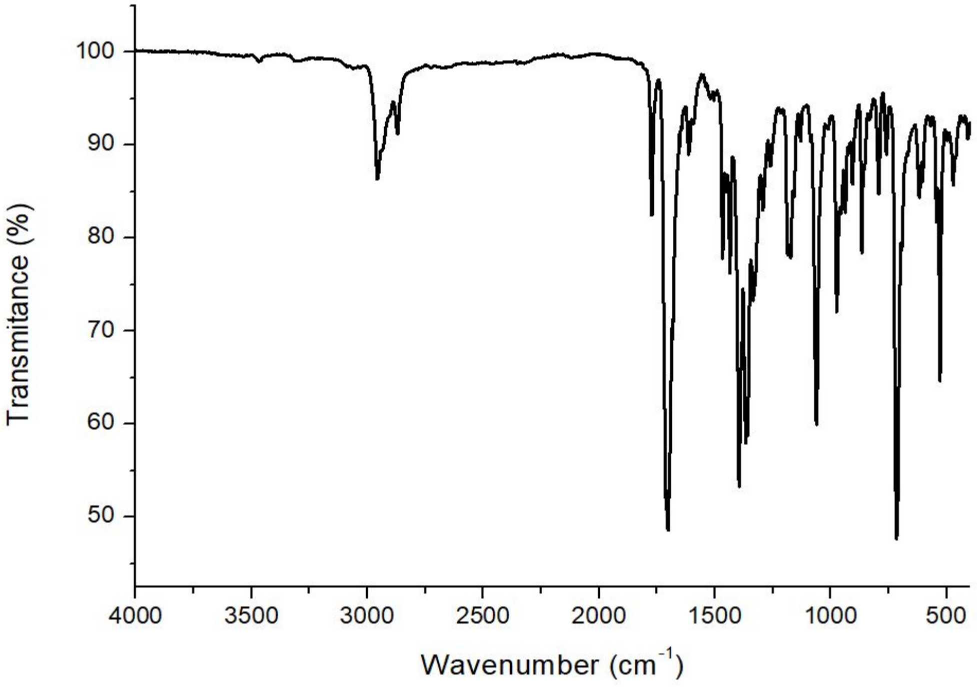

Infrared spectra of phthalimide **3’**.

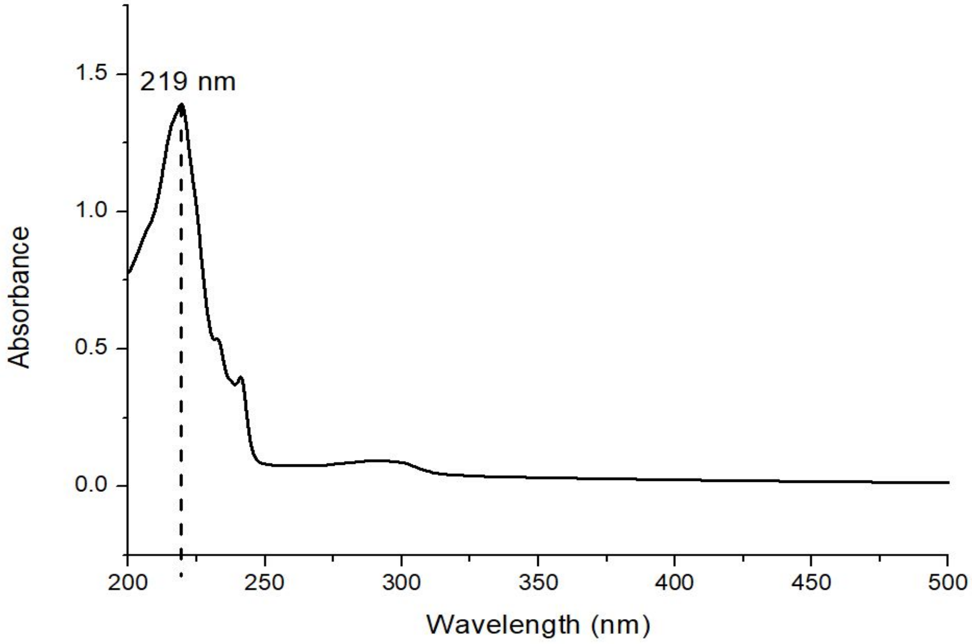

UV-Vis absorption spectra of phthalimide **3’**.

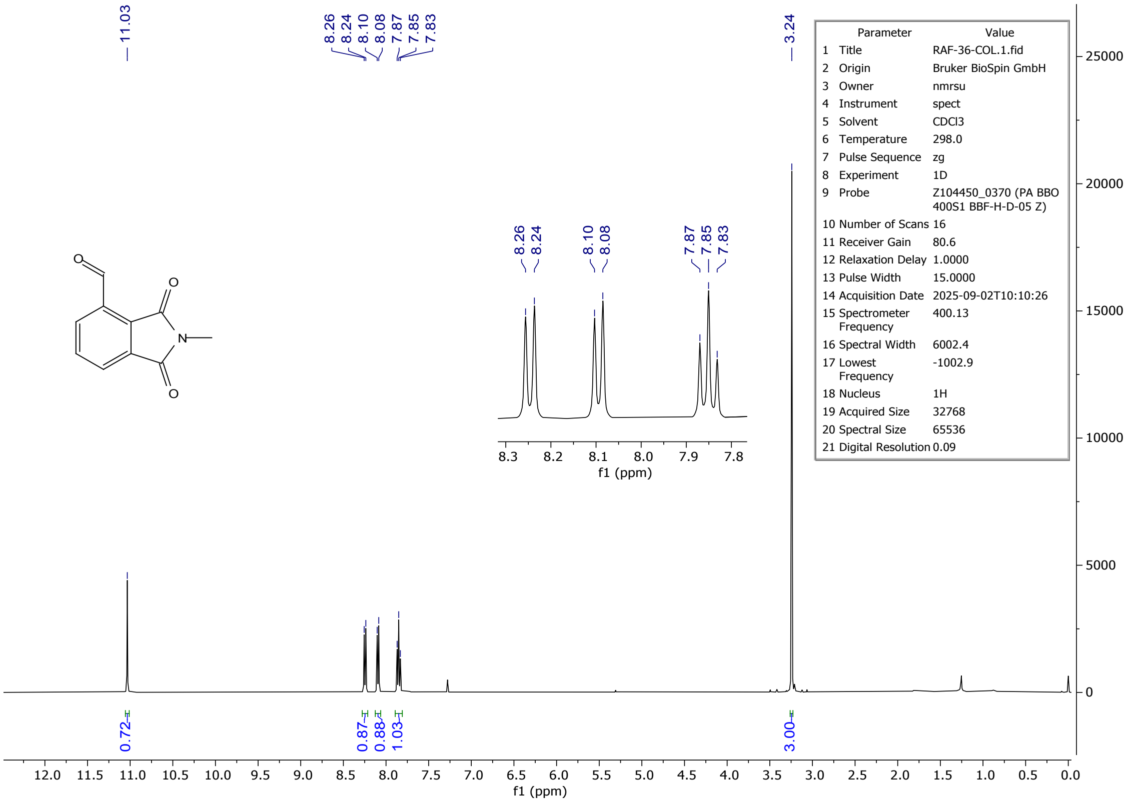

^{1}H NMR spectra (400 MHz, $CDCl_3$) of phthalimide **5**.

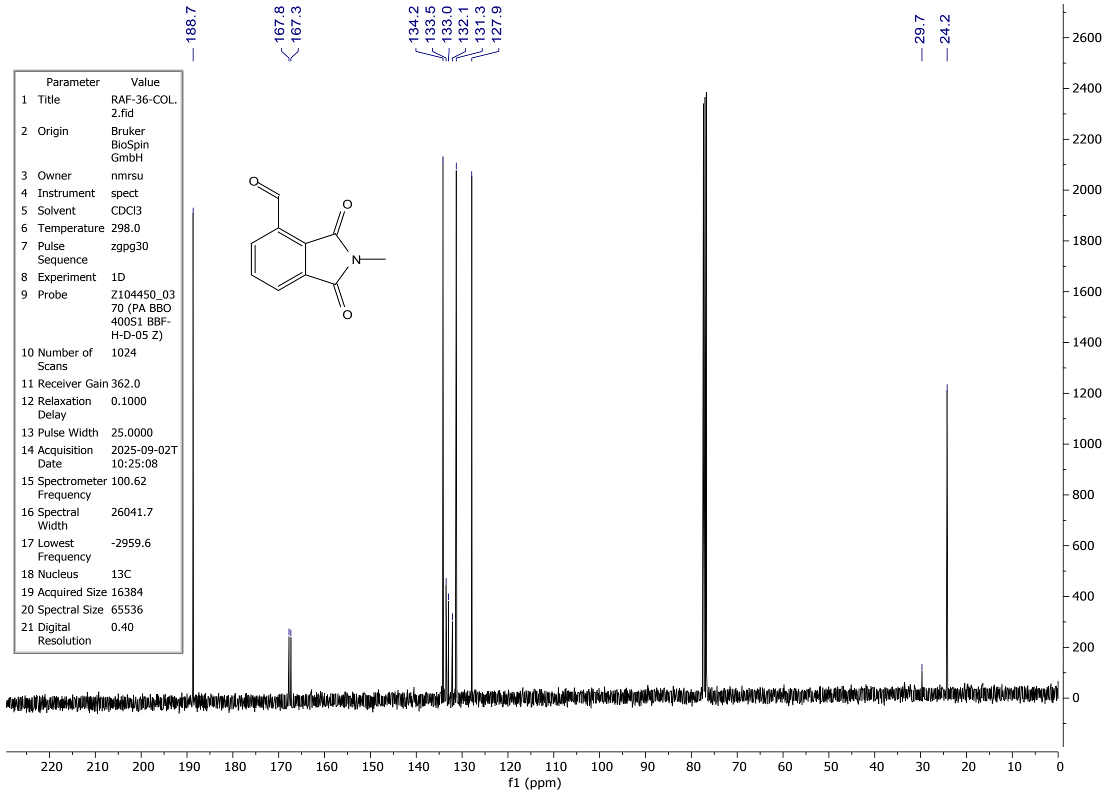

$^{13}C\{^{1}H\}$ spectra (100 MHz, $CDCl_3$) of phthalimide **5**.

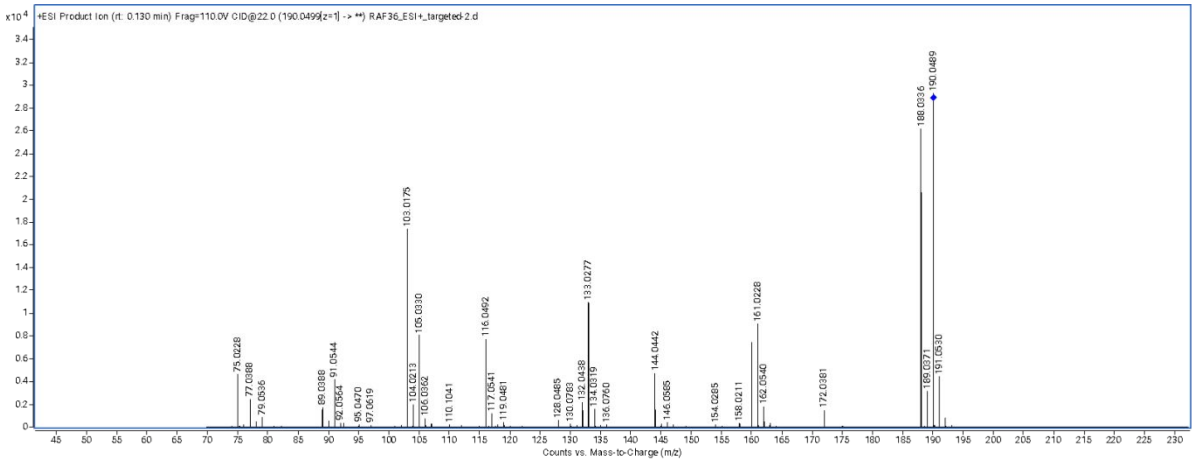

High resolution mass spectra with electrospray ionization of phthalimide **5**.

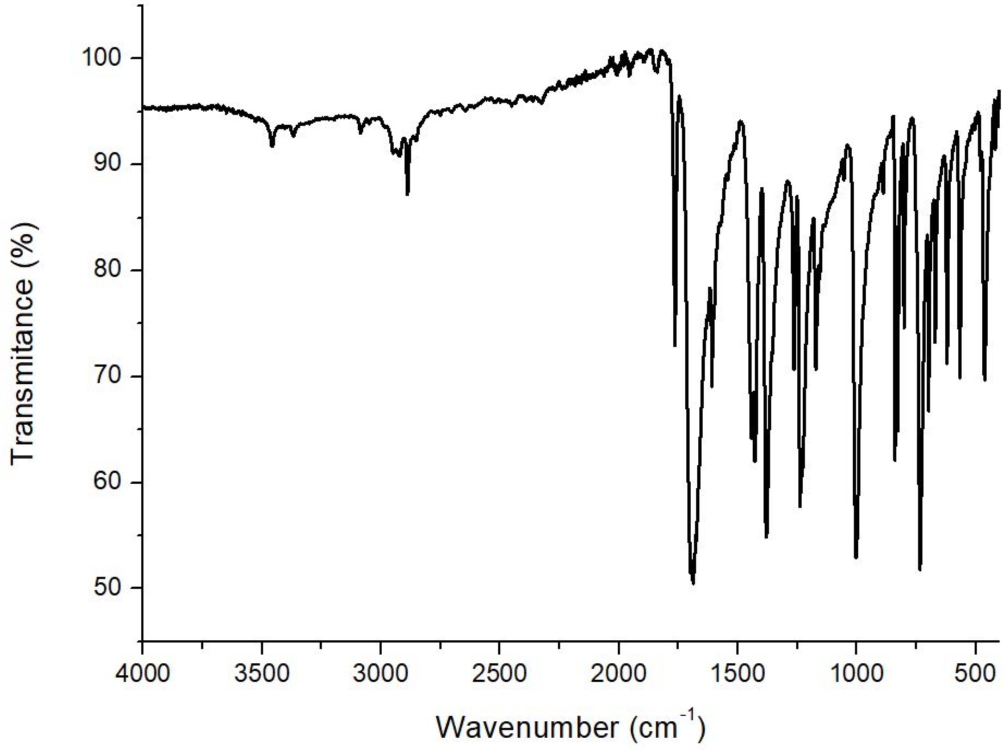

Infrared spectra of phthalimide **5**.

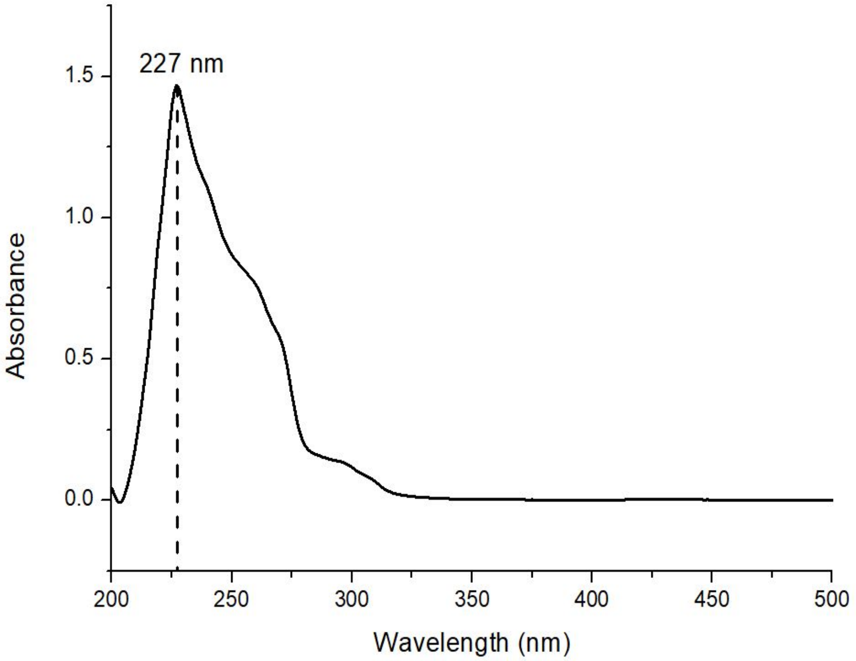

UV-Vis absorption spectra of phthalimide **5**.

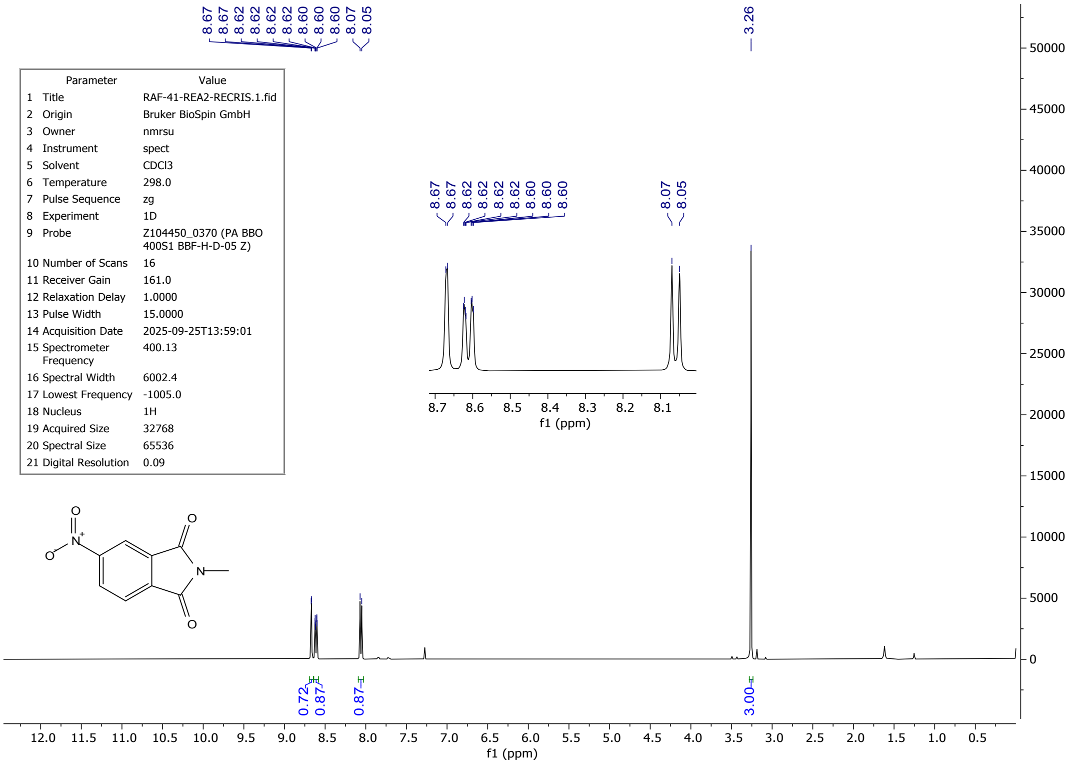

^{1}H NMR spectra (400 MHz, $CDCl_3$) of phthalimide **6**.

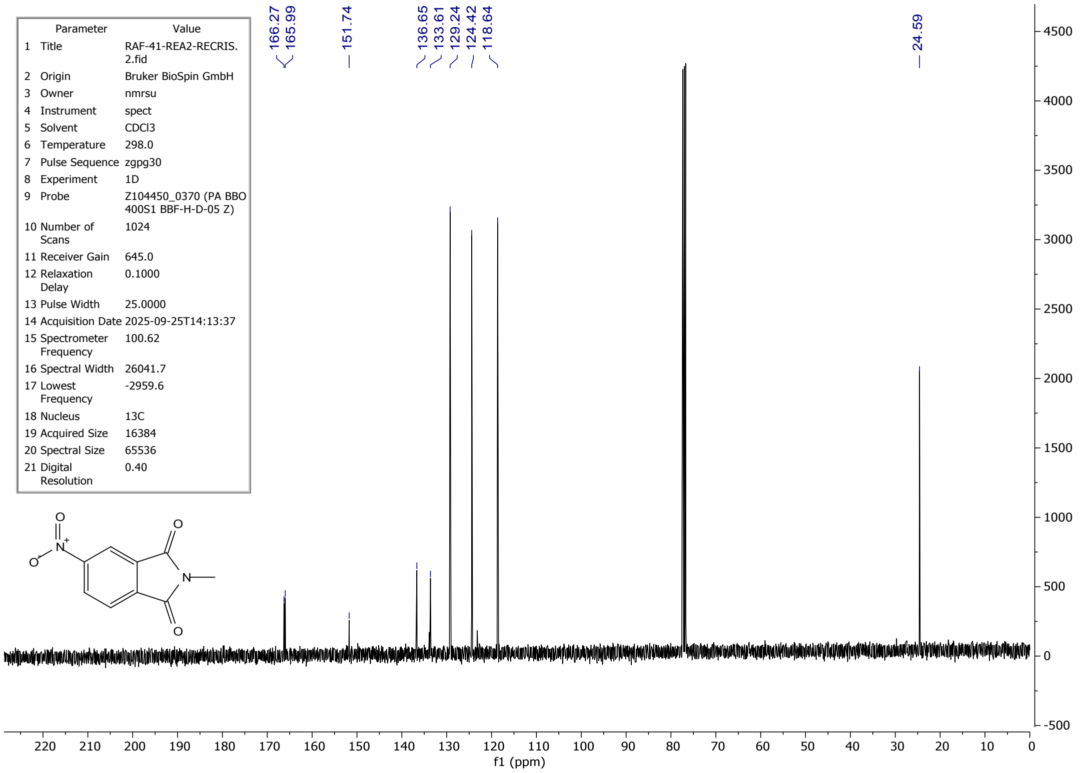

$^{13}C\{^{1}H\}$ spectra (100 MHz, $CDCl_3$) of phthalimide **6**.

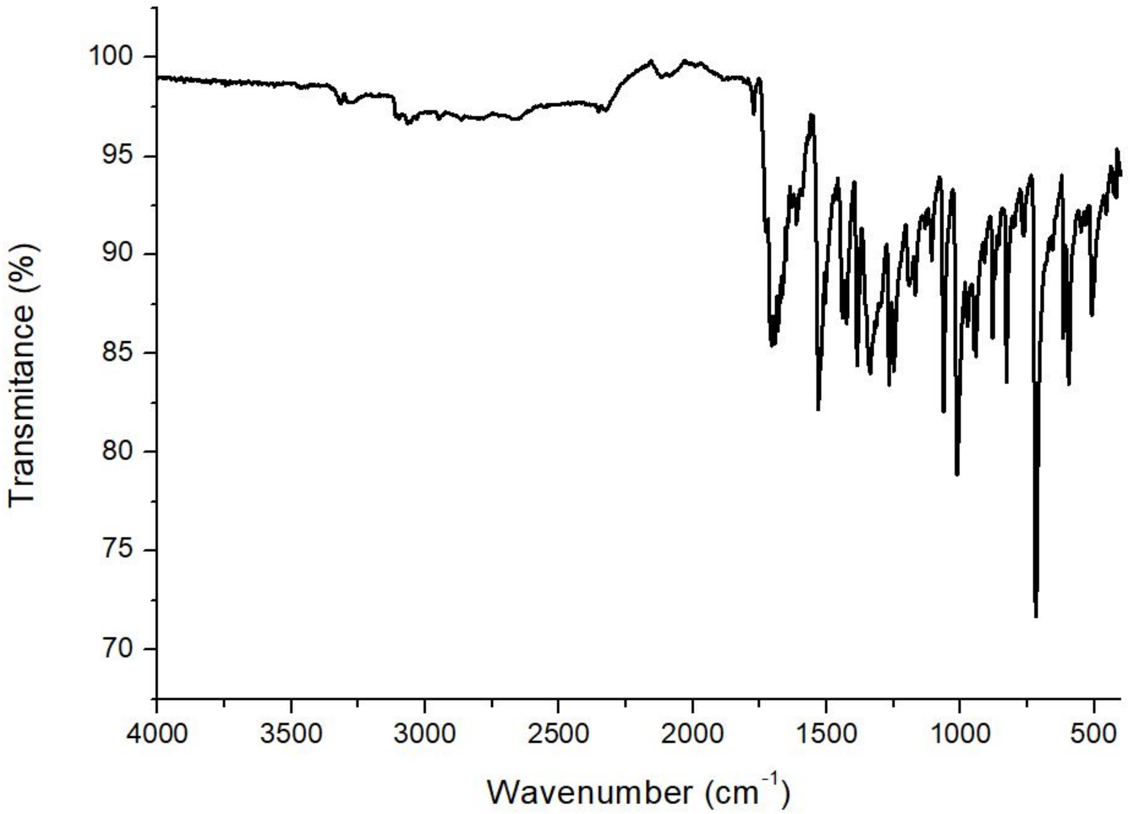

Infrared spectra of phthalimide **6**.

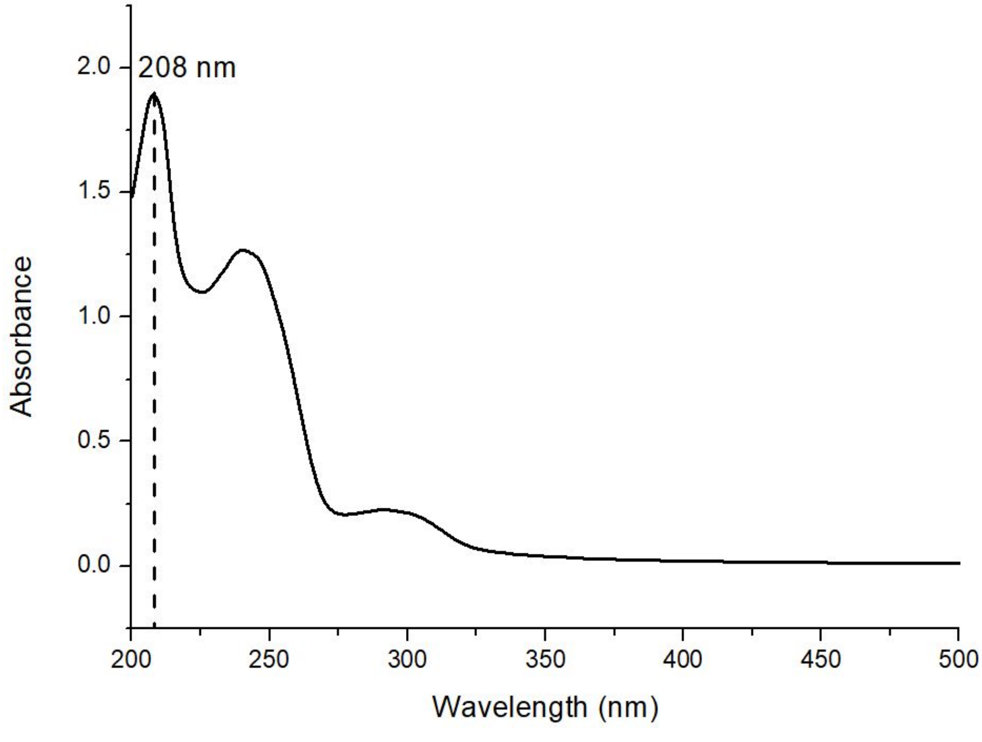

UV-Vis absorption spectra of phthalimide **6**.
